# Association of Overweight with Food Portion Size among Adults of São Paulo – Brazil

**DOI:** 10.1371/journal.pone.0164127

**Published:** 2016-10-05

**Authors:** Jaqueline Lopes Pereira, Aline Mendes, Sandra Patricia Crispim, Dirce Maria Marchioni, Regina Mara Fisberg

**Affiliations:** 1 Department of Nutrition, School of Public Health, University of São Paulo, São Paulo, Brazil; 2 Department of Nutrition, Federal University of Paraná, Curitiba, Paraná, Brazil; State University of Rio de janeiro, BRAZIL

## Abstract

**Background:**

Although studies show that portion size affects energy intake, few have demonstrated a link between portion size and weight status, especially in free-living populations. The objective of the present study was to assess the relationship between food portion sizes and overweight in a representative population of adults of São Paulo, Brazil.

**Methods:**

Cross-sectional population-based study with 1005 adults from São Paulo, Brazil. Dietary data were obtained from two 24-hour recalls. Reported foods were classified into groups and energy contribution, prevalence of consumers and portion sizes were calculated. Individuals were classified according to BMI in with and without overweight. Logistic regression models were used to evaluate the association between food portion sizes and being overweight.

**Results:**

The most consumed food groups were: beans, breads/rolls, coffee/tea, milk, rice, and sugar. Rice, red meat, breads/rolls, and white meat were the groups with the highest percentage of contribution to total energy intake. Butter/margarine, toasts/biscuits, sugar, and cakes were the groups with the highest energy density. After adjustment for confounding variables, overweight was associated with larger portions of pizza (OR = 1.052; p = 0.048), red meat (OR = 1.025; p = 0.043), rice (OR = 1.033; p<0.001), salted snacks (OR = 1.078; p = 0.022), and soft drinks (OR = 1.016; p = 0.007).

**Conclusions:**

Larger portions of few food groups with different energy densities were associated with being overweight, suggesting that overweight may be related to the consumption of larger portion sizes of a series of food groups, not a food group alone. Additionally, we highlight the importance of considering underreporting as a confounding factor in these associations.

## Introduction

Overweight has become a very important issue in the global epidemiological scenario because of the increasing rates and the association with several chronic noncommunicable diseases [1]. The etiology of overweight is complex, with several contributors, such as genetic, physiologic, environmental, psychological, social, and economic factors [2]. These influences ultimately act by changing the energy balance equation, that is, the long-term balance between the amount of energy consumed and the energy spent in everyday life [3]. In terms of energy intake, diet is one of the modifiable factors that may interfere in nutritional status of individuals. One aspect of the diet that has been related to increase in energy intake is the increase of food portion size [4], [5], [6].

Studies show a parallelism between the rise in the prevalence of overweight and the expanding portion sizes of commercially available foods [7], [8], [9] as well as the amount of food consumed per eating occasion [10], [11]. In many laboratory studies, larger portions of foods, especially those with high energy density, were associated with increase in dietary energy intake. According to those studies, large portion sizes seem to enhance food consumption and this effect can be substantial and sustained for several days [4], [12], [13].

Although numerous studies show that portion size affects energy intake, such as those cited by Rolls [14], few have demonstrated a link between portion size and weight status in adults, especially in free-living populations [15] and did not consider dietary energy density, which is a potential confounder for the relationship between food portion size and adiposity [16], [17], [18]. Therefore, the objective of the present study was to assess the relationship between food portion sizes and overweight in a representative population of adults living in São Paulo, Brazil.

## Methods

### Population and study design

Data was derived from the Health Survey of São Paulo (HS-SP; http://www.fsp.usp.br/isa-sp/), a cross-sectional population-based survey that aimed to collect health and nutrition information as well as life conditions on a representative sample of residents of the city of São Paulo, Southeastern Brazil, in 2008. The eligibility of sample study includes: to be resident of São Paulo metropolis, to be 13 years old or more and to be sorted considering the sampling design. A structured questionnaire concerning demographic, socioeconomic, anthropometric, family and lifestyle characteristics was applied by trained interviewers during a household interview. Details of this study are published elsewhere [19].

From a total of 1102 individuals aged 20 years and more who completed the questionnaire and provided dietary data, 97 individuals were excluded: 25 who did not inform weight or height (required for BMI calculation); 6 because they were taking medication to lose weight; 66 because they stated that they had changed their food habits during the study (63 intended to lose weight and 3, to gain weight). Accordingly, the final sample for this study was 1005 individuals.

HS-SP was conducted according to the guidelines laid down in the Declaration of Helsinki and all procedures involving human subjects were approved by the Human Research Ethics Committee of the Public Health School at University of São Paulo. Written informed consent was obtained from all participants who agreed to participate. The Research Ethics Committee of the Public Health School approved the present study (OF.COEP 149/12).

### Food Consumption Data

Dietary data consisted of two 24-hour dietary recalls (24HR) collected by trained interviewers over one year in non-consecutive days, randomly representing every weekday and weekend day, and season of the year. The first 24HR was obtained in participants’ homes by using the multiple-pass method [20], and a second 24HR was performed telephonically, based on the computerized version called automated multiple-pass method [21]. This method is structured in five steps designed to enhance complete and accurate food recall and reduce respondent burden [22]. Participants were instructed to inform amounts in household measures and describe them as detailed as possible, including eating occasions, meal time, cooking methods, seasonings and brand names. Quality control of the 24HR was conducted during data collection and analysis to identify and correct reporting or data entry errors, through supervision of the 24HR collection; review of the completed forms of standardized recipes, and after entering data, intending to identify outliers.

After dietary data collection and checking, all household measures of food and beverages were converted into grams or milliliters, respectively [23], [24]. Nutrition Data System for Research (version 2007, NCC, University of Minnesota, Minneapolis, USA) was used to determine nutrient content of each food and beverage. Typically Brazilian preparations which were not in the program were included using standardized recipes of regional food preparations [24], [25].

### Foods grouping and portion size

A total of 1200 different foods were registered in both 24 HR. Ad-hoc food groups were created for portion size analysis, according to their nutritional value (e.g., all types of fruits were combined into the *‘Fruits’* group), portion sizes, dietary habits and culinary usage of São Paulo population [26] (e.g., *‘Beans’* group includes brown and black beans because they are cooked legumes usually eaten with rice, whereas *‘Legumes’* group includes soybeans, lentils, and chickpeas because they are usually consumed in different preparations, such as salads). Foods and preparations consumed by less than 5% of the population, such as *feijoada*, *sushi*, *yakssoba*, and *risotto*, were not considered for grouping due to their low prevalence. These excluded foods represent 2.9% of the total foods reported.

The initial 46 food groups were evaluated to check which food groups mostly contributed to total energy intake, intending to define the food groups to be part of portion size analysis. For that, food groups that contributed with up to 90% of total energy intake were selected [27], associated with all food groups that were consumed by at least 10% of the population so as to include those food groups that did not contributed significantly to energy intake but were often consumed (e.g: leafy vegetables) (S1 Fig). Accordingly, 27 food groups were analyzed, as described in results section.

Portion size, defined as total amount of food that a person consumed at a particular eating time (in grams) [28], was established by the total intake of items included in the group and consumed in at least one 24HR, divided by the number of eating occasions of these consumed items. For example, if an individual consumed 100g of rice in lunch and 100g in dinner, his/her portion size of rice is 100g; and if another individual consumed 200g of rice in lunch and did not consume rice in any other meal, his/her portion size is 200g. When the food group was consumed in both 24HR, mean was calculated for each day and, after that, the values of each 24HR were averaged. For food groups consumed in only one 24HR, mean reflected only that consumed day. The same methodology was used in other studies, such as in the investigation about food portion size and childhood overweight [17]. These data do not reflect cumulative amount of foods consumed by individuals during the course of a day because they were examined on an individual meal basis. Thus, these were per-consumer averages, not *per capita* averages, and were intended to show changes in the average portion size for those who consume a specific item, so only individuals who consumed a certain food group were included in the analysis of this group.

### Energy density

Total daily energy density was estimated for each individual dividing the usual energy intake (EI, in kcal and kJ) of each 24HR by the usual amount consumed (in grams). EI of each 24HR was estimated according to FAO methodology [29], which is more accurate than other methods, since it considers the amount of energy that can actually be used by the organism. For example, it distinguishes the energy value of available carbohydrate (starch polysaccharides: 4 kcal/g) from the available energy of fibers (2kcal/g).

Usual EI and amount consumed were estimated using the Multiple Source Method. It is a statistical method for adjusting dietary data by within-person variability, which uses 24HR to estimate usual intake, even when a second 24HR is present only in a subsample [30]. In the present study, 539 individuals (53.6%) presented the second 24HR, which is considered adequate to estimate usual intake for all individuals [31]. Energy density was calculated in kJ per gram consumed, first for the overall intake and second separately for each food group.

### Determination of misreporting percentage

Misreporting percentage was calculated intending to reduce the impact of misreporting on the association of food portion size and overweight [15]. The determination of misreporting percentage was based on a methodology used by Kelly et al. (2009), where the equation: EI (energy intake)–EER (estimated energy requirements) /EER x 100 results in a misreporting percentage of each individual energy needs. The value of misreporting can be negative (if EI is lower than EER) or positive (if EI is higher than EER).

For determination of misreporting percentage, the EI was defined by the energy intake of the first 24HR. EER were calculated using the formulas of the Institute of Medicine of the National Academies (2002) [32], that are derived from data of energy expenditure for doubly labeled water, specific for gender and age, and based on age, height and weight of individuals, apart from using metabolic equivalent (MET) data to estimate physical activity expenditure. Data for calculating METs were obtained through the International Physical Activity Questionnaire–IPAQ [33], with questions about duration, frequency and intensity of physical activities in relation to occupation, leisure, household, and transportation. This questionnaire was validated in Brazil [34] and applied in HS-SP.

### Anthropometric Measurements

Height and weight, used to calculate the body mass index (BMI) (BMI = weight (kg)/height (m)^2^), were self-reported. The use of self-reported high and weight is known to incur in possible errors, but previous study with the same population showed good agreement between measured and self-report weight, height and BMI [35].

Individuals were classified according to their BMI into two groups: without overweight (BMI<25 kg/m^2^) and with overweight (BMI≥25 kg/m^2^), according to World Health Organization recommendations [36].

### Statistical Analysis

Stata software was used in all analysis (Statistics/Data Analysis, version 13.1, Texas, USA). Descriptive statistics were used to describe prevalence of consumers, mean, standard deviation and median of the portion (g) of each food group for consumers only.

General and dietary characteristics were described for all adults and the whole sample stratified by weight status. Differences between individuals with and without overweight were assessed using Pearson chi-square tests for categorical variables, and Mann–Whitney tests were used to assess differences between continuous non-parametric variables.

The contribution of each food group to total energy intake was assessed using the formula weighed by Block et al. (1986) [27], while the energy density of these groups was calculated dividing the total energy of each food group by its amount in grams.

Stepwise forward logistic regression models were used to evaluate the association between food portion sizes (continuous variable) and overweight (dependent variable), controlling for confounding factors. The factors that were found to be statistically significant (p<0,20) or were considered important predictors of the outcome were included in the models. The first model was a crude model of overweight and each food group (without co-variables); the second model was adjusted for age (until 60 years and 60 years or more), *per capita* family income (until one minimum salary and one minimum salary or more), physical activity level (sedentary, insufficiently active, active or very active), dietary energy density (continuous variable), and gender (female and male); and the third model was adjusted for the same co-variables of the second model with one additionally co-variable: misreporting percentage (continuous variable). All the models were well calibrated, according to Hosmer-Lemeshow goodness-of-fit test for deciles of risk [37]. OR and 95% CI were estimated for 10 grams of each food group. Associations of each food group on body mass index category were considered significant in the logistic regression models when p<0.05.

## Results

Table 1 presents characteristics of the studied population, comparing categories with and without overweight. Prevalence of overweight was 51.6% of total population. Besides presenting higher BMI (p<0.001), those with overweight were older (p<0.001). There was no difference in proportions of gender, education of householder level, income, or practice of physical activity among those with and without overweight. In relation to dietary characteristics, there was no difference in total grams intake of foods and beverages, but total energy intake (p<0.01) and energy density (p<0.01) were lower for those with overweight compared to those without overweight. Individuals with overweight presented higher percentage of misreporting (p<0.001), tending to negative values (underreporting). Eighty five percent (85%) of individuals underreported their energy intake in relation to their energy needs to some degree (from -0.2% to -86.7%), of which 80% were from those without overweight and 90% from overweight individuals.

**Table 1:** Characteristics of subjects in the Health Survey of São Paulo (2008) without and with overweight.^a^

	All adults (n = 1005)		Without OW (n = 486)		With OW (n = 519)		
	Median or n	IQR or %	Median or n	IQR or %	Median or n	IQR or %	p^b^
**Age (years)**	57	38, 69	48	31, 68	61	45, 70	0.000
**BMI (kg/m**^**2**^**)**	25.3	22.4, 28.4	22.3	20.6, 23.8	28.1	26.5, 30.8	0.000
**Gender, female (%)**	607	60.4	295	60.7	312	60.1	0.850
**Education of householder ≤ 9 years (%)**	653	65.9	312	65.0	341	66.7	0.565
***Per capita* family income ≤ 1 MW (%)**	378	37.6	199	41.0	179	34.5	0.091
*Physical activity level*							
**Sedentary (%)**	266	26.5	119	24.5	147	28.3	
**Insufficiently active (%)**	230	22.9	113	23.3	117	22.5	
**Active (%)**	328	32.6	155	31.9	173	33.3	
**Very active (%)**	181	18.0	99	30.4	82	15.8	0.210
*Dietary characteristics*							
**Total energy (kJ/d)**	6776	5480, 8313	7002	5569, 8517	6569	5383, 8076	0.007
**Total energy (kcal/d)**	1618	1309, 1986	1672	1330, 2034	1569	1286, 1929	0.007
**Total grams of foods and beverages (g/day)**	1358	1128, 1642	1385	1133, 1646	1318	1118, 1640	0.292
**Total energy density (kJ/g)**	5.0	4.5, 5.6	5.1	4.5, 5.6	4.9	4.4, 5.5	0.006
**Total energy density (kcal/g)**	1.2	1.1, 1.3	1.2	1.1, 1.3	1.2	1.0, 1.3	0.006
**Percentage of misreporting (median)**	-32.4	-51.6, -10.0	-25.9	-46.9, -3.3	-36.0	-54.9, -17.2	0.000

OW, Overweight (overweight plus obesity); IQR, Interquartile Range; MW, Minimum Wage (One MW is approximately 217 US dollars)

^a^ Without overweight: BMI<25 kg/m2; With overweight: BMI≥25 kg/m2.

^b^ Categorical variables are compared using X^2^ tests and continuous variables are compared using Mann-Whitney tests.

Food groups that contributed with up to 90% of total amount of energy and/or were consumed by at least 10% of the population are described in Table 2. The most consumed food groups (>70%) were: beans, breads and rolls, coffee and tea, milk, rice, and sugar. The contribution to total energy intake and the energy density of the food groups that contributed with up to 90% of total amount of energy are described in S1 Fig. Rice, red meat, breads and rolls, and white meat were the groups with the highest percentage of contribution to total energy intake, while butter and margarine, toasts and biscuits, sugar, and cakes were the four groups with the highest energy density.

**Table 2:** Description of food groups, food group contents, prevalence of consumers, median, mean and standard deviation of the mean of the portion (g) of each food group for consumers only.

Food groups	Description of food group contents	Consumers		Portion size		
		n	%	Median	Mean	SD
Alcoholic beverages	Fermented and distilled alcoholic beverages	119	11.8	351.5	603.4	603.5
Beans	Black and brown beans	769	76.5	86.0	102.9	66.9
Breads and rolls	White and brown bread and rolls	807	80.3	50.0	60.7	31.7
Butter and margarine	Butter and margarine	525	52.2	15.0	16.8	11.8
Cakes	Cakes with or withou topping and filling	139	13.8	60.0	75.9	44.7
Cheese	Cheeses, all types	330	32.8	30.0	36.2	25.9
Coffe and tea	Coffee and tea, caffeinated or decaffeinated	868	86.4	102.1	126.8	97.0
Cold Cuts	Ham, salami, roast beef, turkey ham	105	10.5	30.0	33.1	25.9
Eggs	Boiled, fries, scrambled eggs	149	14.8	50.0	52.0	29.5
Fruit juices	Lemonade, pinnaple, orange, passion fruit juices, etc. With or without sugar	214	21.3	240.4	258.0	149.6
Fruits	Banana, apple, papaya, melon, mango, citrus fruit, etc.	518	51.5	135.0	159.2	110.2
Industrialized juices	Ready-to-drink juices, powder juices, nectar, etc. With or without sugar	265	26.4	186.7	220.6	109.2
Leafy vegetables	Lettuce, cabbage, escarole, kale, etc.	525	52.2	30.0	40.9	35.1
Milk	Whole, lowfat, skimmed and fortified milks	721	71.7	130.0	164.1	115.3
Pasta	Spaghetti, ravioli, noodels, canelloni, lasagna, etc. With or without sauce	265	26.4	190.0	205.0	137.3
Pizza	Sweet (e.g. chocolate) or salt (e.g. mozzarella) pizza	76	7.6	154.1	186.4	116.1
Red meat	Beef, hamburger, liver, ribs, jerked beef, lamb, pork, etc.	674	67.1	90.0	101.0	75.5
Rice	White and brown rice	889	88.5	125.0	153.8	97.9
Salted snacks	Fried or baked snacks, e.g. "esfiha", "coxinha", croissant, cheese bread, etc.	143	14.2	60.0	83.9	95.3
Soft drinks	Fruit-flavored drinks, cola and noncola soft drinks, light and diet soft drinks	365	36.3	250.0	296.5	229.3
Soups	Soups and creams, including with vegetables or pasta	121	12.0	350.0	375.7	220.1
Sugar	Added sugar, white or brown	725	72.1	8.0	10.2	8.8
Sweets	Chocolate, puddings, ice creams, sweet rolls and pies, etc.	297	29.6	54.5	75.8	70.5
Toast and Biscuits	White and brown toasts, savory or sweet biscuits, with or without filling	337	33.5	26.7	34.8	33.2
Tubers and Roots	Potato, cassava, yams, sweet potato	215	21.4	85.0	116.4	120.5
Vegetables	Carrots, tomato, eggplant, broccoli, cucumber, radish, etc.	553	55.0	50.0	71.8	71.1
White meat	Chicken, turkey, fish and seafoods	472	47.0	85.3	108.1	85.1

The crude logistic regression model highlighted a positive association between the portion size of salted snacks with overweight. This positive association persisted when making adjustment of the second model. When the model was further adjusted for misreporting, we observed positive associations of pizza, red meat, rice, salted snacks, and soft drinks with overweight (Table 3).

**Table 3:** Association of each food group consumed by the total population in the Health Survey of São Paulo (2008) on body mass index category assessed using logistic regression models.

Food groups	Crude model			Adjusted model 1^a^			Adjusted model 2^b^		
	OR	95% CI	P	OR	95% CI	p	OR	95% CI	P
Alcoholic beverages	0.996	(0.990, 1.003)	0.273	0.994	(0.985, 1.003)	0.174	0.996	(0.987, 1.005)	0.397
Beans	0.986	(0.964, 1.007)	0.192	0.997	(0.973, 1.021)	0.814	1.013	(0.987, 1.038)	0.324
Breads and rolls	0.968	(0.924, 1.012)	0.151	0.980	(0.932, 1.029)	0.427	1.004	(0.953, 1.055)	0.874
Butter and margarine	0.880	(0.734, 1.029)	0.113	0.903	(0.745, 1.064)	0.238	1.018	(0.848, 1.191)	0.837
Cakes	1.002	(0.927, 1.077)	0.963	1.019	(0.934, 1.105)	0.660	1.023	(0.936, 1.111)	0.602
Cheese	1.061	(0.972, 1.015)	0.181	1.075	(0.972, 1.178)	0.154	1.082	(0.976, 1.188)	0.129
Coffe and tea	1.001	(0.987, 1.015)	0.866	1.000	(0.986, 1.015)	0.954	1.006	(0.990, 1.021)	0.453
Cold Cuts	1.042	(0.889, 1.020)	0.596	1.006	(0.844, 1.171)	0.939	1.039	(0.896, 1.216)	0.666
Eggs	0.962	(0.853, 1.073)	0.503	0.948	(0.820, 1.077)	0.426	0.972	(0.841, 1.106)	0.682
Fruit juices	1.015	(0.996, 1.034)	0.123	1.017	(0995, 1.039)	0.134	1.022	(0.999, 1.045)	0.062
Fruits	1.008	(0.992, 1.025)	0.322	1.006	(0.989, 1.023)	0.510	1.012	(0.994, 1.031)	0.180
Industrialized juices	1.007	(0.985, 1.030)	0.549	1.019	(0.993, 1.045)	0.145	1.023	(0.997, 1.050)	0.085
Leafy vegetables	1.044	(0.989, 1.099)	0.113	1.045	(0.991, 1.100)	0.105	1.050	(0.992, 1.108)	0.089
Milk	1.002	(0.990, 1.015)	0.722	0.999	(0.985, 1.013)	0.923	1.005	(0.991, 1.019)	0.502
Pasta	0.999	(0.982, 1.017)	0.922	1.000	(0.981, 1.020)	0.994	1.005	(0.985, 1.025)	0.608
Pizza	1.024	(0.983, 1.065)	0.252	1.041	(0.993, 1.088)	0.092	1.052	(1.007, 1.103)	**0.047**
Red meat	1.005	(0.985, 1.025)	0.607	1.012	(0.989, 1.035)	0.306	1.028	(1.003, 1.052)	**0.028**
Rice	1.005	(0.991, 1.018)	0.477	1.010	(0.995, 1.026)	0.186	1.031	(1.014, 1.048)	**0.000**
Salted snacks	1.058	(1.002, 1.114)	**0.041**	1.069	(1.003, 1.136)	**0.041**	1.074	(1.007, 1.141)	**0.030**
Soft drinks	1.004	(0.994, 1.013)	0.449	1.011	(1.000, 1.022)	0.053	1.016	(1.004, 1.028)	**0.009**
Soups	1.002	(0.986, 1.019)	0.792	1.000	(0.980, 1.018)	0.926	1.005	(0.984, 1.025)	0.662
Sugar	0.876	(0.706, 1.048)	0.158	0.856	(0.679, 1.035)	0.115	0.909	(0.734, 1.087)	0.313
Sweets	0.991	(0.959, 1.024)	0.600	0.994	(0.958, 1.031)	0.756	1.008	(0.969, 1.046)	0.703
Toast and Biscuits	0.964	(0.895, 1.034)	0.318	1.005	(0.931, 1.080)	0.886	1.021	(0.945, 1.097)	0.595
Tubers and Roots	1.004	(0.982, 1.027)	0.717	1.002	(0.979, 1.026)	0.843	1.009	(0.984, 1.034)	0.469
Vegetables	1.002	(0.979, 1.026)	0.849	1.007	(0.981, 1.033)	0.582	1.010	(0.984, 1.037)	0.454
White meat	0.988	(0.967, 1.010)	0.280	0.988	(0.965, 1.012)	0.332	0.995	(0.971, 1.019)	0.689

OR, estimated regression coefficient for 10 grams of each food group; 95% CI, 95% Confidence Interval

^a^ Adjusted model 1: models adjusted for age, per capita family income, physical activity level, dietary energy density, and gender.

^b^ Adjusted model 2: models adjusted for age, per capita family income, physical activity level, dietary energy density, gender, and misreporting.

## Discussion

The findings of the present study indicated positive association between overweight and portion sizes of five food groups: pizza, red meat, rice, salted snacks, and soft drinks. To our knowledge, this is the first study to evaluate the epidemiological association of overweight with portion size of food groups in Brazilian adults, taking into account potential confounders, like dietary energy density and misreporting.

In the last years, researchers have been investigating the possibility of larger portion sizes contribute to excess energy intake, and hence increase the prevalence of overweight, which reached epidemic proportions in many countries, including Brazil [38]. These studies observed that increases in portion size have occurred in parallel with the rise in the prevalence of obesity [7], [8], [9], [10], [11]. Furthermore, experimental studies have shown that providing individuals with larger food portions leads to increases in energy intake, considering that individuals tend not to compensate an increase in food intake during a meal by eating less in subsequent meals. These effects were observed in studies with one meal in one day [4], [13], but also in those with even 11 consecutive days [12]. In spite of these studies, data demonstrating a link between portion size and body weight status in free-living adults are scarce.

A study with British adults found positive association between BMI weight-status category and higher portion sizes of whole and low-fat milks, potatoes, fresh meat, breads and rolls, low-fat spreads, vegetables, fish, chips and processed potatoes and meat products [15]. Despite the distinct dietary habits, the present study also found positive association between overweight and higher portions of the previously mentioned food groups reported by adults of São Paulo (pizza, red meat, rice, salted snacks, and soft drinks). This data suggests that there is not only one food group responsible for overweight, but an increase in portion size of different foods may contribute for increase in body weight.

One important issue discussed by this British study and also by another similar study among British adolescents [39] is that underreporting could have masked the associations. In the study with adolescents, the portion sizes of a limited number of high-energy-dense foods (high-fibre breakfast cereal, cream, and high-energy soft drinks) were positively associated with higher BMI after adjusting for misreporting. When eliminating the effect of underreporting of the adolescents, larger portions of biscuits, cheese, cream, and cakes were associated with higher BMI among normal reporters; while only high-fibre breakfast cereal and high-energy soft drinks were associated with higher BMI among underreporters.

Despite the great scientific advances, the assessment of dietary consumption of populations is still a challenge. More accurate methods, such as weighing, are impracticable in studies with a very large number of individuals and the methods mostly used in studies with populations, such as 24HR, are still subject to errors that should be carefully evaluated; although they provide tools to minimize errors, for instance the Multiple Pass Method, interviewer´s training, among others. Underreporting is one of the main problems when comparing diet and overweight because it has been demonstrated that underreporting is higher in people with higher BMI [40]. Practical and statistical solutions are used to minimize these measurement mistakes and their consequences, such as the formula used in this study and others [41]. However, the challenge is even larger when we consider that foods are not underreported in the same way, namely, underreporting is frequently specific for certain foods, both in regard to consumed amount and to report or not this consumption, according to the perception of been “unhealthy” or associated to obesity [42], [43]. Therefore, one of the limitations of this study is the systematically correction of underreporting, because the estimative of the percentage of underreporting considers that all foods are equally underreported. However, up to this moment, there is no methodology in literature that considers this differentiation. This fact could be related to the reason why portion sizes of other energy-dense food groups are not associated with overweight in this study and food groups such as leafy vegetables, vegetables and fruits, which have been associated with lower BMI [44], [45], presented positive association with overweight, although they are not significant.

As like as portion size, dietary energy density is an important determinant of energy intake [16], [18]. A study with French children [17] found positive correlation between overweight and croissant-like pastries and other sweetened pastries in children after adjusting for energy density. The present study also observed positive association between overweight and high-energy dense foods, in which high consumption has been associated to overweight [46]: pizza and salted snacks, which are considered *fast-foods*, and red meat, which also has been related to weight gain [47]. Soft drinks, which are not considered energy-dense, due to their high water content, but high-sugar and nutrient-poor beverages, also have their excessive consumption associated with overweight [48]. In spite of not been considered high-energy dense food, rice presented the highest prevalence of consumption and the highest contribution to total energy intake in relation to the other food groups. In this case, eating frequency may have influenced the association of higher portion with overweight.

Besides underreporting (previously discussed), self-report of high and weight might have attenuated the association between BMI and food portion size if heavy individuals inform lower weights as compare to light ones, however the self-reported data were validated in previous study [35], which observed high intraclass correlation between self-reported and measured parameters for weight (r>0,94) and BMI (r>0,85). Agreement between measured and self-report weight, height and BMI was good, as sensitivity was >91% and specificity was >83%.

The lack of consensus regarding the definition of food portion in the scientific literature is another important limitation that may hamper our study. The term has been discussed in the international scientific community, most notably in recent decades, but the different meanings left doubts about its application. Often portion size is confused with serving size definition. In 2013, the “Symposium focusing on advances in scientific understanding of the development of healthy food portion sizes in children and their families” proposed that serving size refers to “the amount listed on the nutrition information label of foods and in dietary guidelines for consumers” while portion size refers to “the amount of food offered to or selected by adult consumers and children” [28]. This rationale was applied to the portion size definition used in this study and also in other studies [17]. Furthermore, the use of two 24HR to estimate portion size could be considered insufficient in our study, however the research was conducted with 1005 adults, which is a considerable sample.

This study has as an important strength that is to represent dietary characteristics of adult population of São Paulo, the most populous city of the Americas, destination of migrants from several regions of Brazil and other countries, which confers upon it great ethnic and cultural diversity [49]. This heterogeneity allows the evaluation of portion size in a context with different aspects that influence portion size effect, such as ethnicity, culture, education, and food security status [50]. Furthermore, besides portion sizes associations, this study also provides useful descriptive values on portion size, prevalence of consumption and energy density of food groups that can be compared to other studies and populations.

The present study found that larger portions of five food groups with different energy densities were associated with overweight, while groups with low energy density were not associated, suggesting portion size effect of a few specific food groups in this population. This fact suggests that overweight may be related to the consumption of larger portion sizes of a series of food groups, not a food group alone. Additionally, we highlight the importance of considering underreporting as a confounding factor in these associations and the need for future studies to develop methodologies to differently correct underreporting for each food type. This study cooperates in a better comprehension of the association between weight status and food portion size, an important modifiable risk factor to prevent overweight and its consequences.

## Supporting Information

S1 Fig Percentage of energy contribution (%) and dietary energy density (ED in kJ/g and kcal/g) of food groups consumed by adults in the Health Survey of São Paulo (2008). (PDF)

S1 File Data underlying the findings described in the manuscript. (PDF)
