# Supplementary material for: Association of Overweight with Food Portion Size among Adults of São Paulo – Brazil
Source: PLoS One. 2016 Oct 5;11(10):e0164127. doi: 10.1371/journal.pone.0164127 (PMC5051931; doi:10.1371/journal.pone.0164127)

S1 Fig. Percentage of energy contribution (%) and dietary energy density (ED in kJ/g and kcal/g) of food groups consumed by adults in the Health Survey of São Paulo (2008).

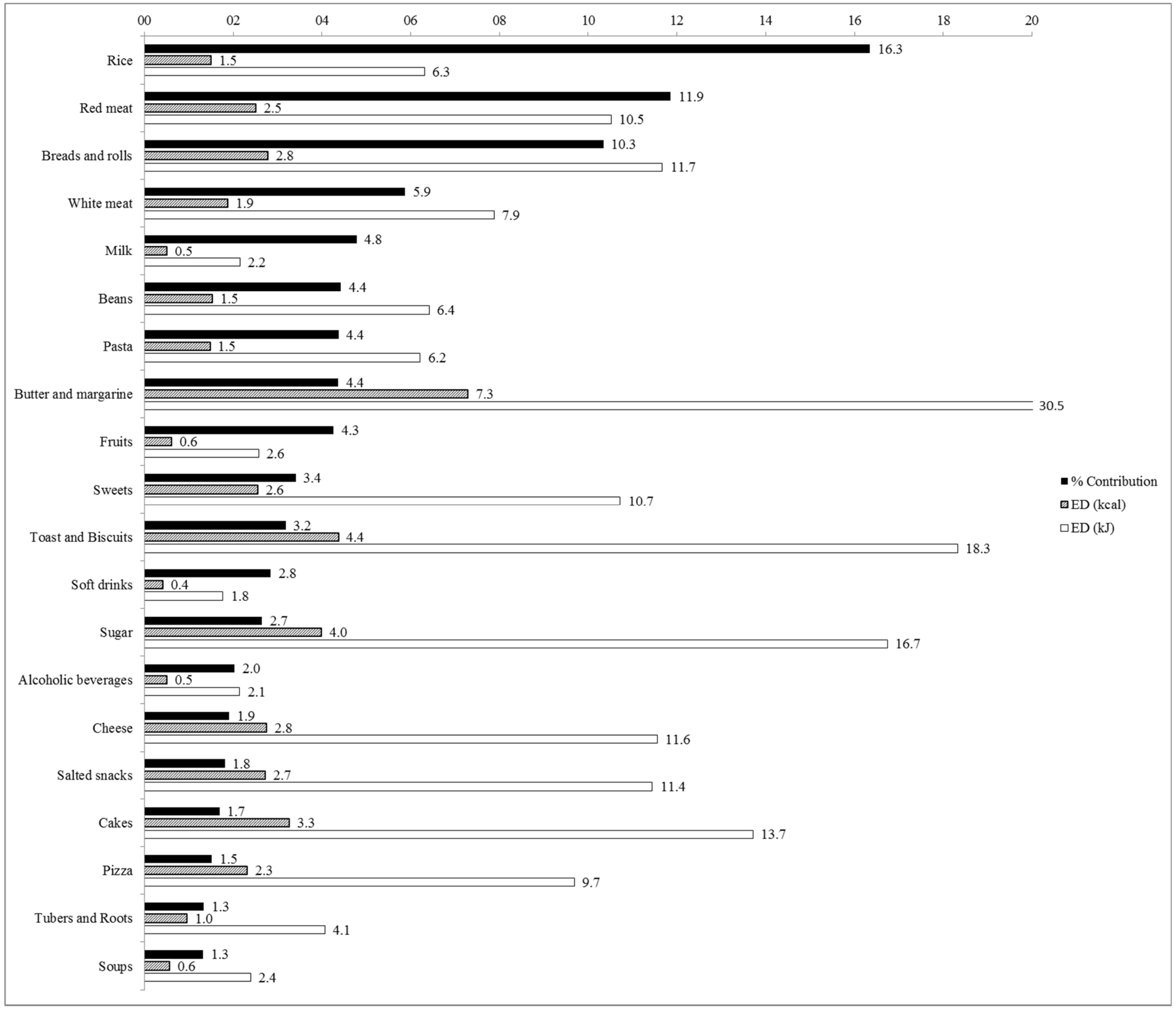

Supplement: S1 Fig — (PDF) [file pone.0164127.s001.pdf]
