# Supplementary material for: Association of Overweight with Food Portion Size among Adults of São Paulo – Brazil
Source: PLoS One. 2016 Oct 5;11(10):e0164127. doi: 10.1371/journal.pone.0164127 (PMC5051931; doi:10.1371/journal.pone.0164127)
Supplement: S1 File — (PDF) [file pone.0164127.s002.pdf]

id	age	gender	familyincome_percapita	education_householde	phisical_activity	BMI	energy_kcal_mean	energy_kcal_msm	grams_total_mean	grams_total_msm
1	54	Feminino	425.5	8	insuf. ativo	25.97012	1110.57	1299.235	1049.52	1169.696
2	57	Masculino	425.5	8	sedentário	19.9446	2820.76	2637.858	1695.57	1663.744
3	34	Feminino	425.5	8	sedentário	30.48316	2747.15	2584.86	1854.17	1777.204
4	28	Feminino	425.5	8	sedentário	21.0772	1967.35	2001.95	809.47	968.2084
5	25	Feminino	425.5	8	ativo	24.67702	1230.63	1403.085	1617.44	1606.881
6	90	Feminino	807.5	13	sedentário	31.25	1401.42	1547.355	1753.36	1705.382
7	41	Feminino	400	8	ativo	24.56747	1227.15	1400.105	648.89	825.4564
8	62	Feminino	407.1429	4	insuf. ativo	28.57796	843.01	1042.708	762.155	918.9025
9	59	Feminino	433.3333	1	ativo	20.3125	2050.115	2042.293	1457.675	1488.405
10	73	Feminino	138.3333		ativo	33.62209	1319.28	1378.581	1143.775	1064.341
11	84	Masculino		3	insuf. ativo	24.69136	2321.61	2272.01	1464.15	1493.272
12	75	Masculino	382.5	12	ativo	25.71166	2011.78	2012.328	1810.355	1711.79
13	78	Feminino	1010	4	insuf. ativo	25.23634	1698.515	1766.52	1502.915	1516.576
14	23	Masculino	275	7	ativo	21.09375	1502.24	1630.807	1348.68	1405.735
15	30	Feminino	275	7	sedentário	29.0927	1566.83	1683.656	1458.35	1488.917
16	21	Feminino	275	7	sedentário	20.76124	824.45	1042.05	800.76	960.6483
17	63	Feminino	138.3333	0	sedentário	28.125	2204.9	2181.986	1950.65	1788.086
18	75	Masculino	1192	4	ativo	23.25502	1444.855	1559.083	1161.35	1256.821
19	71	Feminino	1192	4	ativo	24.63547	1411.09	1545.934	1022.42	1127.948
20	60	Masculino	494.3333	8	muito ativo	28.32658	1206	1374.894	1790.145	1729.483
21	50	Feminino	925	13	sedentário	26.31464	1175.45	1342.043	1089.895	1200.245
22	25	Masculino	925	13	muito ativo	24.16716	620.28	848.0743	664.26	839.4511
23	28	Feminino	925	13	sedentário	21.49029	1607.87	1716.999	1161.16	1259.495
24	71	Feminino	941.3333	3	insuf. ativo	26.95313	2355.48	2297.343	1383.03	1431.963
25	51	Masculino	240	11	muito ativo	22.20408	3266.265	2941.927	2242.705	2032.581
26	43	Feminino	240	11	ativo	30.79585	1685.095	1734.638	1554.39	1535.098
27	66	Masculino	1250	4	sedentário	29.29688	1884.995	1841.222	1364.75	1390.987
28	71	Feminino	1250	4	sedentário	29.55255	1981.495	1993.226	1544.065	1552.464
29	87	Masculino		11	sedentário	21.60494	1965.665	1985.9	1644.06	1622.796
30	62	Masculino	300	0.5	ativo	24.60938	1341.65	1494.811	1544.145	1508.496
31	60	Feminino	300	0.5	sedentário	26.22222	1061.15	1255.47	1203.365	1290.242
32	39	Feminino	266.6667	11	insuf. ativo	27.53482	1524.425	1638.652	926.315	1065.034
33	40	Masculino	266.6667	11	ativo	24.97704	3280.42	2962.275	2400.66	2151.002
34	27	Masculino		4	sedentário	22.20408	1475.72	1608.971	1135.42	1238.985
35	29	Masculino		13	sedentário	23.42356	2162.28	2151.737	1258.64	1336.186
36	88	Feminino	1902.5	13	sedentário	28.13366	1502.605	1630.878	1308.41	1346.717
37	66	Feminino	1902.5	13	muito ativo	25.80645	1157.27	1339.886	877.16	1026.322
38	82	Feminino	1000	4	insuf. ativo	21.64412	591.62	819.9824	596.54	777.2004
39	84	Masculino	1000	4	sedentário	24.22145	585.96	814.4065	484.76	670.7448
40	85	Masculino	2288	13	ativo	26.98962	1621.07	1727.685	1715.93	1678.453

41	75	Feminino	2288	13	ativo	27.35884	1892.95	1943.952	2318.48	2096.292
42	63	Masculino	6050	12	sedentário	29.41076	2075.46	2077.24	2682.88	2334.702
43	61	Feminino	1750	13	ativo	27.94215	1189.5	1353.791	1089.43	1185.861
44	63	Masculino	6666.667	13	muito ativo	30.09496	1882.74	1932.344	2920.32	2375.67
45	76	Masculino	2350	12	ativo	20.57613	1935.325	1976.951	1655.575	1573.263
46	80	Masculino	375	3	sedentário	29.01745	2544.22	2437.1	1531.5	1543.535
47	67	Feminino	375	3	sedentário	28.51563	2180.19	2165.35	1609.36	1600.961
48	82	Feminino	207.5		ativo	22.04916	1858.65	1917.051	1357.72	1412.653
49	42	Feminino	112.5		ativo	28	1825.47	1890.927	1545.12	1553.632
50	73	Feminino	415	3	sedentário	31.0137	856.705	1056.665	659.78	815.3809
51	75	Feminino	830	0	insuf. ativo	25	755.405	964.2466	962.585	1086.211
52	25	Masculino	240		ativo	20.98399	2881.13	2681.097	1451.58	1483.828
53	68	Masculino	425	7	sedentário	25.86451	2067.8	2079.517	1593.24	1589.13
54	62	Feminino	425	7	ativo	32.00732	1237.56	1409.016	1026.55	1150.938
55	40	Feminino		8	insuf. ativo	20.95661	1337.43	1493.748	1182.06	1276.066
56	66	Feminino	535	1	sedentário	31.25	1352.56	1501.358	1379.185	1405.486
57	42	Masculino	535	1	ativo	29.51557	2330.04	2276.552	2062.195	1919.665
58	51	Masculino	512.3333	0	ativo	29.75207	871.19	1084.695	949.365	1080.262
59	51	Feminino	512.3333	0	ativo	26.95313	1280.99	1446.028	1108.68	1217.557
60	26	Feminino	120	8	insuf. ativo	24.98959	2384.675	2318.189	1958.825	1850.007
61	50	Masculino	1638.333	0.5	muito ativo	24.56747	3442.93	3074.502	3535.49	2864.977
62	76	Feminino	830	4	sedentário	26.34649	870.6851	1057.742	1043.63	1138.654
63	26	Masculino	457.5	12	muito ativo	23.04002	3070.04	2802.522	2732.83	2318.969
64	52	Feminino	457.5	12	insuf. ativo	26.07897	1005.265	1191.341	724.66	883.242
65	33	Masculino	720	10	insuf. ativo	26.23457	2330.63	2278.764	2336.47	2108.31
66	25	Masculino	720	10	ativo	24.9308	3117.21	2848.315	2085.37	1938.339
67	67	Masculino	507.5	6	sedentário	32.76939	1159.4	1285.731	967.325	1041.258
68	67	Feminino	507.5	6	ativo	27.70145	762.415	967.678	499.245	684.7401
69	45	Feminino	180.5556	2	insuf. ativo	22.49135	1452.705	1279.582	1666.77	1526.732
70	47	Masculino	180.5556	2	muito ativo	19.15709	2462.905	2204.562	2162.825	1765.824
71	22	Feminino	180.5556	2	ativo	24.8833	1988.435	1804.441	1624.975	1484.937
72	62	Feminino		1	insuf. ativo	20.44674	2357.14	2298.583	1429.24	1466.993
73	62	Feminino	415	3	insuf. ativo	23.72529	1332.22	1489.361	931.21	1071.958
74	48	Feminino	682.5	11	insuf. ativo	20.39542	1580.77	1695.001	1011.18	1138.329
75	39	Feminino	504.25	3	ativo	31.2213	953.9	1160.263	919.9	1062.462
76	48	Masculino	666.6667	8	ativo	25.08286	2114.05	2109.098	1945.8	1817.222
77	46	Feminino	666.6667	8	sedentário	28.13366	1207.32	1375.674	1235.93	1310.65
78	86	Feminino	682.5	0	sedentário	34.92768	1090.91	1282.02	621	799.8641
79	46	Masculino	525	11	ativo	29.06122	1685.05	1715.925	1333.44	1378.281
80	45	Feminino	525	11	ativo	36.57979	2071.69	2080.447	1891.96	1800.15
81	82	Feminino	400	4	sedentário	29.51594	982.82	1186.235	1037.75	1160.097

82	54	Feminino	300	8	muito ativo	25.91068	1398.235	1503.773	1413.705	1423.057
83	32	Masculino	300	8	ativo	26.84636	2548.3	2440.096	2549.57	2248.943
84	60	Feminino	366.6667	0.5	muito ativo	29.51594	658.19	884.8807	1147.28	1248.449
85	44	Masculino	188.3333	6	sedentário	29.71428	1438.79	1575.851	1272.535	1343.273
86	69	Feminino	475	4	insuf. ativo	30.46875	1612.53	1719.941	2056.065	1777.608
87	31	Masculino	1400	7	muito ativo	38.30317	1927.37	1970.843	1364.62	1417.926
88	67	Masculino	525	4	insuf. ativo	36.43665	1335.79	1429.123	1461.61	1476.917
89	67	Masculino	313.3333	4	sedentário	25.39063	1391.37	1532.18	1404.585	1411.52
90	64	Feminino	313.3333	4	insuf. ativo	27.34375	1184.46	1348.88	1120.605	1186.88
91	30	Feminino	1250	13	ativo	22.60027	1314.33	1474.268	1221.88	1307.443
92	49	Feminino	366.6667	8	ativo	25.07619	1316.81	1476.362	1287.29	1358.445
93	68	Feminino	415	2	insuf. ativo	18.73049	1915.835	1961.429	1785.765	1727.576
94	76	Masculino	966.4	3	sedentário	23.69146	1139.365	1306.367	940.3099	1073.223
95	24	Masculino	60	8	insuf. ativo	27.70083	1851.39	1911.343	1640.79	1623.945
96	42	Masculino	440	10	ativo	19.25703	2046.035	2035.872	1610.29	1601.319
97	50	Feminino	675	11	ativo	21.09375	1579.85	1694.253	1473.96	1500.628
98	73	Masculino	700	5	ativo	26.18775	1590.915	1702.238	1326.395	1374.294
99	76	Masculino	537.5	4	ativo	24.21875	2610.035	2430.223	1905.2	1790.866
100	72	Feminino	537.5	4	ativo	30.11242	1939.99	1980.605	1676.495	1648.427
101	83	Feminino	830	4	sedentário	25.29938	853.53	1068.898	714.03	884.2625
102	59	Feminino	450	8	muito ativo	23.63403	738.76	947.118	936.26	1074.126
103	52	Masculino	450	8	ativo	27.14304	3745.375	3273.312	2532.385	2228.605
104	50	Masculino	788.75	8	muito ativo	26.85441	2395.99	2240.965	1600.335	1536.024
105	22	Masculino	788.75	8	ativo	25.43269	2015.515	1964.21	1521.535	1535.592
106	43	Feminino	788.75	8	insuf. ativo	24.4646	1934.48	1940.136	1623.73	1593.291
107	81	Feminino	800	0	sedentário	27.34375	822.99	1040.697	609.09	788.8549
108	78	Feminino	415	4	ativo	18.94631	903.9901	1115.075	747.74	914.1981
109	68	Feminino	207.5	0	insuf. ativo	22.26563	928.06	1096.555	1126.645	1231.869
110	48	Masculino	675	12	ativo	21.41095	1005.425	1206.431	1530.51	1537.068
111	67	Feminino	966.5	8	insuf. ativo	30.86301	1645.31	1739.052	1271.585	1337.564
112	62	Feminino	207.5	5	insuf. ativo	45.78564	1133.095	1308.953	921.605	1060.127
113	49	Masculino	448	4	muito ativo	29.41176	1712.64	1801.323	1205.92	1294.898
114	56	Feminino	448	4	insuf. ativo	18.04803	1066.22	1260.313	1049.09	1169.346
115	79	Feminino	601	4	ativo	29.72108	805.305	1000.674	1340.625	1387.907
116	45	Masculino	1200	4	muito ativo	22.32143	3585.775	3135.217	2753.47	2378.176
117	35	Masculino	740	11	insuf. ativo	28.90508	3757.42	3288.401	3336.8	2744.801
118	42	Feminino	740	11	ativo	24.21229	2413.92	2340.869	1958.76	1850.69
119	79	Feminino	207.5	0.5	ativo	28.51563	880.39	1093.542	791.37	952.4761
120	60	Feminino	183.3333	4	muito ativo	26.66667	882.8	1095.746	1071.22	1187.326
121	47	Feminino	688.3333	12	sedentário	34.22222	1137.92	1322.904	761.725	906.459
122	22	Masculino	688.3333	12	sedentário	25.25951	2150.23	2135.909	2131.25	1968.691

123	21	Masculino	688.3333	12	sedentário	21.84465	3874.37	3338.8	3401.085	2760.35
124	20	Masculino	688.3333	12	ativo	21.3103	2147.82	2071.524	1932.41	1795.714
125	24	Feminino	688.3333	12	muito ativo	24.34961	1972.385	1941.446	1325.955	1386.051
126	21	Masculino	168.75	11	muito ativo	25.42001	2073.34	2064.862	1453.95	1450.445
127	64	Feminino	207.5	4	insuf. ativo	24.21875	916.965	1125.354	955.86	1066.376
128	37	Feminino	366.6667	11	ativo	22.38631	2042.685	2010.634	1564.85	1464.356
129	41	Masculino	757.5	7	ativo	23.37473	1592.955	1699.681	1467.91	1487.085
130	73	Feminino	269.1667	11	ativo	25.68371	1480.76	1613.127	1662.68	1639.888
131	52	Masculino	570.25	1	muito ativo	24.91349	3090.5	2829.54	2311.7	2091.757
132	26	Masculino	570.25	1	ativo	26.06168	3077.41	2820.326	1986	1869.665
133	59	Masculino	960	11	sedentário	30.83653	2745.4	2583.596	2680.65	2333.958
134	28	Masculino	960	11	insuf. ativo	22.40879	2852.055	2642.086	2524.81	2138.025
135	21	Masculino	960	11	muito ativo	24.4898	2575.93	2460.355	2113.63	1957.717
136	49	Feminino	960	11	ativo	29.06574	663.74	890.2366	688.38	861.2623
137	23	Feminino	960	11	muito ativo	22.40588	1848.77	1909.282	1388.94	1436.459
138	41	Feminino	987.5	4	muito ativo	23.24341	1399.76	1545.971	1671.39	1646.217
139	41	Masculino	987.5	4	ativo	20.0692	1035.81	1233.439	441.38	627.9905
140	22	Masculino	987.5	4	ativo	28.08163	2560.035	2446.635	1139.725	1214.07
141	21	Feminino	987.5	4	sedentário	20.76124	1972.84	2006.211	650.8	827.1996
142	54	Feminino	645	0	insuf. ativo	33.16327	1225.87	1387.681	985.63	1114.994
143	64	Feminino	100	5	ativo	17.3076	1347.595	1499.673	1171.5	1260.378
144	67	Feminino	600	4	ativo	27.34375	1493.88	1623.932	1212.68	1300.216
145	26	Feminino	214.4286	2	ativo	20.54569	2945.055	2641.258	2070.775	1892.829
146	36	Masculino	125	11	ativo	18.09161	2619.67	2467.835	2167.485	1978.271
147	27	Feminino	125	11	muito ativo	20.3125	1878.125	1928.397	1382.5	1421.685
148	56	Feminino	375	8	muito ativo	20.77562	1064.565	1258.404	1005.045	1133.193
149	48	Masculino	832	11	ativo	26.3958	2637.04	2504.994	2124.95	1965.461
150	46	Feminino	832	11	muito ativo	24.00549	2208.73	2178.812	1770.47	1697.608
151	23	Feminino	832	11	muito ativo	22.18935	1549.57	1470.705	1251.625	1162.427
152	28	Feminino	366.6667	11	insuf. ativo	25.29938	1951.94	1984.931	1652.27	1632.088
153	41	Masculino	1240	11	muito ativo	29.40779	4797.59	3942.63	2994.05	2502.209
154	35	Masculino	500	11	muito ativo	30.93044	2421.38	2346.408	1549.29	1556.719
155	78	Feminino	375	4	sedentário	32.05128	1039.395	1235.857	1109.56	1216.739
156	66	Masculino	600	4	sedentário	23.4375	660.295	856.0042	668.465	839.4934
157	44	Masculino	523	8	ativo	26.87868	908.7599	1119.414	1241.21	1322.584
158	68	Masculino	1415	4	ativo	22.07031	2050.575	1948.708	1551.315	1390.002
159	72	Feminino	1415	4	insuf. ativo	22.07108	847.175	1058.475	858.91	993.1702
160	41	Masculino	3000	11	ativo	22.14533	3211.12	2892.292	2961.735	2459.862
161	77	Feminino	606.6667	8	insuf. ativo	23.49524	1314.27	1469.486	1297.615	1351.104
162	56	Masculino	1300	12	ativo	26.19619	2338.245	2278.938	2172.45	1997.376
163	29	Masculino	1300	12	ativo	24.37673	1477.23	1610.216	1236.22	1318.681

164	35	Feminino	1300	12	insuf. ativo	19.31295	1565.54	1682.342	982.63	1114.751
165	28	Feminino	780	7	muito ativo	25.39063	2055.655	2067.014	1745.855	1698.552
166	61	Masculino	216.6667	4	ativo	25.14861	1317.53	1476.97	1316.36	1380.906
167	70	Feminino	298.3333	0	sedentário	33.02508	1702.945	1771.496	1419.655	1459.737
168	61	Feminino	403	11	ativo	47.10569	1486.3	1551.677	821.225	910.1564
169	64	Masculino	1115	2	sedentário	18.00554	1628.65	1733.813	1636.06	1620.494
170	30	Feminino	590	11	muito ativo	20.57613	1297.825	1455.339	782.48	935.9101
171	44	Masculino	433.3333	7	ativo	32.74416	2684.82	2539.738	2129.83	1968.796
172	42	Feminino	433.3333	7	muito ativo	28.36035	1380.58	1529.953	934.28	1074.53
173	42	Feminino	158.75	3	insuf. ativo	23.4375	1226.64	1347.459	962.72	1063.129
174	61	Feminino		2	sedentário	21.96712	1191.74	1369.68	873.05	1022.825
175	63	Masculino	175	4	muito ativo	26.81359	1304.77	1466.185	1332.16	1393.063
176	45	Feminino		0	muito ativo	43.31414	1255.82	1424.609	1690.99	1660.429
177	32	Feminino	280	8	insuf. ativo	26.66667	885.31	1098.04	742.5	909.566
178	34	Masculino	186	5	ativo	26.72993	2911.11	2702.496	2254.09	2053.081
179	33	Feminino	186	5	muito ativo	21.55102	1575.85	1690.999	1197.6	1288.342
180	79	Masculino	415	1	sedentário	25.64892	686.155	911.7598	476.76	660.968
181	70	Feminino	415	1	sedentário	31.88776	1989.18	2018.878	1199.61	1289.927
182	42	Masculino	172	4	ativo	20.2449	1347.79	1502.462	1505.55	1524.235
183	35	Feminino	172	4	sedentário	29.38476	1186.76	1365.386	842.81	996.9706
184	47	Feminino	621.6667	6	ativo	22.47659	1676.57	1772.418	1367.45	1420.087
185	71	Masculino	481.6667	0	ativo	17.00882	1090.98	1282.082	1033.24	1156.412
186	64	Feminino	481.6667	0	muito ativo	28.8	449.98	677.3859	539.7	723.6912
187	69	Masculino	762.5	4	ativo	23.2438	2969.17	2743.798	2523.57	2231.95
188	40	Masculino	150	11	muito ativo	32.5771	1952.4	1990.334	2682.23	2334.976
189	37	Feminino	150	11	ativo	24.08822	1997.71	2025.481	1959.85	1851.451
190	32	Feminino			ativo	29.31986	1063.37	1257.791	741.73	908.3748
191	74	Feminino	800	4	insuf. ativo	22.89282	1113.41	1295.767	1557.06	1517.881
192	20	Masculino	750	11	sedentário	27.42857	4512.14	3786.178	2505.17	2219.896
193	68	Feminino	300.3333	4	insuf. ativo	21.45727	1473.985	1607.51	1331.665	1379.705
194	84	Masculino	285	2	sedentário	27.04164	1684.415	1761.219	1524.535	1538.236
195	81	Feminino	285	2	insuf. ativo	20.82999	1307.735	1468.627	1341.805	1388.492
196	40	Feminino	316.6667	8	muito ativo	24.97399	2079.72	2088.667	2047.8	1912.474
197	31	Feminino	200	6	sedentário	19.53125	1631.42	1736.051	1831.48	1761.126
198	28	Feminino	160	11	muito ativo	31.18459	1133.18	1318.958	630.51	808.6199
199	60	Feminino	415	2	insuf. ativo	23.18339	1101.41	1291.222	1137.76	1240.854
200	49	Feminino	505	8	insuf. ativo	24.00549	1657.44	1725.445	1715.455	1606.859
201	31	Masculino	433.3333	8	ativo	29.5858	1083.885	1274.63	947.31	1085.301
202	80	Feminino	59.28571	11	sedentário	21.79164	926.485	1113.893	973.345	1097.201
203	64	Feminino		1	ativo	16.40625	1145.955	1329.87	851.48	1001.036
204	33	Masculino	600	10	insuf. ativo	21.22634	1921.44	1966.218	1747.87	1701.441

205	50	Masculino	612	8	ativo	26.89232	2032.9	2052.661	1211.63	1299.391
206	21	Masculino	563	3	ativo	20.57143	2857.6	2664.268	2477	2201.4
207	42	Masculino	200	0	muito ativo	24.21875	1929.72	1972.675	1408.13	1451.026
208	78	Masculino		4	sedentário	29.75778	825.13	1042.68	1202.12	1291.905
209	47	Masculino	700	11	muito ativo	26.3656	709.12	933.7317	533.73	717.9986
210	42	Feminino	700	11	insuf. ativo	27.68878	1226.46	1399.514	1079.65	1194.151
211	58	Masculino	933.3333	3	sedentário	24.91349	1865.245	1917.335	1455.09	1486.129
212	59	Feminino	933.3333	3	insuf. ativo	24.45606	1762.5	1834.618	1488.42	1511.415
213	25	Feminino	933.3333	3	sedentário	21.51386	1992.89	1880.885	1666.94	1610.772
214	43	Feminino	300	11	insuf. ativo	23.61275	896.84	1108.149	998.915	1125.166
215	66	Masculino	368.8	12	sedentário	35.55556	1946.61	1983.332	1411.115	1450.151
216	49	Masculino	366.6667	10	sedentário	25.10239	3001.105	2755.99	1936.56	1834.907
217	20	Masculino	366.6667	10	sedentário	22.15265	1908.73	1892.365	1651.91	1593.961
218	47	Feminino	366.6667	10	ativo	21.28743	1246.415	1361.731	1025.035	1145.651
219	73	Feminino	263.75	3	insuf. ativo	24.21875	1332.35	1487.688	1141.105	1243.517
220	40	Masculino	390	11	ativo	28.3737	2483.2	2392.172	1999.89	1879.316
221	35	Feminino	390	11	insuf. ativo	22.03857	1492.365	1568.867	1176.48	1268.702
222	20	Feminino	627.6667	7	sedentário	21.78649	3154.89	2874.74	2009.4	1885.913
223	53	Masculino	1717.5	0.5	muito ativo	19.66231	1553.075	1671.05	1518.965	1529.186
224	52	Feminino	1717.5	0.5	muito ativo	20.56933	1284.865	1442.45	1335.415	1371.727
225	80	Feminino	420	0	sedentário	23.7037	881.38	1094.447	1298.97	1367.485
226	53	Feminino	611.3333	8	insuf. ativo	22.21368	972.69	1176.89	1393.36	1418.498
227	48	Masculino	333.3333	8	ativo	23.10843	1629.725	1679.421	1118.665	1186.971
228	25	Masculino	333.3333	8	sedentário	27.71768	1256.56	1425.153	1117.29	1206.316
229	42	Feminino	333.3333	8	insuf. ativo	22.65625	1032.91	1214.083	935.655	1038.019
230	21	Feminino	333.3333	8	sedentário	20.70082	1515.01	1641.292	967.92	1102.589
231	77	Masculino	164	4	sedentário	23.23346	772.08	980.8644	738.825	889.4383
232	81	Feminino	164	4	sedentário	24.7666	671.94	896.1847	672.575	839.7427
233	58	Masculino	334.2857	3	ativo	33.20313	1876.605	1928.697	1551.995	1528.104
234	51	Feminino	334.2857	3	sedentário	27.92667	1496.01	1607.72	1449.1	1471.881
235	31	Feminino	334.2857	3	ativo	21.48438	2513.48	2414.497	2020.3	1893.466
236	21	Feminino	334.2857	3	sedentário	17.6693	1976.7	2009.205	1654.54	1633.966
237	61	Feminino	320.6667	8	sedentário	25.33333	1360.39	1513.042	1058.22	1176.775
238	43	Feminino	59.42857	4	sedentário	16.79688	1063.2	1257.651	710.68	881.2697
239	38	Feminino	345.7143	8	muito ativo	32.95068	1671.375	1702.181	1375.925	1392.878
240	65	Masculino	2950	10	muito ativo	23.71185	2005.665	2030.025	1860.38	1781.079
241	31	Feminino	200	11	muito ativo	35.15625	1109.87	1298.624	1620.98	1609.472
242	62	Masculino	324.8	4	sedentário	17.30104	1390.175	1523.031	885.46	1033.284
243	48	Masculino	1618.75	9	ativo	25.60554	2529.425	2408.436	1889.74	1795.644
244	22	Masculino	1618.75	9	muito ativo	23.87543	2923.62	2711.151	1971.12	1766.039
245	60	Feminino	350	11	sedentário	29.0006	1475.51	1608.798	1297.14	1366.07

246	74	Feminino	82.4	13	ativo	23.11111	1332.56	1487.147	1596.45	1590.652
247	27	Masculino	975	10	muito ativo	24.60973	2834.535	2647.217	2642.28	2303.838
248	30	Feminino	103.75	2	sedentário	20.76124	1448.79	1563.343	1245.845	1325.196
249	61	Masculino		9	ativo	22.86237	1314.03	1473.714	1499.005	1517.428
250	21	Feminino	235	11	muito ativo	24.13602	2046.03	2062.776	1675.98	1649.549
251	62	Feminino	333.3333	5	sedentário	25.29938	1410.935	1555.243	1312.225	1370.603
252	58	Feminino	30	10	muito ativo	21.63115	2624.815	2494.024	1969.825	1856.804
253	79	Feminino	500	4	sedentário	26.02617	2080.075	1943.814	2277.82	1814.02
254	71	Masculino	440	12	sedentário	24.22145	800.61	1018.502	779.885	942.2878
255	87	Feminino	140	3	sedentário	23.11111	1747.67	1829.272	1655.29	1634.512
256	92	Feminino	560	6	sedentário	23.50781	594.13	822.4521	849.34	1002.572
257	57	Masculino	135.5556	3	ativo	25.90431	978.48	1181.99	899.465	1045.212
258	30	Feminino	135.5556	3	ativo	22.06035	1057.21	1252.25	1251.645	1324.272
259	79	Masculino	522.5	4	sedentário	25.34435	1383.93	1531.676	1355.245	1410.319
260	69	Feminino	522.5	4	sedentário	25.33333	1278.7	1435.112	1046.285	1165.471
261	49	Masculino	1700	11	sedentário	26.3656	2333.245	2267.915	1939.225	1826.452
262	46	Feminino	433.3333	8	muito ativo	20.28123	1969.825	2001.974	1605.945	1594.168
263	64	Feminino	600	8	ativo	24.14152	821.295	1038.914	1173.965	1261.249
264	62	Masculino	335	5	ativo	24.16327	1634.15	1733.276	1489.83	1472.219
265	77	Masculino	1166.667	13	ativo	23.73866	2017.8	2034.337	1619.835	1594.969
266	75	Feminino	1166.667	13	insuf. ativo	32.03125	777.515	995.4204	928.185	1068.921
267	64	Masculino	1564	4	ativo	19.26717	1747.76	1829.344	1834.27	1763.106
268	61	Feminino	1564	4	sedentário	30.48316	1199.17	1376.079	1124.05	1229.889
269	37	Feminino		13	muito ativo	20.70313	1206.705	1355.048	1507.68	1467.631
270	41	Masculino		13	muito ativo	23.12062	1383.05	1532.019	1477.3	1503.13
271	67	Masculino	206	7	insuf. ativo	26.53376	2347.4	2291.307	2631.94	2302.492
272	34	Feminino	2500	13	sedentário	25.65437	898.59	1102.793	1085.915	1150.232
273	61	Masculino	700	11	sedentário	28.40055	1393.445	1539.376	1479.825	1491.966
274	62	Masculino		11	muito ativo	29.04657	1600.74	1711.219	923.3	1065.32
275	53	Masculino	1333.333	11	muito ativo	26.67487	1150.16	1333.719	646.47	823.246
276	54	Feminino	1333.333	11	ativo	23.30906	1327.795	1481.29	1201.51	1284.522
277	30	Feminino	1333.333	11	insuf. ativo	17.21109	1312.89	1473.051	1274.01	1348.143
278	77	Masculino	415	11	ativo	27.92969	1825.88	1891.25	2140.87	1976.333
279	50	Feminino		12	sedentário	24.97704	825.615	1040.281	817.085	899.2775
280	61	Feminino	433.3333	12	insuf. ativo	23.37258	1506.02	1633.912	875.68	1025.063
281	30	Feminino	2000	13	insuf. ativo	27.68166	1420.13	1562.934	1145.82	1247.285
282	77	Feminino	566.6667	5	insuf. ativo	36	1038.44	1235.77	1024.85	1149.546
283	69	Feminino	253.75	5	ativo	33.29865	1366.9	1494.317	864.905	1011.086
284	57	Feminino	400	4	ativo	20.44444	884	1096.843	840.6	995.0725
285	64	Masculino	915	8	sedentário	28.22839	1216.21	1390.724	1116.16	1223.564
286	77	Feminino	457.5	1	insuf. ativo	24.88889	1103.065	1280.739	1070.735	1179.179

287	71	Feminino	415	1	insuf. ativo	27.34375	833.41	1050.341	1543.46	1552.403
288	56	Feminino			insuf. ativo	18.75	1072.03	1265.43	965.34	1100.446
289	67	Feminino	3000	4	ativo	33.29865	1885.67	1938.251	1426.24	1464.728
290	64	Feminino	166.6667		muito ativo	28.125	1014.04	1214.105	584.98	766.4156
291	70	Masculino	600	11	sedentário	21.04805	2111.1	2112.701	1627.2	1614.021
292	75	Masculino	252.5	7	sedentário	27.54821	1934.755	1970.964	1738.51	1686.133
293	65	Feminino	252.5	7	sedentário	23.87512	1741.145	1805.303	1270.85	1318.887
294	64	Feminino	665.5	1	sedentário	29.38476	1537.895	1653.179	1127.97	1232.328
295	32	Feminino		3	insuf. ativo	17.71542	984.62	1187.846	865.78	1016.629
296	70	Feminino	1707.5	8	sedentário	28.76397	1742.62	1818.865	1481.235	1498.773
297	50	Masculino	175	2	insuf. ativo	22.58955	2024.23	2039.99	1820.47	1753.13
298	48	Feminino	175	2	sedentário	30.48669	4475.645	3702.211	3318.32	2607.042
299	54	Feminino	600	2	insuf. ativo	19.81785	1650.21	1745.94	1545.015	1543.672
300	48	Feminino	833.3333	4	insuf. ativo	28.39872	1316.39	1462.073	1118.81	1212.007
301	71	Feminino	266.6667	3	insuf. ativo	31.21748	1290.295	1453.383	1020.435	1145.424
302	38	Masculino	500	11	ativo	23.98687	2604.86	2481.516	1639.26	1622.829
303	32	Feminino	500	11	ativo	24.56747	2046.705	2021.963	1156.345	1247.197
304	29	Masculino	376	4	muito ativo	22.9854	1787.86	1861.193	1666.25	1642.483
305	32	Feminino	376	4	ativo	24.23823	1675.19	1733.833	854.55	1006.979
306	60	Feminino		2	sedentário	22.22222	1168.775	1336.685	823.34	938.5187
307	24	Feminino	225	7	ativo	27.43484	2749.26	2318.115	1210.28	1254.934
308	46	Feminino	83	2	ativo	21.48438	954.78	1140.211	731.11	863.1335
309	61	Masculino	666	2	ativo	25.28257	2364.73	2304.248	2389.05	2143.302
310	23	Masculino	675	6	ativo	20.44914	1848.46	1909.038	1815.6	1749.844
311	20	Masculino	675	6	ativo	22.30935	1387.27	1535.546	1230.67	1314.335
312	75	Masculino	276.6667	0.5	sedentário	26.6782	484.58	712.8428	461.59	648.0192
313	21	Feminino	666.6667	10	ativo	20.93664	1417.76	1560.963	1645.69	1627.519
314	46	Feminino	253.75	6	ativo	21.33333	930.38	1139.027	720.64	890.158
315	91	Masculino	1150	10	sedentário	29.05475	1545.54	1666.287	1439.85	1474.997
316	91	Feminino	1150	10	sedentário	23.71185	1527.8	1651.776	1609.4	1600.991
317	72	Masculino	1107.5	11	ativo	23.4375	1837.045	1887.841	1536.335	1544.197
318	82	Feminino	1107.5	11	insuf. ativo	24.44444	1078.5	1221.303	1067.775	1149.234
319	35	Feminino	710	13	ativo	25.4017	2207.73	2186.235	1918.28	1822.369
320	35	Feminino	710	13	ativo	24.39106	1083.36	1275.393	1043.07	1164.439
321	80	Masculino	6400	11	ativo	29.80277	1311.11	1435.723	1608.19	1566.151
322	49	Feminino	300	4	ativo	22.22222	1458.65	1579.986	1060.17	1177.403
323	85	Feminino	415	4	sedentário	22.7583	1491.2	1621.726	1873.6	1790.933
324	78	Masculino	760	4	sedentário	27.35884	1540.11	1661.849	1543.28	1552.269
325	79	Feminino	760	4	muito ativo	20.82999	2355.26	2297.179	1946.19	1841.911
326	50	Feminino	433.3333	13	ativo	20.19509	1090.025	1279.569	965.33	1095.697
327	91	Masculino	1000	10	insuf. ativo	20.76124	1598.88	1679.734	1543.21	1492.144

328	84	Feminino	1000	10	insuf. ativo	25.40282	1853.06	1912.574	1825.87	1740.974
329	55	Feminino	550	11	muito ativo	30.0215	1423.785	1495.452	781.105	922.4834
330	31	Feminino	550	11	muito ativo	22.9854	2347.94	2291.71	1160.35	1258.851
331	62	Feminino	2205	10	muito ativo	34.54735	1296	1458.76	1085.34	1198.751
332	72	Feminino	415	4	ativo	24.4646	1382.67	1531.701	926.65	1068.133
333	81	Feminino	1050	11	insuf. ativo	27.94215	1185.64	1364.42	916.32	1059.451
334	50	Masculino	3500	13	muito ativo	23.5102	1313.86	1473.871	1827.33	1758.18
335	64	Feminino	240	8	muito ativo	23.18339	2134.66	2130.696	1388.2	1435.896
336	65	Masculino	3315	11	muito ativo	31.67347	1423.85	1563.198	1260.435	1327.886
337	64	Feminino	3315	11	ativo	23.95123	1026.16	1224.879	1166.84	1264.006
338	68	Masculino	1333.333	11	ativo	25.60554	1147.525	1325.698	1201.43	1279.392
339	39	Masculino	500	11	muito ativo	27.7551	1199.44	1375.927	789.7	938.8343
340	39	Feminino	500	11	muito ativo	25.88758	1690	1756.724	1255.77	1310.465
341	72	Masculino	857.5	0.5	sedentário	22.86254	2067.895	2079.427	1572.075	1571.424
342	65	Feminino	857.5	0.5	insuf. ativo	22.76944	1352	1503.851	1494.795	1513.894
343	27	Masculino	569	8	ativo	24.22145	4750.57	3939.413	2891.52	2468.537
344	24	Masculino	569	8	sedentário	22.14533	3078.66	2821.206	2352.86	2119.239
345	53	Feminino	569	8	insuf. ativo	24.00549	1631.53	1736.14	1221.95	1307.498
346	97	Masculino	674.6667	3	sedentário	17.30104	975.64	1179.801	1030.06	1153.811
347	41	Masculino		13	sedentário	28.71972	1894.35	1937.666	1370.305	1416.551
348	40	Feminino		13	insuf. ativo	18.64535	1698.91	1788.725	1077.235	1180.305
349	27	Feminino	476	7	sedentário	26.42357	2978.77	2750.61	3067.49	2578.931
350	23	Masculino	476	7	sedentário	19.11075	3952.71	3419.202	3430.27	2801.558
351	32	Feminino	200		insuf. ativo	24.03461	1625.18	1731.008	3367.94	2763.755
352	21	Feminino	200		sedentário	18.73278	2747.89	2585.394	2343.6	2113.067
353	65	Feminino	207.5	0	insuf. ativo	36.79017	931.445	1139.903	950.175	1086.847
354	61	Feminino	457.5	11	muito ativo	26.7094	678.71	893.6019	660.085	830.0723
355	29	Masculino	533.3333	11	ativo	32.87197	1591.58	1699.777	1047.78	1163.498
356	27	Feminino	533.3333	11	insuf. ativo	20.20202	918.49	1124.651	767.205	919.687
357	35	Masculino	167.125	8	ativo	19.94321	2723.67	2567.889	2172.72	1998.025
358	55	Feminino	167.125	8	muito ativo	31.23941	1253.83	1422.912	1083.44	1197.216
359	48	Masculino	509.2	6	sedentário	26.14928	1858.265	1831.127	1220.295	1255.282
360	45	Feminino	509.2	6	sedentário	23.86359	1704.58	1789.419	1203.115	1272.906
361	22	Feminino	509.2	6	insuf. ativo	19.81405	2211.65	2184.911	1522.42	1534.082
362	21	Feminino	509.2	6	insuf. ativo	23.82367	1763.13	1716.574	877.145	946.3934
363	63	Masculino	230	1	insuf. ativo	32.49055	2324.745	2274.308	1392.645	1437.118
364	38	Feminino	165	9	insuf. ativo	23.72529	928.14	1136.999	665.63	840.6948
365	81	Feminino	232.5	0	insuf. ativo	37.61574	1059.005	1230.327	901.205	1012.775
366	41	Masculino	208.3333	6	ativo	29.35752	2082.315	2089.898	2379.62	2123.375
367	41	Feminino	208.3333	6	sedentário	20.95727	1939.03	1957.998	1568.885	1566.147
368	68	Feminino	525	1	insuf. ativo	26.87817	547.995	775.2935	678.71	849.6779

369	41	Masculino	112.5	5	muito ativo	17.63085	1489.83	1620.599	2629.97	2301.217
370	40	Feminino	112.5	5	ativo	19.37716	1395.95	1542.793	1060.89	1178.945
371	73	Feminino	465	5	insuf. ativo	29.6875	754.62	976.1591	833.03	975.0731
372	23	Feminino	350	11	insuf. ativo	19.53125	3908.04	3389.414	2938.03	2497.878
373	29	Masculino	1666.667	11	sedentário	25.05931	1051.955	1247.508	775.725	935.6463
374	52	Masculino	500	11	ativo	26.1752	841.36	1057.683	981.8	1114.098
375	46	Feminino	500	11	sedentário	39.47681	1278.33	1417.155	1103.39	1205.802
376	63	Masculino	465	1	sedentário	21.30395	382.23	606.6334	654.27	830.3636
377	62	Feminino	465	1	ativo	24.03461	889.89	1102.223	913.36	1056.958
378	30	Feminino	800	5	muito ativo	29.38476	1804.37	1871.797	1402.13	1445.766
379	70	Feminino	443.3333	3	muito ativo	16	1436.93	1576.885	1509.07	1526.858
380	44	Masculino	276.6667	3	muito ativo	26.12245	3291.93	2882.997	3047.435	2539.214
381	62	Masculino	371.6667	4	sedentário	22.72044	1796.565	1863.241	1658.45	1587.915
382	78	Masculino	200	4	insuf. ativo	23.03005	2013.72	1847.434	1070.425	1148.261
383	88	Feminino	200	4	sedentário	20.88889	1154.85	1330.234	689.375	862.0856
384	34	Feminino	400	12	muito ativo	33.49768	3603.71	3184.377	2330.07	2104.037
385	63	Feminino	805	4	insuf. ativo	35.84079	1176.67	1356.307	1385.34	1432.942
386	64	Masculino	707.5	4	sedentário	32.32323	1341.48	1478.585	925.745	1041.516
387	26	Masculino	1166.667	12	insuf. ativo	23.67126	2356.5	2298.105	1876.43	1792.93
388	28	Masculino	17.14286	4	insuf. ativo	23.87512	1829.065	1893.426	1078.345	1186.174
389	53	Feminino	17.14286	4	muito ativo	24.16716	1239.845	1410.166	1041.57	1163.167
390	56	Feminino	1032.5	4	insuf. ativo	27.33991	779.01	985.7531	703.81	853.4991
391	65	Feminino			insuf. ativo	37.09869	459.42	687.1028	697.27	869.2562
392	46	Feminino		13	sedentário	21.79931	868.37	1071.485	636.89	814.3367
393	62	Masculino	266.6667	4	ativo	25.51021	1749.89	1831.04	1393.09	1439.613
394	74	Feminino	400	0.5	sedentário	24.4646	1072.18	1252.099	842.88	990.0171
395	22	Masculino	500	11	muito ativo	23.67126	2620.09	2492.635	2624.03	2297.369
396	57	Feminino	337.5	11	ativo	30.0621	1314.62	1465.481	1015.13	1115.353
397	35	Masculino	250	5	ativo	28.40551	1764.85	1839.146	1236.955	1317.536
398	40	Feminino	250	5	insuf. ativo	26.95313	1757.435	1778.35	1679.035	1571.409
399	75	Masculino	310	0.5	sedentário	25.55933	965.33	1170.546	1004.53	1132.86
400	67	Feminino	310	0.5	sedentário	23.71185	1350.51	1504.748	1007.05	1134.933
401	81	Feminino	3500	11	ativo	27.39226	687.875	899.7443	788.875	944.1064
402	49	Feminino	116.6667	6	muito ativo	29.66655	1382.46	1523.984	1092.3	1187.538
403	20	Feminino	116.6667	6	ativo	30.47797	2599.485	2471.245	2047.82	1896.329
404	63	Masculino	306.6667	11	muito ativo	23.18339	1901.825	1950.683	1308.67	1374.163
405	30	Masculino	1760	11	muito ativo	27.77427	1352.03	1506.025	1874.5	1791.568
406	27	Feminino	1760	11	muito ativo	21.875	1802.98	1871.266	1341.275	1350.581
407	79	Feminino	1833.333	4	sedentário	17.56965	1209.655	1384.502	884.22	1030.272
408	78	Masculino	310	4	sedentário	28.08163	4100.74	3517.384	2982.87	2526.053
409	76	Feminino	310	4	ativo	31.25	1992.8	2021.681	1191.67	1283.662

410	68	Masculino	207.5	4	muito ativo	28.40816	1853.525	1875.319	1480.87	1489.699
411	62	Masculino	700	13	ativo	24.4418	1543.55	1664.661	2154.38	1985.544
412	58	Feminino	1700	13	ativo	31.14187	2222.7	2195.698	2195.665	1987.822
413	71	Masculino		4	ativo	22.22222	1534.33	1657.121	1073.64	1189.287
414	68	Feminino		4	insuf. ativo	29.34129	1534.33	1657.121	1073.64	1189.287
415	39	Feminino	140	11	ativo	27.0538	1321.64	1479.436	1581.92	1580.787
416	48	Masculino	466.3333	10	ativo	24.91349	1043.095	1199.657	977.325	1067.065
417	26	Masculino	466.3333	10	ativo	28.72738	2418.465	2290.548	1963.25	1836.265
418	21	Feminino	466.3333	10	ativo	24.12879	897.9	1109.529	769.79	933.6073
419	37	Feminino	850	13	insuf. ativo	23.30668	2162.96	2152.254	1402.11	1446.461
420	79	Feminino	138.3333	2	insuf. ativo	21.48438	1071.65	1265.096	1029.34	1153.222
421	62	Masculino	703	4	ativo	25.40282	1222.715	1284.807	1379.205	1248.792
422	88	Feminino	703	4	sedentário	18.42653	1383.18	1532.127	1440.4	1475.411
423	50	Feminino	280	5	ativo	24.16716	1610.645	1703.811	1305.395	1370.953
424	28	Feminino	280	5	muito ativo	24.30462	1062.17	1256.743	1340.62	1399.557
425	83	Masculino	860	0	sedentário	19.37716	1734.665	1727.539	1413.81	1413.196
426	72	Feminino	860	0	ativo	20.56933	981.96	1138.381	797.475	935.8153
427	42	Feminino	253.75	8	ativo	25.71166	1411.94	1470.224	999.83	1116.706
428	26	Feminino	283.3333	11	insuf. ativo	24.08822	3368.82	3023.472	2220.6	2030.483
429	71	Feminino	6400	8	ativo	25	1047.15	1243.479	986.28	1117.804
430	27	Feminino	416.4286	5	ativo	27.37966	1570.93	1686.89	1042.175	1144.837
431	30	Feminino	100	6	ativo	24.60973	1703.71	1792.223	1904.435	1804.555
432	61	Feminino	150	0	muito ativo	27.05515	2615.77	2489.483	2192.28	2011.307
433	83	Masculino	207.5	2	ativo	23.30906	1021.59	1220.819	1181.7	1275.782
434	47	Masculino	900	11	muito ativo	20.0692	1562.44	1680.078	1334.28	1394.691
435	48	Feminino	900	11	ativo	28.125	2799.14	2622.324	2427.74	2168.926
436	66	Masculino	232.5	8	insuf. ativo	28.90625	1035.525	1180.349	611.9	781.5611
437	66	Masculino	415	4	sedentário	24.91349	1210.945	1373.89	1109.24	1211.701
438	62	Masculino	266.25	2	muito ativo	24.60973	1801.845	1716.6	1914.705	1687.509
439	45	Feminino			insuf. ativo	27.47563	1613.22	1721.332	1232.97	1316.137
440	70	Masculino	353.75	9	ativo	20.70313	670.88	897.1151	499.32	684.9017
441	51	Masculino	225	3	ativo	20.98399	1332.53	1489.622	1320.23	1383.887
442	54	Feminino	225	3	ativo	24.65591	1592.78	1704.76	1352.76	1408.859
443	20	Feminino	225	3	insuf. ativo	18.9069	2041.22	2059.072	1909.51	1816.213
444	25	Masculino	298.3333	5	muito ativo	21.45329	3607.465	3035.071	2898.725	2289.131
445	52	Masculino	307.5	5	muito ativo	19.4674	3572.37	3163.047	2503.95	2219.097
446	36	Masculino	466.6667	8	muito ativo	28.73467	3005.45	2769.517	1679.72	1652.262
447	34	Feminino	466.6667	8	insuf. ativo	19.10009	2199.86	2180.273	1673.94	1648.068
448	33	Feminino	333.3333	4	insuf. ativo	22.65625	922.08	1131.508	867.87	1018.411
449	33	Feminino	235.7143	7	muito ativo	22.77319	1617	1724.392	1446.51	1480.013
450	70	Feminino	100	0.5	insuf. ativo	39.11111	1163.755	1339.484	1139.97	1214.112

451	47	Feminino	233.3333	5	insuf. ativo	18.75	1978.18	2010.353	1661.83	1639.27
452	20	Masculino	233.3333	5	ativo	19.53125	1006.41	1207.309	636.69	814.2934
453	42	Feminino	766.6667	11	sedentário	26.5625	1198.125	1363.049	740.21	904.2361
454	34	Feminino	90	0	ativo	32.88889	1142.5	1327.065	850.82	1003.84
455	59	Masculino	260	8	ativo	27.82932	1619.08	1726.075	1373.51	1424.71
456	27	Masculino	260	8	sedentário	22.89308	1857.45	1916.107	1478.7	1504.178
457	54	Feminino	260	8	ativo	21.71925	1266.7	1433.878	980.15	1112.732
458	30	Feminino	260	8	ativo	16.79688	637.03	864.3851	636.5	814.1191
459	81	Feminino	375	4	insuf. ativo	22.95918	2999.64	2765.403	1978.96	1864.768
460	75	Feminino	683	9	sedentário	31.11111	938.725	1146.374	1223.185	1300.432
461	23	Feminino	500	12	insuf. ativo	24.96494	1589.32	1701.95	1196.41	1287.403
462	30	Feminino	500	12	ativo	26.70362	2044.86	2061.875	1945.15	1841.185
463	71	Feminino	1216.667	0.5	insuf. ativo	25.71166	1357.385	1487.364	1674.525	1642.854
464	55	Masculino	579	11	insuf. ativo	24.22145	2707.065	2546.019	3479.74	2550.223
465	81	Feminino	579	11	sedentário	33.29865	623.97	851.6743	555.01	738.225
466	79	Feminino	382.5	3	sedentário	21.36752	361.74	584.8606	631.28	809.3275
467	72	Masculino	292.8571	5	sedentário	24.60973	1076.99	1074.044	1323.255	1288.303
468	31	Masculino	292.8571	5	sedentário	25.71166	1318.31	1477.629	2054.65	1917.198
469	30	Masculino	292.8571	5	ativo	20.67901	1193.12	1370.869	1079.85	1194.313
470	63	Feminino	292.8571	5	sedentário	27.81588	1594.585	1703.468	1130.985	1233.865
471	41	Feminino	292.8571	5	insuf. ativo	32.38836	1152.03	1335.341	1535.95	1546.836
472	68	Feminino	866.6667	4	insuf. ativo	29.29688	908.285	1085.69	1174.205	1214.124
473	73	Feminino	363	4	sedentário	31.25	1511.64	1638.527	831.79	987.4941
474	38	Masculino	240	0.5	ativo	24.22145	2253.945	2076.971	1906.84	1623.188
475	38	Feminino	240	0.5	ativo	25.39063	788.625	992.7435	1048.515	1111.436
476	64	Feminino	669	8	ativo	22.40588	1650.25	1744.067	1205.255	1293.706
477	39	Feminino	283.3333	6	ativo	24.00549	435.01	661.9083	510	695.2275
478	62	Feminino	4400	13	muito ativo	22.30815	1215.865	1390.367	1921.61	1780.981
479	72	Feminino	583.3333	0	sedentário	46.74557	1012.67	1174.149	1127.785	1221.97
480	57	Masculino	560	6	ativo	26.57313	2074.8	2084.891	1106.89	1216.118
481	28	Masculino	560	6	sedentário	20.00657	2124.49	2122.934	964.95	1100.122
482	25	Masculino	560	6	ativo	24.72518	1933.41	1975.551	1086.62	1199.785
483	55	Feminino	560	6	insuf. ativo	22.93975	2863.05	2668.169	1792.27	1733.223
484	26	Feminino	560	6	ativo	16.79688	1926.3	1970.009	1342.13	1400.715
485	72	Feminino	776.6667	2	insuf. ativo	33.6593	923.615	1132.899	1041.185	1159.144
486	74	Masculino	411.6667	4	sedentário	22.40879	1750.81	1831.769	1213.8	1300.015
487	60	Feminino	411.6667	4	insuf. ativo	26.37024	1638.945	1707.752	1637.33	1619.361
488	61	Feminino		8	muito ativo	26.17188	943.52	1150.903	748.29	914.6838
489	79	Masculino	310	0	ativo	23.30668	2125.38	2123.613	1502.33	1521.834
490	78	Feminino	310	0	insuf. ativo	28.53745	2119.94	2119.458	1726.9	1686.361
491	53	Feminino		4	insuf. ativo	25.55884	1051.37	1247.209	816.14	973.9847

492	44	Masculino	482.5	4	muito ativo	19.88385	2309.21	2262.715	2656.6	2318.441
493	20	Masculino	482.5	4	sedentário	22.14533	4250.17	3615.689	1754.21	1705.992
494	40	Feminino	482.5	4	muito ativo	17.96875	2432.63	2354.755	1398	1443.342
495	44	Masculino	960	11	muito ativo	25.95156	4892.16	4029.584	5406.93	3925.168
496	55	Feminino	683.3333	4	muito ativo	29.04866	1052.605	1235.586	1046.125	1127.716
497	51	Feminino	27.5	0	ativo	23.53036	513.91	742.5694	425.09	611.7
498	21	Masculino	27.5	0	ativo	20.74507	1022.9	1221.984	603.75	783.9028
499	31	Masculino	800	2	muito ativo	23.62445	1452.72	1549.4	1426.26	1422.539
500	65	Masculino	316.6667	4	sedentário	26.57313	1037.5	1234.935	1330.06	1391.175
501	29	Feminino	212.5	4	ativo	28.90625	1770.59	1847.495	1146.3	1247.668
502	65	Masculino	420	4	sedentário	25.14861	2005.055	2010.282	1869.705	1786.978
503	61	Feminino	420	4	insuf. ativo	27.88762	1457.265	1593.714	1176.095	1269.386
504	21	Feminino	500	10	insuf. ativo	31.25	1433.905	1568.993	1501.365	1487.486
505	28	Masculino	500	10	insuf. ativo	25.71166	1622.545	1660.876	1254.09	1274.444
506	37	Masculino		7	ativo	23.66144	1424.04	1566.184	1681.87	1653.821
507	69	Feminino	600	0	insuf. ativo	19.56296	613.04	841	527.12	711.679
508	46	Feminino	1040	0	ativo	29.96878	591.14	819.5099	700.11	871.8049
509	25	Feminino	1040	0	ativo	20.13478	1016.5	1216.294	736.66	904.3943
510	84	Masculino	506	3	ativo	19.94321	1349.35	1438.542	925.84	1058.49
511	76	Feminino	506	3	insuf. ativo	28.06183	866.71	1081.007	912.165	1055.057
512	82	Feminino	737.3333	2	sedentário	19.14672	998.94	1200.483	1010.42	1128.711
513	74	Masculino	357.5	4	insuf. ativo	29.86055	773.29	994.3752	846.05	999.7512
514	78	Feminino	357.5	4	insuf. ativo	26.44898	660.055	847.7603	702.895	757.175
515	88	Feminino	1500	11	insuf. ativo	18.66667	1748.81	1830.18	1399.56	1444.526
516	53	Feminino	1750	13	ativo	21.04805	2245.22	2214.576	1507.08	1525.375
517	60	Masculino		13	muito ativo	24.90641	2045.99	2062.745	3051.81	2569.161
518	41	Masculino	6500	13	sedentário	25.45807	1817.47	1712.318	984.415	1004.381
519	90	Feminino	715	4	ativo	27.34375	862.76	1077.383	1305.33	1372.398
520	72	Feminino	415	4	ativo	19.43301	2468.56	2381.357	3002.64	2538.441
521	58	Feminino	4700	13	sedentário	18.42404	2007.85	2033.323	1602.41	1595.864
522	73	Masculino	366.6667	13	sedentário	19.59184	1718.9	1789.554	1751.405	1670.323
523	63	Feminino	416.6667	8	sedentário	24.12879	929.51	1138.239	874.05	1023.676
524	64	Feminino	3933.333	8	ativo	32.88242	1643.685	1740.895	1612.66	1601.233
525	37	Feminino	300	8	insuf. ativo	29.38476	716.885	924.4715	823.42	915.7507
526	34	Feminino	551.6667	11	ativo	16.609	2001.54	1910.058	2443.335	2160.2
527	43	Masculino	166.6667	6	sedentário	26.85185	1362.84	1514.148	1049.965	1161.027
528	50	Masculino	500	7	sedentário	26.23457	2850.54	2659.213	2505.98	2220.428
529	71	Feminino	900	2	sedentário	30.42185	617.54	837.1219	662.72	721.2036
530	66	Feminino	548.3333	3	insuf. ativo	22.65625	1220.61	1388.234	875.355	1008.59
531	57	Masculino	1728.333	2	ativo	19.1953	1411.925	1535.462	1358.91	1410.79
532	58	Feminino	1728.333	2	ativo	22.47659	1556.965	1673.78	1361	1414.872

533	43	Feminino	419	4	muito ativo	24.74094	1404.65	1550.048	1233.13	1316.262
534	71	Feminino			insuf. ativo	39.66942	2180.77	2165.79	1064.06	1181.519
535	51	Masculino		10	insuf. ativo	21.2031	2076.27	2086.02	1832.84	1762.091
536	88	Feminino		13	sedentário	24.97399	1131.34	1317.24	996.815	1121.468
537	63	Feminino		13	sedentário	25.72103	1050.85	1241.502	973.245	1100.896
538	60	Feminino		13	insuf. ativo	23.05176	942.97	1150.308	1164.555	1242.96
539	62	Masculino	1600	13	sedentário	31.40766	1338.935	1467.799	1134.525	1193.162
540	67	Masculino	498.3333	11	sedentário	26.54321	332.77	553.7544	747.09	913.6239
541	66	Feminino	498.3333	11	insuf. ativo	27.58732	710.845	920.3782	1010.75	1122.625
542	79	Masculino	963	8	ativo	20.0692	2546.505	2432.699	1910.87	1817.086
543	67	Feminino	963	8	ativo	19.10009	987.725	1176.795	934.5	1051.246
544	67	Masculino	521.6667	11	sedentário	28.3737	1344.665	1480.516	1016.51	1141.455
545	70	Feminino	521.6667	11	insuf. ativo	24.00549	815.685	1017.088	540.165	720.5574
546	35	Masculino		6	sedentário	28.01022	4517.29	3789.507	2958.45	2510.722
547	30	Feminino		6	sedentário	21.63115	1723.9	1786.737	1335.67	1346.881
548	73	Masculino	1150	11	insuf. ativo	27.77778	1190.99	1366.61	1250.675	1309.8
549	55	Masculino	2440	13	insuf. ativo	27.68166	1726.405	1810.87	1438.45	1468.868
550	24	Masculino	2440	13	sedentário	26.72993	1373.99	1524.439	906.62	1051.277
551	51	Feminino	2440	13	ativo	28.51563	683.915	903.4127	601.495	778.2223
552	85	Masculino	937.5	4	sedentário	29.01745	2379.425	2298.973	2528.04	2233.909
553	63	Masculino	4500	11	sedentário	26.09328	1950.05	1988.506	1638.27	1622.107
554	54	Feminino	1086.667	11	ativo	34.72223	1844.8	1882.503	1874.22	1787.938
555	80	Masculino		10	ativo	26.42357	1802.52	1872.799	1759.16	1709.542
556	79	Feminino		10	ativo	20.9042	1723.61	1810.089	1478.63	1504.126
557	57	Masculino	675	9	insuf. ativo	44.59877	909.76	1120.324	984.23	1116.109
558	79	Masculino	1137.333	2	sedentário	32.28306	2089.815	2079.555	2113.645	1937.48
559	80	Feminino	1137.333	2	sedentário	26.44628	1617.36	1677.803	1644.02	1602.59
560	72	Feminino	820	13	muito ativo	26.15933	1760.38	1821.199	1487.39	1384.636
561	57	Feminino		7	ativo	29.38468	2226.795	2192.667	1343.61	1388.369
562	21	Masculino	400	11	ativo	22.20599	1275.88	1441.686	854.79	1007.239
563	20	Feminino	400	11	muito ativo	18.67093	1257.915	1388.992	941.35	1046.962
564	35	Masculino	2000	11	insuf. ativo	24.22145	2348.925	2276.489	1519.12	1531.454
565	59	Masculino	1666.667	4	sedentário	21.45329	833.14	1050.091	866.34	1017.107
566	62	Masculino	2550	11	sedentário	31.09789	1350.54	1484.673	1184.385	1257.488
567	31	Feminino	200	4	ativo	20	1120.03	1307.497	1057.77	1176.409
568	62	Masculino	790	11	insuf. ativo	24.34176	1875.39	1926.827	1604.935	1551.118
569	59	Feminino	790	11	ativo	34.04903	1921.89	1928.702	1968.53	1857.17
570	28	Feminino	790	11	muito ativo	22.03172	2070.245	2074.336	1437.565	1473.231
571	86	Feminino	200	4	sedentário	29.13632	1294.09	1456.42	1229.395	1303.79
572	63	Masculino	475	0	insuf. ativo	28.40551	658.155	872.2985	786.545	937.126
573	43	Masculino	375	11	ativo	31.14187	1592.7	1704.088	1146.05	1245.298

574	36	Feminino	375	11	muito ativo	20.54569	1577.5	1692.342	623.87	802.5097
575	64	Feminino	228.75	4	insuf. ativo	15.58172	1011.42	1194.332	1025.185	1148.398
576	67	Feminino	235.8333	8	ativo	26.83518	1266.045	1410.066	1270.235	1345.088
577	70	Feminino	475	11	muito ativo	24.21875	1004.45	1205.562	672.99	847.3664
578	49	Feminino	500	8	muito ativo	29.35752	1430.14	1560.916	1175.075	1270.512
579	66	Feminino	277.5	1	insuf. ativo	25.39063	1435.625	1565.752	1168.71	1249.395
580	71	Feminino	415	4	insuf. ativo	31.25	1431.04	1567.863	1143.155	1238.088
581	34	Feminino	630	10	muito ativo	17.52781	1584.32	1695.953	1786.825	1716.698
582	41	Masculino	6000	12	ativo	27.43484	2943.26	2522.191	3020.135	2393.657
583	84	Feminino	866	11	insuf. ativo	23.33547	1503.19	1628.758	1246.3	1316.967
584	69	Feminino	1200	4	insuf. ativo	29.77778	1038.87	1236.15	1105.56	1215.049
585	61	Feminino	2350	13	insuf. ativo	36.73095	1182.98	1361.952	1485.275	1502.787
586	74	Feminino	775	11	ativo	26.7094	1191.47	1292.197	1415.055	1417.451
587	47	Masculino	1251.6	11	ativo	30.64453	2343.05	2288.055	2032.59	1901.969
588	48	Feminino	1251.6	11	ativo	20.96436	983.6	1186.933	551.76	735.1475
589	25	Feminino	1251.6	11	ativo	19.53125	2508.5	2410.829	2454.39	2186.515
590	24	Feminino	1251.6	11	muito ativo	19.47341	1968.98	2003.215	1700.45	1667.274
591	69	Masculino	1000	11	muito ativo	25.71166	1342.94	1387.011	960.745	1030.098
592	64	Feminino	1000	11	insuf. ativo	29.29688	870.1851	1044.538	948.025	1054.064
593	33	Masculino	866.6667	11	ativo	21.46194	1904.08	1952.169	1678.64	1622.758
594	42	Feminino		11	insuf. ativo	20.61313	1232.205	1359.58	995.915	1098.876
595	47	Masculino	1666.667	11	sedentário	28.57796	1338.705	1494.287	1126.6	1231.637
596	49	Feminino	1666.667	11	muito ativo	23.33547	2666.59	2526.498	2306.46	2088.249
597	80	Feminino	2200	4	sedentário	23.42209	1805.63	1875.258	1148.21	1249.19
598	83	Masculino	448.5714	0.5	sedentário	23.79536	1504.81	1632.918	1355.7	1411.108
599	78	Feminino	448.5714	0.5	insuf. ativo	24.4646	1651.91	1752.581	1489.44	1512.212
600	69	Feminino	823.5	1	muito ativo	25.23634	1873.93	1929.047	1709.64	1673.914
601	45	Feminino	726.6667	11	ativo	20.56881	1186.645	1361.329	1427.515	1462.974
602	20	Masculino	726.6667	11	sedentário	20.04745	2269.18	2232.635	1530.12	1542.511
603	25	Feminino	726.6667	11	sedentário	37.34187	1482.53	1614.586	1058.64	1177.116
604	62	Masculino	666.25	4	ativo	24.22145	2338.84	2284.907	1485.86	1509.536
605	83	Masculino	666.25	4	sedentário	20.20202	1094.73	1285.37	646.95	823.6846
606	47	Masculino	575	11	ativo	34.74891	1664.22	1760.964	1043.78	1165.007
607	39	Feminino	575	11	sedentário	27.04164	1968.71	1999.604	1645.78	1620.12
608	58	Feminino	300	11	insuf. ativo	24.80159	998.13	1182.58	720.385	885.9934
609	25	Masculino	300	11	sedentário	22.69402	1026.85	1225.492	530.7	715.1039
610	23	Feminino	900	11	muito ativo	19.37716	1102.09	1291.818	854.27	1006.794
611	31	Feminino	900	11	ativo	20.98399	1178.86	1358.568	902.75	1048.01
612	22	Feminino		12	ativo	25.96953	951.3	1157.92	710.93	881.4931
613	66	Masculino	790	11	insuf. ativo	22.23099	2167.62	2142.92	1896.38	1784.916
614	64	Feminino	203	4	ativo	25.28257	1297.91	1460.378	719.51	889.1511

615	51	Feminino	1000	12	muito ativo	24.14152	1840.965	1830.977	1585.11	1543.714
616	26	Masculino	1000	12	muito ativo	18.937	3298.99	2895.093	3784.88	2838.357
617	46	Masculino	790	10	ativo	33.13976	2720.57	2565.646	2371.16	2131.418
618	48	Feminino	790	10	muito ativo	23.30668	1098.88	1289.007	1091.57	1203.78
619	66	Masculino	2000	11	muito ativo	27.33564	2221.6	2181.389	2002.735	1872.74
620	67	Feminino	1033.333	13	sedentário	33.26707	1146.735	1314.599	1356.58	1399.462
621	46	Masculino	2400	13	ativo	28.39506	2789.48	2607.128	1766.635	1712.608
622	46	Feminino	2400	13	insuf. ativo	31.64432	1331.8	1485.86	1078.98	1193.488
623	20	Feminino	2400	13	insuf. ativo	28.73469	1373.42	1509.455	1226.43	1304.963
624	42	Feminino	1200	11	muito ativo	21.09619	1139.84	1324.753	1317.91	1382.1
625	41	Masculino	825	4	sedentário	33.20313	856.74	1071.851	945.52	1083.932
626	38	Feminino	825	4	sedentário	19.37716	1203.53	1370.193	890.475	1035.324
627	69	Masculino	1080.667	4	muito ativo	32.17993	1645.45	1747.374	1555.7	1561.46
628	63	Feminino	1080.667	4	sedentário	26.29758	1602.25	1712.443	1579.99	1579.382
629	44	Masculino		8	muito ativo	24.09297	1779.055	1853.016	1118.45	1223.905
630	38	Feminino		8	ativo	28.04038	2071.75	2079.682	1826.42	1741.49
631	68	Masculino	1175	2	insuf. ativo	24.22145	1800.555	1865.686	1754.955	1681.186
632	63	Feminino	1175	2	sedentário	25.53671	1616.635	1709.599	1783.775	1703.3
633	35	Masculino	666.6667	13	ativo	25.7122	1010.95	1211.354	1239.96	1321.606
634	23	Masculino	350		insuf. ativo	23.88844	2316.05	2267.844	1579.32	1578.889
635	61	Masculino	1150	4	sedentário	27.54821	1586.24	1699.448	1319.32	1383.186
636	35	Feminino	1500	13	sedentário	21.45329	1490.32	1621.002	1149.32	1250.074
637	24	Feminino	750	11	insuf. ativo	20.17325	1731.245	1816.01	1300.105	1366.127
638	65	Masculino	1505	13	sedentário	34.06361	2091.635	2096.649	1378.765	1427.347
639	64	Feminino	1505	13	sedentário	26.67276	1795.165	1864.311	1182.005	1274.527
640	61	Feminino	936.25	8	ativo	31.11111	892.66	1104.751	933.5	1073.877
641	70	Masculino	378.75	4	ativo	27.54821	1498.24	1541.773	1235.46	1299.11
642	65	Feminino	378.75	4	sedentário	40.23438	1300.93	1419.633	1180.41	1274.761
643	37	Feminino	925	13	muito ativo	23.06805	2831.385	2625.135	2630.215	2301.337
644	60	Masculino	550	1	muito ativo	20.2449	1518.97	1644.54	1104.68	1214.341
645	65	Feminino	1317	0.5	insuf. ativo	24.03461	911.305	1121.721	895.27	1036.924
646	28	Masculino	720	10	sedentário	27.68166	2213.09	2187.749	1806.68	1739.467
647	28	Feminino	720	10	muito ativo	32.8473	1854.135	1913.448	1452.26	1480.625
648	31	Masculino	240	9	muito ativo	22.0384	3785.855	3303.898	2316.52	2086.885
649	35	Feminino	240	9	insuf. ativo	20.81166	2049.965	2065.777	1545.295	1551.089
650	65	Masculino	387.5	5	ativo	32	1576.6	1691.61	1368.93	1421.216
651	60	Feminino	387.5	5	ativo	27.07205	1256.87	1425.504	1156.52	1255.806
652	21	Feminino	387.5	5	ativo	19.60519	1373.46	1523.995	644.64	821.5732
653	21	Masculino	380.5	11	insuf. ativo	22.83951	1514.42	1640.808	1068.64	1185.235
654	60	Feminino	600	5	ativo	25.625	1458.74	1594.948	975.78	1109.111
655	83	Masculino	348.75	0	sedentário	21.09375	483.005	707.8363	378.895	559.3731

656	40	Masculino	620	10	muito ativo	28.04967	1841.905	1900.974	1745.07	1662.999
657	37	Feminino	620	10	ativo	24	1667.27	1761.644	1931.625	1777.353
658	76	Feminino	415	1	muito ativo	26.5625	943.57	1039.073	761.155	906.8516
659	68	Feminino	1600	4	sedentário	27.73909	1255.485	1423.049	1168.445	1246.202
660	92	Masculino	557.5	4	sedentário	21.875	1962.47	1998.16	1628.86	1615.234
661	81	Feminino	557.5	4	insuf. ativo	22.89282	1901.83	1950.899	1006.23	1134.259
662	36	Feminino	738.3333	5	insuf. ativo	23.66144	1272.91	1439.161	1291.16	1361.443
663	83	Feminino	440	0.5	ativo	26.84067	1793.75	1760.736	1656.9	1438.126
664	64	Feminino	380	4	insuf. ativo	27.05515	991.61	1145.92	1524.325	1528.956
665	69	Masculino	342	4	muito ativo	31.1993	1218.29	1392.509	1180.05	1274.476
666	68	Feminino	342	4	muito ativo	28.87544	964.32	1169.638	919.05	1061.747
667	76	Masculino	900	11	sedentário	23.5102	1961.43	1997.352	2481.34	2204.253
668	47	Masculino	302.1429	7	ativo	27.88519	1248.06	1403.584	1482.235	1504.053
669	46	Feminino	302.1429	7	insuf. ativo	24.21875	638.6	865.6987	542.08	724.7711
670	21	Feminino	302.1429	7	ativo	21.22789	1369.91	1521.022	1168.01	1264.934
671	68	Masculino	266.6667	8	sedentário	29.13632	1856.46	1915.329	2327.24	2102.147
672	21	Feminino	1040.8	12	sedentário	18.71095	1763.87	1842.158	1330.03	1391.426
673	55	Feminino	300	1	ativo	41.55125	921.17	1130.683	513.5	698.6008
674	50	Masculino	120	2	ativo	26.53376	679.36	905.2673	519.71	704.5733
675	41	Feminino	120	2	sedentário	27.43484	822.24	1040.002	1056.44	1175.328
676	55	Masculino	1000	4	ativo	27.88519	1191.915	1122.306	1508.105	1275.504
677	65	Feminino	1700	4	insuf. ativo	26.22269	1468.32	1591.553	1318.635	1380.95
678	82	Masculino	450	12	sedentário	20.95727	1295.66	1458.472	1288.52	1359.398
679	64	Feminino	1265	4	ativo	25.28257	1149.59	1333.224	1905.7	1813.536
680	51	Masculino		13	ativo	42.44898	1518.565	1597.416	1555.765	1420.17
681	46	Feminino	291.6667	5	ativo	23.30906	1027.765	1223.8	818.615	970.3436
682	46	Masculino	291.6667	5	ativo	27.54821	1782.295	1819.453	1133.925	1214.202
683	75	Feminino	978.5714	10	sedentário	18.02596	1233.375	1404.212	1196.05	1283.197
684	53	Masculino	412.5	12	sedentário	26.54321	2756.73	2591.775	1965.34	1855.28
685	21	Masculino	412.5	12	muito ativo	18.7883	3710.52	3256.764	1938.89	1836.807
686	47	Feminino	412.5	12	ativo	19.59184	1999.08	2026.541	1321.09	1384.549
687	60	Feminino	305	11	sedentário	23.33768	934.03	1134.398	1468.255	1426.087
688	61	Feminino	1370	7	ativo	30.66667	1696.99	1788.748	1739.54	1693.152
689	71	Masculino	2266.667	11	sedentário	37.02422	2064.63	2077.081	2050.9	1914.612
690	76	Feminino	625	12	ativo	24.60973	1117.62	1268.636	959.73	1000.679
691	60	Feminino	625	12	ativo	29.04866	1179.545	1311.624	1016.425	1124.348
692	62	Feminino	800	4	sedentário	29.40228	789.27	1009.329	917.28	1060.258
693	78	Feminino	915	4	sedentário	28.80441	1090.16	1281.362	1473.5	1500.283
694	80	Masculino	615	0.5	sedentário	24.22145	1911.035	1932.661	1925.06	1824.883
695	80	Feminino	615	0.5	insuf. ativo	31.91111	2671.495	2414.027	2567.98	2259.745
696	62	Masculino	1250	8	ativo	26.26494	2832.57	2646.333	2380.96	2137.931

697	72	Feminino	650	5	sedentário	18.25632	1400.25	1546.38	1277.78	1351.07
698	84	Masculino	700	1	ativo	21.48438	2177.16	2119.694	2042.995	1890.451
699	76	Feminino		3	ativo	30.8642	1627.79	1692.748	1987.15	1713.13
700	78	Feminino	415	0.5	insuf. ativo	25.78125	1289.045	1418.007	1152.79	1233.546
701	91	Feminino	174.1429	4	sedentário	24.22145	986.01	1189.09	861.47	1012.95
702	42	Feminino	372.5	11	insuf. ativo	23.4375	906.14	1117.032	810.92	969.4651
703	38	Feminino	325	3	muito ativo	22.26563	2469.33	2381.926	1413.72	1455.26
704	67	Masculino	1050	4	ativo	28.65014	716.77	940.6956	940.305	1063.938
705	63	Feminino	1050	4	ativo	32.38836	954.225	1137.366	1359.425	1363.782
706	60	Masculino	800	4	ativo	35.37981	1442.75	1581.711	950.84	1088.373
707	49	Masculino	600	2	sedentário	33.05785	1757.355	1804.759	1687.75	1651.626
708	34	Feminino	250	11	ativo	28.82753	1454.41	1591.366	1052.66	1172.253
709	70	Feminino	150	2	muito ativo	23.53036	1501.07	1629.845	1288.11	1359.08
710	49	Feminino	120	5	ativo	25.25951	1455.33	1592.127	1012.67	1139.554
711	84	Feminino	235.8333	5	sedentário	18.42653	1889.45	1941.212	1722.82	1683.421
712	20	Feminino	241.6667	1	sedentário	20.93664	1925.55	1955.422	955.115	1091.739
713	60	Feminino	100	0	sedentário	24.97399	2433.56	2355.445	2356.9	2121.93
714	66	Feminino	1750	4	ativo	22.20633	1151.66	1334.901	987.05	1117.733
715	52	Feminino		4	sedentário	27.88762	685.945	899.8911	805.27	959.6179
716	61	Feminino	83	3	insuf. ativo	28.93518	1219.28	1365.938	1108.19	1212.118
717	26	Masculino	645	0	ativo	22.53061	1517.47	1634.885	1084.805	1192.162
718	73	Masculino	1365	12	sedentário	25.71166	1912.78	1956.24	1336.035	1395.99
719	33	Feminino	42.85714	1	sedentário	31.55556	950.22	1156.947	1106.54	1215.837
720	78	Feminino	415	0	insuf. ativo	25.39063	457.5	673.6194	436.925	623.2593
721	60	Masculino	750	9	insuf. ativo	25.78125	1824.39	1890.075	1506.14	1524.675
722	55	Feminino	60	5	ativo	22.20633	953.315	1159.69	1006.56	1133.486
723	35	Feminino	333.3333	8	ativo	22.83288	2867.12	2671.081	2272.24	2065.292
724	72	Masculino	330.625	4	sedentário	22.14533	2666.59	2479.395	1542.425	1547.854
725	48	Masculino	300	11	ativo	23.597	2924.01	2711.688	1883.04	1797.591
726	80	Feminino	415	4	ativo	31.95763	1321.24	1480.102	1223.81	1308.957
727	33	Masculino	525	5	sedentário	22.85714	1448.1	1586.143	1057.93	1176.539
728	50	Feminino	700	3	ativo	25.77778	1405.23	1550.531	1237.5	1319.682
729	89	Feminino	439.3333	5	sedentário	29.66655	1190.295	1358.588	1458.11	1487.223
730	71	Feminino	665	2	insuf. ativo	24.03461	872.53	1086.345	924.08	1065.975
731	57	Masculino	250	3	sedentário	24.97704	1031.73	1229.207	1209.345	1274.59
732	49	Masculino	250	3	ativo	22.65625	1376.515	1525.759	1277.305	1340.887
733	59	Feminino	250	3	ativo	26.22269	935.73	1143.866	904.12	1049.167
734	72	Feminino	315	3	sedentário	25.77778	1275.48	1416.155	1370.765	1408.713
735	47	Masculino	284	4	ativo	22.03857	2292.88	2250.458	1543.66	1552.551
736	38	Feminino	284	4	insuf. ativo	37.5	1652.97	1753.435	1574.07	1575.021
737	36	Feminino	200	3	ativo	28.57796	780.475	995.4502	528.31	698.5198

738	64	Feminino	350	11	muito ativo	27.99302	1328.9	1486.31	1067.71	1180.802
739	64	Feminino	84.54546	0	muito ativo	25.78125	1129	1315.149	872.39	1019.363
740	32	Feminino	103.3333	7	muito ativo	23.97531	831	1027.33	626.7	797.478
741	70	Masculino		9	ativo	25.68956	1010.7	1211.131	1902.16	1811.049
742	67	Feminino		9	ativo	32.04615	1318.91	1478.136	2195.09	2013.212
743	62	Feminino	205	4	insuf. ativo	24.09297	1575.975	1624.654	1930.72	1817.351
744	45	Feminino		11	insuf. ativo	24.21875	759.395	970.3569	1043.805	1152.806
745	71	Feminino	207.5	4	ativo	29.67494	1123.98	1310.943	912.09	1055.889
746	62	Masculino	250.5	11	ativo	32.98792	1756.46	1835.87	2032.75	1892.391
747	33	Feminino		13	insuf. ativo	26.03749	1668.82	1766.19	1751.16	1703.803
748	67	Masculino	567.5	4	sedentário	23.32415	2594.575	2397.566	2147.025	1945.077
749	61	Feminino	567.5	4	insuf. ativo	22.71897	2262.22	2220.569	1651.09	1622.564
750	55	Masculino	783.3333	4	sedentário	21.22449	2167.58	2149.913	2007.425	1883.901
751	26	Masculino	783.3333	4	muito ativo	31.97502	3536.01	3138.248	3553.39	2875.717
752	73	Feminino	250	2	insuf. ativo	29.13632	872.14	1085.988	712.1	882.5386
753	39	Feminino		8	ativo	27.33564	1827.76	1820.338	1847	1746.042
754	77	Masculino	707.5	11	sedentário	30.854	1706.02	1796.028	1084.08	1197.733
755	79	Feminino	707.5	11	insuf. ativo	35.62902	1293.93	1424.493	1319.825	1379.905
756	31	Masculino		11	sedentário	24.18705	3264.49	2951.209	2711.9	2354.068
757	33	Feminino		11	insuf. ativo	19.83471	1530.855	1650.617	1007.275	1129.125
758	72	Feminino		4	sedentário	33.41241	1467.37	1602.079	935.18	1075.284
759	76	Masculino	957.5	4	sedentário	26.82742	1348.28	1502.874	1345.21	1403.076
760	77	Feminino	957.5	4	sedentário	24.32501	1356.345	1495.891	1384.69	1433.12
761	64	Masculino	375	11	ativo	23.12467	2299.82	2255.67	1740.67	1696.269
762	75	Feminino	103.75	11	sedentário	28.39872	1224.865	1336.135	1240.495	1209.308
763	59	Feminino	103.75	1	sedentário	22.86254	1689.99	1764.595	1355.075	1410.63
764	72	Feminino		8	ativo	30.44983	930.635	1135.698	923.82	1063.365
765	81	Masculino	92.22222	0	sedentário	24.38272	1334.33	1491.138	1135.87	1239.344
766	85	Feminino	92.22222	0	insuf. ativo	25.56611	838.01	1054.591	745.87	912.5459
767	37	Masculino	960	9	ativo	27.99474	3205.62	2910.207	2394.11	2146.659
768	36	Feminino	960	9	ativo	27.1809	1031.83	1229.911	592.33	773.2783
769	20	Feminino	960	9	insuf. ativo	24.65483	350.79	573.1486	432.96	619.5869
770	63	Feminino	220	4	sedentário	33.29865	879.95	1093.139	885.87	1033.721
771	78	Masculino		10	sedentário	19.57992	1144.16	1221.94	1840.03	1605.23
772	75	Feminino		10	insuf. ativo	35.13355	1276.635	1430.835	1231.435	1295.577
773	73	Masculino	285	8	ativo	29.05329	960.58	1166.275	2128.1	1967.614
774	72	Feminino	285	8	insuf. ativo	26.76978	684.17	909.8831	981.59	1113.924
775	56	Masculino	166.6667	8	muito ativo	24.6181	3085.64	2826.12	2056.8	1918.681
776	30	Masculino	166.6667	8	ativo	19.88385	3863.26	3359.475	4724.32	3551.534
777	61	Feminino	207.5	1	insuf. ativo	26.44628	1464.645	1580.47	1223.895	1308.819
778	64	Masculino	207.5	1	ativo	20.57143	970.98	1173.599	789.46	949.9108

779	89	Masculino		1	sedentário	18.51852	1308.92	1469.695	1022.05	1147.251
780	53	Feminino	300	11	ativo	24.91349	1608.15	1716.719	1014.495	1141.036
781	52	Feminino	850	4	insuf. ativo	27.34453	1443.79	1582.573	978.21	1111.125
782	62	Masculino		8	sedentário	29.62963	2383.6	2318.316	1852.53	1776.044
783	47	Masculino	800	10	muito ativo	21.45329	1799.75	1867.504	1341.645	1386.088
784	72	Feminino	69.16666	4	ativo	22.77319	1091.14	1282.222	1028.88	1152.845
785	78	Masculino	415	2	ativo	21.22449	1453.96	1590.994	1565.44	1568.655
786	68	Feminino	415	2	ativo	25.33333	917.91	1127.726	1092.53	1204.555
787	39	Feminino	570	11	ativo	27.23922	2034.58	2053.956	1978.73	1864.608
788	71	Feminino	276.6667	3	ativo	27.23922	1716.745	1792.888	1358.585	1412.1
789	44	Feminino		4	sedentário	25.0995	997.36	1199.236	1111	1219.421
790	72	Masculino		4	insuf. ativo	25.40282	1978.935	2010.129	2453.26	2171.938
791	70	Feminino	415	3	insuf. ativo	32.04995	632.18	795.5436	716.365	878.7465
792	80	Feminino	103.75	6	sedentário	20.82999	2463.385	2174.615	1740.34	1632.292
793	29	Feminino		8	insuf. ativo	26.63892	1858.845	1843.237	1164.44	1254.588
794	68	Masculino		1	sedentário	26.44898	1240.77	1411.76	1235.02	1317.742
795	64	Feminino	83	11	sedentário	21.77384	2351.245	2274.917	2102.93	1935.779
796	63	Masculino	207.5	11	ativo	25.46939	1101.235	1265.588	898.73	1035.255
797	82	Feminino	207.5	11	insuf. ativo	26.48554	1014.525	1196.361	1035.095	1139.065
798	78	Feminino	650	3	insuf. ativo	31.25	2182.18	2035.173	1701.03	1444.346
799	45	Masculino		11	muito ativo	18.93878	2219.45	2166.843	2191.745	1990.259
800	28	Masculino	485	4	muito ativo	29.62963	1105.34	1294.662	1164.23	1261.933
801	70	Feminino	415	0.5	sedentário	22.06035	1511.575	1635.918	1276.435	1318.792
802	49	Masculino	324	3	muito ativo	20.43817	1061.27	1246.682	1109.135	1217.364
803	44	Feminino	324	3	ativo	29.38476	1542.885	1655.885	1323.905	1381.628
804	23	Feminino	324	3	ativo	26.49151	1067.275	1257.813	642.325	811.5958
805	32	Feminino	1000	11	sedentário	20.70313	1351.24	1504.461	1133.3	1237.164
806	87	Masculino	415	4	sedentário	20.44444	978.85	1182.678	931.88	1072.519
807	86	Feminino	415	4	sedentário	29.33333	699.65	924.6981	574.8	756.8777
808	63	Masculino	165	4	ativo	29.35236	2023.115	2038.898	1663.315	1596.223
809	62	Feminino	165	4	insuf. ativo	23.52941	1743.575	1820.967	1500.85	1459.022
810	55	Feminino	3533.333	13	sedentário	29.05329	1185.71	1336.374	1460.72	1428.431
811	24	Feminino	3533.333	13	sedentário	21.19274	1087.88	1279.362	798.04	958.2834
812	74	Masculino	415	4	sedentário	19.03114	1178.96	1357.908	1005.78	1123.969
813	72	Feminino	415	4	insuf. ativo	35.37981	1274.725	1427.008	1311.74	1363.368
814	23	Masculino	525	8	sedentário	20.2449	2481.35	2374.925	1894.38	1800.174
815	36	Feminino	600	8	insuf. ativo	25.25951	1692.565	1741.693	1542.105	1547.304
816	76	Feminino	1900	11	insuf. ativo	36.19875	1133.35	1299.744	804.89	932.9737
817	75	Masculino	207.5	13	ativo	25.20776	1704.34	1794.683	1670.86	1645.832
818	74	Feminino	207.5	13	sedentário	35.75595	826.42	1043.874	809.9	968.5811
819	49	Masculino	2125	13	sedentário	29.03674	1956.695	1941.419	1946.06	1800.653

820	47	Feminino	2125	13	ativo	26.986	1806.515	1867.215	1642.995	1619.101
821	20	Feminino	2125	13	ativo	25.23634	1701.125	1792.108	1362.705	1415.106
822	72	Masculino	300	4	ativo	26.06168	1964.48	1999.612	1479.63	1497.375
823	70	Feminino	300	4	ativo	22.67995	1814.075	1878.284	1716.295	1675.963
824	67	Feminino	975.3333	11	ativo	27.05515	1484.76	1615.918	1359.05	1407.103
825	60	Masculino	975.3333	11	ativo	28.34467	2191.875	2172.987	2745.13	2314.758
826	90	Feminino	975.3333	11	sedentário	22.31328	1360.615	1504.024	1277.35	1350.39
827	75	Feminino		4	sedentário	19.59646	1132.95	1318.758	1207.93	1296.48
828	68	Feminino	425	4	muito ativo	28.67263	2249.895	2207.302	1825.7	1670.691
829	52	Masculino		8	ativo	31.14187	1553.44	1626.439	1402.115	1301.012
830	67	Feminino	743	4	ativo	29.77778	1385.045	1469.246	1381.28	1359.249
831	30	Feminino	3000	13	muito ativo	25.77778	1095.22	1285.8	1407	1450.169
832	70	Feminino	2200	13	muito ativo	23.23346	1137.41	1322.64	1101.53	1211.806
833	40	Masculino	686	11	muito ativo	38.73967	1480.165	1608.191	1886.245	1793.318
834	65	Feminino	207.5	4	muito ativo	23.11111	1752.18	1830.636	1400.27	1432.081
835	69	Masculino	2625	11	ativo	25.38426	1690.55	1759.73	1781.935	1659.253
836	74	Feminino	1600	1	ativo	31.18459	2217.7	2193.782	2083.42	1936.999
837	85	Masculino	1200	11	muito ativo	25.63692	2189.135	2148.258	1743.66	1696.823
838	68	Masculino	530	4	insuf. ativo	27.9431	1459.7	1595.741	1222.41	1307.859
839	41	Masculino	400	5	muito ativo	22.49135	3147.15	2869.318	2972.83	2519.754
840	20	Masculino	400	5	insuf. ativo	23.14815	3469.47	2947.16	2032.635	1846.126
841	44	Feminino	400	5	insuf. ativo	23.73866	1110.965	1273.319	976.845	1037.521
842	66	Feminino	1855	4	insuf. ativo	34.375	1155.345	1338.209	1228.12	1311.98
843	62	Feminino	616.6667	4	insuf. ativo	27.34375	1517.83	1643.605	1185.59	1278.858
844	30	Masculino	1395	12	muito ativo	21.87755	3989.49	3443.673	4371.98	3353.261
845	29	Masculino	1395	12	muito ativo	20.04745	2880.03	2680.311	2518.45	2228.598
846	62	Feminino		12	ativo	33.30499	1331.24	1488.535	1689.03	1659.01
847	78	Masculino	489.895	11	insuf. ativo	27.99036	1413.645	1526.81	1743.81	1642.534
848	72	Feminino	489.895	11	ativo	26.03749	920.07	1129.412	1176.635	1227.712
849	58	Feminino	741.25	8	insuf. ativo	29.47584	2729.83	2572.344	1909.52	1816.22
850	32	Masculino	741.25	8	muito ativo	36.6782	3718.8	3262.356	2680.52	2333.874
851	32	Feminino	741.25	8	ativo	22.18935	1322.19	1480.904	1016.2	1142.453
852	69	Masculino	900	0	ativo	28.125	1728.83	1814.255	1318.99	1382.932
853	33	Masculino	201.6	6	insuf. ativo	24.4418	816.45	1034.633	430.74	617.3653
854	29	Feminino	201.6	6	muito ativo	17.99816	724.05	845.1832	974.395	914.6487
855	72	Feminino	415	4	insuf. ativo	27.58732	2305.71	2260.09	2281	2071.177
856	66	Masculino	207.5	4	sedentário	27.34375	1605.735	1657.852	1730.785	1650.154
857	72	Feminino	207.5	4	sedentário	31.14187	758.86	973.2784	1155.74	1248.316
858	59	Feminino	638.3333	4	insuf. ativo	26.5625	1281.845	1445.888	1226.945	1305.654
859	76	Feminino	138.3333	5	sedentário	24.88889	1270.5	1437.112	1014.31	1140.901
860	83	Feminino		4	sedentário	27.34375	872.785	1068.022	988.58	1103.029

861	75	Masculino	696	13	ativo	24.22145	1518.73	1643.335	1854.965	1734.911
862	65	Feminino	696	13	ativo	27.68878	1067.865	1261.323	1703.835	1628.838
863	35	Feminino		11	ativo	23.1405	2127.96	2125.583	1420.36	1460.284
864	61	Feminino	103.75	3	sedentário	27.60945	1946.48	1985.729	1464.69	1493.677
865	75	Masculino	2415	0.5	ativo	19.94321	1720.445	1793.499	1580.57	1537.895
866	22	Masculino	762	12	insuf. ativo	23.5102	2828.44	2643.37	1588.57	1585.697
867	28	Feminino	762	12	insuf. ativo	21.48438	2582.83	2221.139	1170.565	1169.017
868	70	Masculino	303.75	3	muito ativo	20.65754	1217.8	1392.089	751.79	917.7731
869	21	Masculino	303.75	3	sedentário	24.56747	2452.92	2369.788	2700.64	2346.829
870	61	Feminino	303.75	3	sedentário	28.34467	1039.8	1236.974	1032.49	1155.798
871	61	Masculino		12	muito ativo	25.68956	1549.31	1669.366	1689.9	1659.64
872	60	Feminino		12	muito ativo	27.91552	2042.12	2059.765	1881.89	1796.78
873	39	Masculino	675	12	ativo	30.08383	1254.3	1423.313	941.72	1080.757
874	41	Feminino	675	12	insuf. ativo	21.77384	1239.715	1410.847	1105.825	1184.734
875	72	Feminino	783.3333	4	ativo	33.05785	933.3	1134.314	553.515	705.2601
876	67	Feminino	1122.5	4	ativo	28.39872	3053.15	2803.226	2076.06	1931.94
877	78	Feminino	207.5	4	sedentário	35.59986	1550.93	1661.639	1517.205	1532.776
878	72	Masculino	560.6667	4	ativo	25.21736	672.5	898.674	684.01	857.324
879	65	Masculino	560.6667	4	sedentário	24.03461	489.94	718.2973	649.96	826.4332
880	62	Feminino	160		insuf. ativo	30.0621	1015.345	1193.666	805.85	957.6816
881	36	Masculino	500	13	ativo	22.30935	1726.065	1764.666	1824	1656.067
882	24	Feminino	500	13	insuf. ativo	24.38653	2251.28	2219.148	1631.35	1617.054
883	85	Masculino	229	0.5	ativo	24.21875	1444.42	1484.427	1325.685	1317.179
884	82	Feminino	229	0.5	insuf. ativo	17.84652	1346.995	1498.635	1427.625	1464.407
885	56	Feminino	216.6667	8	insuf. ativo	25.91068	1486.01	1586.242	1167.36	1264.418
886	45	Masculino	625	11	ativo	24.60973	1895.49	1945.94	1482.86	1507.292
887	39	Feminino	625	11	muito ativo	27.34375	1805.6	1875.234	1118.4	1225.36
888	45	Feminino	435.8333	11	ativo	29.64268	3204.57	2909.474	1466.27	1494.863
889	49	Feminino	288.3333	6	insuf. ativo	21.77778	1534.26	1657.064	1269.11	1344.335
890	37	Masculino	795.25	11	muito ativo	29.06977	1050.84	1246.741	1148.5	1249.421
891	34	Feminino	795.25	11	ativo	26.25958	1192.86	1369.513	1011.575	1137.898
892	73	Feminino	755	4	ativo	18.75	1681.83	1776.641	1035.61	1158.349
893	35	Feminino	755	4	ativo	18.36547	1707.28	1797.036	1004.89	1133.156
894	56	Masculino	681.6667	4	muito ativo	26.81222	1722.79	1629.845	1338.67	1271.781
895	54	Feminino	681.6667	4	sedentário	30.66667	1549.46	1641.746	1025.805	1143.018
896	61	Masculino	145	0	ativo	23.11111	565.56	794.2304	424.53	611.1375
897	30	Masculino	433.3333	9	muito ativo	29.38775	2178.41	2163.998	2048.15	1912.715
898	53	Feminino	580	8	insuf. ativo	28.99931	1338.9	1471.222	1151.285	1231.737
899	24	Feminino	580	8	ativo	22.60027	1911.845	1934.138	1748.505	1695.677
900	83	Feminino	615	5	sedentário	38.26531	1133.74	1319.446	958.76	1094.973
901	69	Masculino	316.25	8	ativo	25.65437	1361.48	1505.678	1139.49	1241.811

902	73	Feminino	316.25	8	insuf. ativo	24.24242	1199.395	1340.651	933.44	1049.355
903	26	Masculino	832	8	ativo	23.45092	1233.975	1401.004	979.425	1103.502
904	24	Masculino	832	8	ativo	21.67126	1415.52	1559.099	942.58	1081.476
905	22	Masculino	832	8	muito ativo	21.30682	1652.35	1752.936	1369.49	1421.644
906	22	Masculino	832	8	ativo	24.60973	1624.635	1694.898	1822.79	1641.16
907	85	Feminino		4	sedentário	25.31545	1627.65	1733.005	1231.86	1315.267
908	41	Feminino	725	8	insuf. ativo	25.91068	2602.395	2383.676	2418.53	2084.978
909	73	Feminino	1229	10	insuf. ativo	18.92301	1251.33	1420.779	950.48	1088.073
910	78	Masculino		13	muito ativo	25.46939	1240.9	1408.124	1244.64	1323.979
911	78	Feminino		13	insuf. ativo	20.95661	1270.45	1435.086	1270.14	1342.727
912	29	Feminino	250	7	insuf. ativo	20.96436	1547.94	1668.247	1243.43	1324.319
913	53	Masculino	400	5	muito ativo	36.32813	2110.985	2045.943	1506.875	1509.1
914	41	Feminino	400	5	muito ativo	18.35938	608.47	808.8169	591.435	764.2544
915	77	Masculino	1080	6	ativo	28.02768	2261.01	2224.047	1340.375	1399.091
916	51	Masculino	1240	8	muito ativo	29.06977	2417.6	2343.602	2620.38	2295.003
917	25	Masculino	1240	8	sedentário	23.58833	3445.76	3076.446	4756.32	3569.349
918	23	Masculino	1240	8	ativo	25.35154	1483.62	1615.484	835	990.2576
919	52	Feminino	1240	8	insuf. ativo	27.34375	1332.75	1489.807	1095.72	1207.127
920	20	Feminino	1240	8	ativo	23.62445	766.38	987.8912	750.82	916.9172
921	66	Feminino	466.25	4	muito ativo	35.99633	2399.605	2329.042	2036.835	1822.277
922	43	Masculino	475	8	ativo	27.1809	1487.04	1618.301	1479.47	1504.755
923	80	Feminino		11	sedentário	21.63115	1121.74	1308.989	904.39	1049.395
924	62	Feminino	650	8	ativo	24.34964	667.11	892.9386	643.89	820.5999
925	57	Feminino	7500	13	sedentário	26.89232	1559.68	1671.783	1919.655	1821.074
926	75	Feminino	207.5	0.5	sedentário	29.72839	2318.84	2269.935	2045.34	1910.776
927	61	Feminino	2100	13	ativo	23.43374	1347.985	1491.428	1481.1	1501.314
928	77	Feminino		5	sedentário	24.67702	1253.4	1422.545	1199.73	1290.021
929	61	Feminino	10500	13	sedentário	23.30906	1767.865	1844.859	1548.355	1556.027
930	53	Feminino	2426.667	8	insuf. ativo	29.29688	2526.235	2340.986	1804.945	1711.964
931	61	Feminino	700	4	sedentário	28.98114	1792.18	1862.729	1301.44	1363.004
932	67	Masculino	857.5	0	ativo	32.66077	1302.58	1464.332	834.24	989.6036
933	43	Feminino	142.5	6	sedentário	22.04779	954.37	1049.438	1135.58	1134.532
934	33	Masculino	500	7	insuf. ativo	24.91349	2150.72	2075.282	2125.41	1914.051
935	33	Feminino	500	7	ativo	37.27907	1055.28	1235.391	1027.775	1144.222
936	25	Feminino	174.2	7	insuf. ativo	20.3125	1971.97	1890.712	2559.31	2244.198
937	52	Feminino	726.6667	8	muito ativo	22.58955	1184.96	1349.022	1318.515	1382.401
938	27	Feminino	726.6667	8	muito ativo	22.46003	2696.655	2531.132	2173.72	1965.889
939	66	Masculino	321.2	3	ativo	29.38476	1678.915	1771.048	1205.345	1290.756
940	50	Feminino	185	8	sedentário	29.31986	1043.455	1103.114	811.8	848.8408
941	27	Feminino	185	8	ativo	24.14152	2504.58	2406.066	1687.725	1608.392
942	37	Masculino	311.25	4	ativo	27.74475	3165.32	2882.042	2485.08	2206.711

943	29	Feminino	311.25	4	insuf. ativo	21.00767	2922.67	2710.733	2046.8	1911.784
944	36	Feminino	569.1667	4	insuf. ativo	28.60476	2118.32	2118.22	1272.72	1347.141
945	40	Masculino	2090	11	ativo	23.25502	1622.9	1729.165	1132.52	1236.667
946	63	Feminino	2900	13	muito ativo	21.82995	1649.695	1705.259	1954.92	1839.753
947	71	Feminino	1800	8	ativo	25.59374	1766.295	1839.358	2150.37	1980.847
948	42	Feminino	1896.667	11	sedentário	30.46875	2320.62	2271.268	1599.24	1593.537
949	43	Feminino	376.6667	7	muito ativo	29.5858	1115.23	1301.681	766.27	929.912
950	82	Feminino	683	4	sedentário	19.56296	650.82	877.7557	632.65	810.5859
951	81	Masculino	447.4	7	ativo	33.64198	1560.59	1656.965	1098.06	1198.887
952	72	Feminino	447.4	7	insuf. ativo	26.53376	1064.075	1205.416	976.275	1102.064
953	67	Masculino	415	3	ativo	26.44628	1871.67	1927.274	1488.43	1511.457
954	67	Feminino	415	3	insuf. ativo	34.62682	1853.95	1913.356	1838.65	1766.212
955	80	Masculino	400	4	ativo	30.32334	1056.39	1251.643	1133.34	1237.322
956	82	Feminino	400	4	insuf. ativo	24.91077	1143.16	1327.639	1284.84	1356.547
957	75	Feminino	416.25	4	insuf. ativo	32.8473	765.72	985.7162	918.625	1061.289
958	31	Masculino	233.3333	11	sedentário	31.85596	762.46	984.2081	677.34	851.3015
959	32	Feminino	233.3333	11	insuf. ativo	28.959	2357.085	2212.587	2057.43	1765.96
960	60	Masculino	500	5	muito ativo	30.47052	1092.31	1278.807	963.95	1097.792
961	61	Feminino	343.75	4	insuf. ativo	23.66144	1314.28	1474.225	1025.63	1150.185
962	65	Masculino	343.75	4	ativo	20.57143	1291.11	1454.615	1335.59	1395.697
963	20	Feminino	343.75	4	muito ativo	21.63115	2007.07	2032.72	1474.16	1500.778
964	38	Feminino	1025	11	insuf. ativo	20.20202	2034.285	2037.988	1682.545	1653.745
965	48	Masculino	1025	11	muito ativo	22.09317	3296.915	2922.193	3726.19	2955.466
966	20	Feminino	1025	11	sedentário	17.31341	1328.685	1481.105	1095.94	1206.666
967	29	Feminino	1366.667	13	ativo	24.21875	1666.46	1731.975	1318.725	1332.475
968	31	Masculino	1366.667	13	ativo	25.71166	4040.105	3386.872	2812.035	2328.619
969	80	Masculino	733.3333	4	ativo	26.72993	1561.755	1674.968	1270.045	1312.657
970	76	Feminino	282.5	1	sedentário	30.84284	1446.355	1577.243	1334.56	1394.519
971	33	Feminino	279.8	8	ativo	24.08822	1050.1	1246.087	920.83	1063.244
972	42	Masculino	279.8	8	muito ativo	22.22222	451.46	678.9116	263.92	442.0965
973	42	Feminino	2814.286	13	muito ativo	26.79494	1431.5	1556.377	912.595	1025.138
974	58	Feminino	1028.75	10	muito ativo	31.25	1663.2	1728.37	1133.46	1217.719
975	69	Feminino	725	11	muito ativo	22.83288	2112.395	2090.223	2280.715	2066.573
976	48	Masculino	600	11	sedentário	25.60554	1601.575	1686.92	1478.11	1501.618
977	42	Feminino	600	11	muito ativo	28.01022	789.88	1008.615	945.735	1081.41
978	62	Masculino	650	4	ativo	35.56395	2087.79	1982.912	1946.695	1770.477
979	62	Feminino	650	4	insuf. ativo	26.22269	1490.305	1620.341	1286.62	1352.597
980	64	Feminino	166.6667	4	muito ativo	18.31426	1075.09	1243.444	1072.195	1144.603
981	31	Masculino		11	muito ativo	28.08217	1816.64	1883.958	1553.77	1560.033
982	49	Feminino		4	sedentário	27.1809	1629.37	1734.395	908.67	1053.006
983	68	Masculino	662	4	ativo	26.29758	1943.355	1953.231	2206.265	1858.365

984	62	Feminino	662	4	insuf. ativo	22.37034	1772.51	1833.41	1526.6	1530.186
985	69	Masculino		11	muito ativo	24.224	2099.84	2104.086	2690.02	2339.994
986	66	Masculino	350	4	sedentário	39.44208	1292.78	1456.031	904.85	1049.783
987	60	Feminino	350	4	insuf. ativo	29.82325	1291.04	1454.556	908.56	1052.913
988	71	Feminino	343.6667	8	sedentário	18.81892	1220.92	1394.765	1031.39	1154.899
989	72	Masculino	450	4	sedentário	25.88057	1023.28	1208.5	946.41	1068.782
990	70	Feminino	450	4	insuf. ativo	36.85307	678.15	894.0699	553.64	732.1127
991	74	Feminino	500	4	ativo	23.30906	1202.48	1369.887	1198.56	1287.927
992	65	Masculino	750	4	muito ativo	25.51021	1780.13	1855.065	1384.81	1433.317
993	65	Feminino	750	4	insuf. ativo	28.93518	3008.44	2771.633	2465.97	2194.143
994	64	Masculino	550	11	muito ativo	25.1559	1836.115	1858.195	1287.805	1316.598
995	38	Masculino	450	8	ativo	30.11938	1401.54	1547.455	1450.92	1483.331
996	48	Feminino	525	9	ativo	20.95717	1630.825	1726.429	1409.245	1427.722
997	32	Masculino	383.3333	8	muito ativo	22.94812	1464.055	1599.072	1726.235	1682.386
998	31	Feminino	383.3333	8	ativo	21.64412	1190.04	1361.07	2126.69	1942.129
999	50	Feminino	550	1	insuf. ativo	25.71166	1385.095	1533.711	1271.455	1343.944
1000	74	Feminino	372.5	0.5	insuf. ativo	31.14187	454.44	681.9809	473.68	659.9081
1001	52	Feminino	700	5	muito ativo	18.72417	1620.9	1727.547	1242.2	1323.357
1002	47	Feminino	700	5	insuf. ativo	35.51136	1772.08	1622.233	1388.71	1156.552
1003	52	Feminino	620	8	muito ativo	25.53671	1954.69	1962.497	1285.015	1241.13
1004	28	Masculino	318.75	7	muito ativo	22.53086	2146.09	1994.413	1339.96	1344.111
1005	23	Masculino	318.75	7	muito ativo	20.19509	2439.07	2359.53	1742.21	1697.375

id	energy_density	alcoholicbev_portion	beans_portion	breads_portion	butter_portion	cakes_portion	cheese_portion	coffee_portion	coldcuts_portion	eggs_portion
1	1.110746				15			50.05		45
2	1.585495	187.505		50						
3	1.454453		107.5	50	30		55	96.09		
4	2.067685			115			60	50.05	45	
5	0.8731734		107.5					176.83		
6	0.9073365			42.5				240.22		
7	1.696159		86					100.09		
8	1.134732		86	75	15			114.1033		
9	1.372135		64.5	75	15			105.7633		
10	1.295244			50			70			
11	1.521498			37.5	15		70	240		100
12	1.175569		64.5	20	35.625	60	34.66667	112.1033		
13	1.164808		86	53.33333	12.5			127.2817		
14	1.16011		215	100	15		15	120.11	34	
15	1.130792		105	104	18.75			80.07		
16	1.084736		107.5	52				120.11		
17	1.220291		139.75	93.66666	45			144.8033		
18	1.240497		78.6	50	15		48.33333	70.06667	30	
19	1.370572		52.4	58.33333	7.5		32.5	97.59	15	
20	0.7949743		107.5	150	22.5			333.31		
21	1.11814		86	26				57.38		50
22	1.010273		32.76							
23	1.363244						55	80.07		
24	1.604332			50		60	15	120.11		
25	1.447385		179.1667	62.5	11.25			90.8375		50
26	1.129985		86	75	15	60		180.1675		
27	1.32368		81.88	58.33333	26.25			108.0667		
28	1.283911		67.75	27.5	11.26		25	97.09		
29	1.223752		137.25	50	15		15	166.8267		50
30	0.990928	703.01	63.6					112.4367		
31	0.9730501		63.6			60		62.72667		
32	1.538592		26.22	50	37.5		40	150.8067		
33	1.377161		107.5	250	56.25			1153.06		
34	1.298621		82.3	50				75.07		
35	1.610357		43	50			20		34	50
36	1.211003		65.6		12.5	120		165.4833		
37	1.305522			25			30			
38	1.055046		32.76	50	7.5			49.045		
39	1.214182			50			10	75.07	15	
40	1.029331		86.26	30	15		29.33333	288.27		

41	0.9273288		258	50			80			
42	0.889724	472.0267	87.5	130	60			198.5167		
43	1.14161			54	26.25		20		30	
44	0.8133891	477.045		27.66667	18.75		75	291.27	15	75
45	1.256593		43	64	18.75			186.1511		
46	1.578908			100				50.05		
47	1.352531			73			19	50.05	17	
48	1.357057		86	50			20			50
49	1.217101		86	25		60	40	225.21		
50	1.295915						30	107.6		
51	0.8877156		58.95	20			7.5	168.16		
52	1.806879		86							
53	1.308588		86	30	45			300.28		
54	1.224232		129	45	7.5			80.07		
55	1.170588		98.26					50.05		
56	1.068213		122	50	7.5			185.1733		45
57	1.185911		199	50	15			153.1433		45
58	1.004103		65.5	50	15			192.18		
59	1.187647			20				48.04		
60	1.25307		263.375	50			15	210.1925		100
61	1.073133	1506.45						144.13		
62	0.928941		56.1	50	7.5		20	94.08749		
63	1.20852	1205.17	164.8333	69.33334	32		50		30	
64	1.348827		140	100	15			97.09		
65	1.080849		129	200	7.5			92.95389		
66	1.469462		258	37.5	15			96.09		
67	1.234787		107.5	50	18.75			96.09		
68	1.413205		86	22.33333			20	48.04		
69	0.8381184		75.25					180.1675		
70	1.248461	1054.52	107.5				30	136.1233		
71	1.215163		93.66666					144.13		
72	1.566866		64.5			120		175.6656		
73	1.389384		86			100		192.18		50
74	1.489025			50	7.5			96.09		50
75	1.092051		86	50			20	75.07		
76	1.160617		161.25	75	4.75		10	96.09		
77	1.049612		96.75	50				160.1467		
78	1.602798		30	60						
79	1.244975		105.65	68.75	18.75		20	57.055	17	
80	1.155707		86	91.66666	22.5	60	15	96.7025		
81	1.022531			30			15	160.15	16.5	16.5

82	1.05672	703.01	52.4	50				44.36833	34	
83	1.084996		107.5	75			15		15	
84	0.7087841		32.76							
85	1.173142		83.315	50				114.1033		
86	0.9675596	44.74	71.66666	60		100		276.6843		50
87	1.389948		129	50	15			50.05		
88	0.9676392	250.315	75.25	50	26.25			142.6325		
89	1.085482		86	25	7.5			84.305		
90	1.136492		129	40	15			160.15		
91	1.127596			62.5	11.25			96.75667		
92	1.086803	477.2	172					100.09		
93	1.135365		107.5	50		30		123.115		
94	1.217237		39.3	50	15		30	110.6033		
95	1.176975		215	50						
96	1.271372		96.75	83.33334	22.5		40	107.5717	30	
97	1.129029		86	100	7.5			100.09		50
98	1.238628		88.43	50			22.5	37.535		
99	1.35701	58.75	96.75	100	26.25			258.24		75
100	1.201512		105.62	50	12.5			82.075		50
101	1.208802		43	37.5	10	30		62.56		
102	0.8817567		58.45	25				122.585		
103	1.468772		53.75	91.66666	18.75			123.1133		
104	1.458939		78.83334	50	11.25	100	15	144.135		
105	1.279122		71.88	101.6667	26.25	60		72.565		
106	1.217691		100.3333	53.33333	6.75			66.3125		50
107	1.31925		86							
108	1.219731		86	27	10			112.605		
109	0.8901556			50				96.09		
110	0.7848913		107.5	70		30	15	390.16		
111	1.300164		52.4	25	11.25	30	5	182.67		
112	1.234714		31.33	75	15			81.97945		
113	1.391093		129	100	7.5		30			90
114	1.077793		86	62.5			30			
115	0.7209952			45			40	78.07		
116	1.318329	703.01	258	50	40		37.5	70.565		
117	1.198047		258							
118	1.264863			35	22.5			109.6		
119	1.148104		43	30			20	134.625		
120	0.9228683		86	50				96.09		
121	1.45942			50	7.5			47.545		
122	1.084939		147.42	100	30			170.1567		50

123	1.209557		129	137.5	82.5					
124	1.153593		101	81.25	30			255.5014		
125	1.400703	148.36	34.6	37.5	25			85.08		
126	1.423606		96.75	110	40					50
127	1.055307		86.75	50	7.5			140.5344		
128	1.37305		86	66.66666	10.83333			90.75		
129	1.142961		71.55	50	8			86.58		
130	0.9836811		86							
131	1.35271		107.5	50	7.5			60.06		
132	1.508466		150.5	100			60	120.11		
133	1.106959		107.5	75	7.5		60		45	90
134	1.23576	1054.52	43	60			37.5	48.04	30	
135	1.256747		86	50			22.5		30	90
136	1.033642		107.5					50.05		
137	1.329159		43	50	15					
138	0.9391053									
139	1.964105	47								
140	2.015233			200			30		30	
141	2.425304			125			20		15	
142	1.244564		30.43333	45	10		180	73.06667		
143	1.189859		86	50				127.12		
144	1.24897		43	50	10			100.09		
145	1.395402		78.83334	162.5	46.875			87.58		
146	1.247471			75	7.5			84.075		
147	1.356417		43	75	11.25	120		137.46		
148	1.110493		40.885	25			17.5	69.73		
149	1.274507					120		100.095		
150	1.28346		93.16666	50	15	200		158.65		50
151	1.265202		172	120				111.105		50
152	1.216191		78.75	75	18.75			145.334		45
153	1.57566	351.51	258	48.75		150	40	106.2225	30	50
154	1.507278		43	50			40	120.11	30	
155	1.015713		37.88	41.66667	11.25			84.08		
156	1.019668					60		102.8467		
157	0.8463845							529.3489		
158	1.401946		208.95	100		90		22.52		
159	1.065754		139.75	27.5			16	125.12		
160	1.175794	1205.17	129							
161	1.087619		41.1	50	12.5			155.14		
162	1.140966	1205.17	93.16666					133.625		100
163	1.221081						7.5			

164	1.509164		78.6	50	22.5		20	25.02		
165	1.216927		96.75	125	20			154.5175		
166	1.069566		100	50				216.2		
167	1.213573		258	25				73.57		
168	1.704846							73.57		
169	1.069929		86					180.165		
170	1.554998		56.5	110			43			
171	1.289996		86	100	30			39.035		
172	1.423834			30	15			61.06		
173	1.267446			58.33333	11.66667			53.54667		
174	1.339115		86	50	7.5			48.04		
175	1.052491		107.5	75				96.09		
176	0.8579765							360.33		
177	1.207213		86	50	7.5			72.07		
178	1.316312		476	50	40		30	72.07		
179	1.312539		107.5	50			55.25	120.11		
180	1.379431		59	50	6	29.99		83.325		
181	1.56511							72.07		
182	0.9857157		172					264.24		30
183	1.369535		64.5	50						
184	1.248106	351.51	129	37.5	7.5			72.07	30	
185	1.108672		52.4	50				51.295		
186	0.9360152		104.8					16.62		
187	1.229328		86	50	22.5			195.18		
188	0.8524001	2109.04		50						
189	1.093997		43	50				66.66666		30
190	1.384661		65.6			60		107.2011		
191	0.8536687		43	37.5		60		69.565	30	
192	1.705565		104.8					72.07		
193	1.165112		86	40	7.5		30	90.8325		
194	1.14496		114.6667	50	7.5		45	73.07		
195	1.057713		100.3333	50	7.5		60	112.77		
196	1.092128		86					50.04		50
197	0.9857618		172					144.13		
198	1.631123			30			17.5	192.18	5	5
199	1.040592		32.26	50				75.07		50
200	1.0738		107.5	100				83.325		
201	1.174448		123.63					192.175		
202	1.015213		64.5	50				133.62		
203	1.328494		80.625	43.33333				45.04		
204	1.155619	351.51	129	53.33333	15			143.635		

205	1.57971		65.5	75	15			72.07		
206	1.210261		78.6	100	15			108.1		
207	1.359504		344	100				144.13		
208	0.8070871			25				240.22		
209	1.300465			50	15			144.13		
210	1.171974		64.5	50				72.07		
211	1.290154			83.75	7.5		30	276.26		50
212	1.213842			68.75	7.5		30	135.6275		
213	1.167692	326.52		50	22.5	30	21.8	123.115		
214	0.9848767		43.79	50	7.5			104.3475		
215	1.367673		61.13334	50	20.83333			75.07		50
216	1.501979		210.97	125	22.5		30	133.125		
217	1.187209		102.25					400.36		
218	1.188609		139.75	70	3.2			162.82		
219	1.196356		114.6667	50				118.11		
220	1.272895		172	100			80		60	
221	1.236592		32.76	50	6.25		185	25.02		
222	1.524322	1054.52	26.2					192.18		
223	1.092771		143.3333					80.07		
224	1.051557			45	11.25			160.145		50
225	0.800336		129	60				100.09		
226	0.8296735		110.6667	30	5			123.115		20
227	1.41488		65.53333	50	18.75			74.32		
228	1.181409	351.51	78.53333	50	5	60		195.18		
229	1.169616		98.3	50	7.5			192.18	15	
230	1.48858		65.6	100	15			75.07		
231	1.102791		73.4	66.66666				80.07333		
232	1.067213		73.4	66.66666				80.07333		
233	1.26215		227.4	50			20	121.11		
234	1.092289	208.81	61.2	42.5	10			88.3325		
235	1.275173		172	25			30	150.14		
236	1.229649		26.2			60				
237	1.285753			25			20	118.61		
238	1.42709		107.5					100.09		
239	1.22206		96.75	50	11.5	40	20	150.5374	15	
240	1.139772						60	144.13		50
241	0.8068632			50			22.5	85.08		
242	1.473972					50		90.75		
243	1.341266		100.3333	47	7.5			96.09		
244	1.535159		98.54			30		130.62		
245	1.177683		64.5	50	7.5			48.04		

246	0.9349295		69.2	50	10.83333		18	110.94		
247	1.149046		96.75	150	45			206.695		
248	1.179707			60	15	90		146.135		
249	0.9711918		86	50			20			
250	1.250509		98.26	50	7.5		20	160.14		
251	1.134714		52.4	32.5	7.5	60	16.25	235.8267		
252	1.343181		100.3333	43.75	22.5			83.86285		
253	1.07155		78.6	37.5	7.5			247.735		
254	1.080882			29	15			104.0933		4
255	1.119155		65.6							
256	0.8203422					18		150.14		
257	1.130862		75.25	75				139.6275		50
258	0.9456141		64.5	50			20	123.4467		
259	1.086049		86					258.9066		
260	1.231357		53.75	55			15			
261	1.241705	1205.17						120.11		50
262	1.255812		75.25	100	15		90	45.04		
263	0.8237185		26	37.5	7.5		7.5	180.1675		
264	1.177322		46.13334	82.5	20.625		20	231.55		
265	1.275471		242.3933	100	30			126.6175		
266	0.9312384		86	50				91.08333		
267	1.037569	351.51	107.5					25.02		
268	1.118864		107.5	25	7.5					
269	0.923289		107.5		22.5		56.25	248.592	22.5	
270	1.019219			60			22.3			
271	0.995142		175	50			40	60.06	30	
272	0.9587576		65.6	25			15	100.09		
273	1.031777			43.33333			16	112.105		
274	1.606296		100	100	37.5			75.07		
275	1.620073		65.6	50	15			72.07		5
276	1.153183		75.805	20				63.32278		5
277	1.092652		525							5
278	0.9569489		52.4					90.08		25
279	1.156797		43					186.2767		
280	1.593962		43	25	15			67.54		
281	1.253068		86	20	30		25	50	18.75	
282	1.075007			50	7.5		15	112.605		
283	1.477932		70	58.33333	10	179.94		64.05666		
284	1.102274		86	100				192.18		
285	1.136618		86	50	7.5			50.05		
286	1.086128		68		20			82.075		

287	0.6765906			50	7.5			96.09		
288	1.149925		104.8	50	6.02			144.135		
289	1.323284		65.6	96			60	96.09		
290	1.584134		43					72		
291	1.308968		86	75	22.5			100.09		
292	1.168926		65.6	50	17.8125		22.5	171.8267		50
293	1.368808		157.5	50	15		20	67.565	15	50
294	1.341509			116.6667	11.25			102.595		
295	1.168417		26.2	100	22.5			125.12		
296	1.213569		53.75	50	13.125	60	30	152.14		
297	1.163627		86	50			40	89.08	30	
298	1.420081		436	58.33333	50.5			362.835		
299	1.13103	78.1	51.065	50	11.25	37.5	15	252.2325		
300	1.206324		68	110			22.5	104.5975	15	
301	1.26886		43	93.75	7.5		47.5	61.555	30	
302	1.52913	351.51	107.5	50	15			120.11		
303	1.621206		86	50	11.25	100		25.02		
304	1.133158		107.5	100				120.11	15	
305	1.721816		64.5					96.09		
306	1.42425		114.6667	50	12.5			77.325		
307	1.847201		140.26	50						50
308	1.321014		172	50	22.5			144.13		
309	1.075093	703.01	215							
310	1.090976		344			100		125.12		
311	1.168306		344			100		66.72		
312	1.100033		64.5					100.09		
313	0.9591058			150						
314	1.279578	433.86		50	15			45.04		
315	1.129688			48.5	8			200.18		
316	1.031721			55	11.5		20	125.12		
317	1.222539			86.66666	19.5			250.23		37.5
318	1.062711			30	7.5			200.18		
319	1.199667			100	15		40	72.07	40	20
320	1.095285			30		60		100.09		2.79
321	0.9167207	351.51	51.9	50	10.25		22.5	95.085		
322	1.341925		131	50	23.5	180		122.4467		
323	0.9055203		78.6	30		41				
324	1.070593		182.6	50				37.53		
325	1.247171		131	50	90			62.56		
326	1.167813			50	15		15	180.17		
327	1.125718	125.375	130.5	50		60		90.08		

328	1.098566	175.75	87.5			117.5				
329	1.621116			75	7.5	30	20	111.1033		
330	1.820478							126.1167		
331	1.2169			37.5	7.5		30	36.03		20
332	1.433999		52.4	50	15		20	97.09		25
333	1.287856			50	7.5		40	72.07		3
334	0.8382934	356.53								
335	1.483879		107.5	60	15		35	80.07		
336	1.177208		102.88	49	7.5			59.305		12
337	0.9690455		64.5	30			10	45.04		
338	1.036193		72.96667	38.5			17.5	190.675	25.5	
339	1.46557		52.5	100	37.5		80	73.57	30	
340	1.340536		92.33334		33.75		30	192.18		
341	1.323276	53.68	86	84	60			80.03378		
342	0.9933661		75.25	50			35.83333	115.105		
343	1.595849	713.06	322.5	100	60			160.15		
344	1.331236		301	100	30		30	72.07		
345	1.327834		26.2	50			22.5	108.1		
346	1.022525		126.2	50				103.595		
347	1.367877		93.88667	43.75	32.5		20	49.045		50
348	1.515476		85.2	50	28.125			90.08		50
349	1.06657		172							
350	1.220465	1406.03	129	100						
351	0.626325		86					494.858		
352	1.223527		400	300	30					
353	1.048817		29.38	55			30	94.8375		
354	1.076535		86					75.07		
355	1.460919		172	87.5	18.75			112.605		
356	1.222863		86	50	12.5			158.65		
357	1.285214		215	100	30			216.195		50
358	1.188518		172	50				109.6		
359	1.458737		86	100	28.125		40	96.84	30	50
360	1.405775		86	100	30		40	122.1133	30	
361	1.424247		96.75	100	60		30	132.575	22.5	50
362	1.813806		86	100	60			480.44		
363	1.582548		86	50	15			107.502		
364	1.352451			50	30			75.07		
365	1.214808		52.4	75				168.155		
366	0.984234	1054.52	229.3333					150.14		150
367	1.250201		94.66666	41.66667	15			107.915		
368	0.9124558		64.5					108.1		

369	0.7042356		86					560.515		
370	1.308622		172	100	10			132.62		
371	1.001114		29.48					144.13		
372	1.356917		86							
373	1.333311		43	50	15			96.09		8.33
374	0.9493632		86	50				216.11		
375	1.17528		46.13334	75				139.38		
376	0.7305636		64.5					90.085		
377	1.042825		32.24	50				112.1033		
378	1.294674		300	67.5	8.75			158.65		
379	1.032765		65.6	100				214.8667		
380	1.13539	1205.17	107.5	50	26.25	65		114.105		50
381	1.173388	149.96	86	66.66666	15		40	90.75333	30	
382	1.608897		70.2	50		124.95	45	236.22		
383	1.543042		48.1	50		74.98	25	95.08666		
384	1.51346			50		120		50.05		
385	0.9465188			48	7.5		62.5	128.4333		
386	1.419647		70	50	15			240.225	80	
387	1.28176		129							
388	1.596246		139.75	100	20			142.635		
389	1.212351		86	100	15			209.5267		
390	1.154955		86	50	15			66.059		
391	0.7904491			30	5.62			55.85333		
392	1.315776		96.75	50	11.66667			146.135		
393	1.271897		215	100	15			72.07		
394	1.264725		72.48					101.595		
395	1.084996		139.8	56.66667			15	192.18	17	
396	1.313917		157.2	50	7.5			49.38		
397	1.395898		43	50	8.75			63.06		
398	1.131692		173.5					264.24		50
399	1.033266		129	37.5				72.07		
400	1.325847		107.5	50	22.5			64.06		
401	0.9530115			30			22.5			
402	1.283314		140	65	10		20		40	
403	1.303173		172							
404	1.419543			50	22.5				62	
405	0.8406181			50			40		15	
406	1.385527		26.2	50		30	30	107.1	15	
407	1.343822				7.5			81.07666		45
408	1.392442		172	100		180		288.265		
409	1.574932		86	50	7.5	60		25.02		

410	1.258858		271.25	100			100	122.615		
411	0.8383901		43	50			20	72.065		
412	1.104575		215	50	7.5					
413	1.393374		86	50	22.5			73.07		
414	1.393374		86	50	22.5			73.07		
415	0.9358857			50.41667	20			169.655		50
416	1.124259	351.51	258	50				73.57		50
417	1.247395	527.26	86	100	30			75.07		
418	1.188432			20						
419	1.487945	351.51		70			20	192.18		
420	1.09701		64.5	50	7.5			224.21		
421	1.02884		86	50	15			78.075		
422	1.038441		129					96.09		
423	1.242793		43			50.5		122.11		
424	0.8979577			20	15			50.05		
425	1.222434		90.66666	50				45.04		
426	1.216459		107.5	50	12.19			90.085		
427	1.316573		86	66.66666	7.5		41.33333	216.2		
428	1.489041			25		180				
429	1.11243			27	7.5	30		144.135		
430	1.473477		65.6	150	25	238		116.9433		
431	0.9931661		100.3333	50	7.5			696.645		
432	1.237744			50		100		85.575		
433	0.9569188		43	50	5			125.12		
434	1.204624			75	8			232.22		
435	1.209043		215			60		96.09		
436	1.510246		75.64					108.1		
437	1.133853		136.1667	50	15			85.58		
438	1.017239	351.51	225.75	50	26.25			240.22		
439	1.307867		52.4	50	30			125		
440	1.309845							120.11		
441	1.076404		107.5	75				190.1767		
442	1.210029		107.5	50				84.08		
443	1.133717		215	50						
444	1.325862		236.5	75	28.25			226.8733		
445	1.425376		172	50	15			240.22		
446	1.676197	401.72	107.5	50				96		
447	1.322926	200.86	86					96.09		
448	1.111052		258					120.11		
449	1.165119		107.5	50	7.5	50		45.04		
450	1.103262		41.15	54	15			175.16		25

451	1.226371		107.5					67.56		
452	1.482647		107.5	50	16			45.04		
453	1.507403		32.76	75	10		40	85.58		
454	1.321989		131.25	100				72.07		
455	1.211527		172	50		100		144.13		
456	1.273857		172							
457	1.288611		172	50	7.5			109.6		
458	1.061743		86	50				108.1		50
459	1.482974			50	22.5			75.07		
460	0.8815334		100.44							
461	1.322002					60	24.2			
462	1.119863	298.25				100	17.6			
463	0.905354		135.13	30	15			123.8675		
464	0.9983516	1807.75	129					125.12		
465	1.153678		86					96.09		
466	0.7226501			37.5				192.68		
467	0.8336887		226.2	50				192.1767		
468	0.770723	703.01	172	110				200.1833		65
469	1.14783		172	110	7.5			96.09		
470	1.380596		89	62.5	9.375	120		86.3325		
471	0.8632726		86	150				72.07	30	
472	0.894217			25	15			169.49		
473	1.659277		157.5	50	19	30	40	33.33333		
474	1.279563		114.6667	84.66666	22.5	120		250.8967		
475	0.8932084		42.6	30.25		60		480.44		
476	1.348117		52.4	25	6.02			126.66		
477	0.9520743		86					50.05		
478	0.7806746			25			0.35	392.8625		
479	0.9608657		43	50	15			176.1633		
480	1.714382		107.5	100	30			100.09		
481	1.929726			120	7.5	200	15	45.04		
482	1.646588		107.5	100	30			25.02		
483	1.539426		172				40	45.04	30	
484	1.406431		160.5	150	22.5			125.12		45
485	0.9773588		105	50	15			112.77		
486	1.409037	23.5	86	43.75	22.5		15	125.12		
487	1.054584		113	47.5	50		20	207.19		
488	1.258253							50.05		
489	1.39543		75.25	50			120	82.58		
490	1.256824		78.6	50				130.12		
491	1.280522		52.4	100	15			98.09		

492	0.9759643	492.1767	175					144.13		
493	2.119406		129				20			
494	1.63146			50	111.25			207.1933		
495	1.026602	821.54		50						
496	1.095653		65.5	20				64.50333		
497	1.213944		105	50				100.09		
498	1.558846		86	50	15			160.15		
499	1.089179		74.39	100	15			120.11		
500	0.8876919		108.5	50		30		75.07		
501	1.480759		129	50	22.5			75.07		50
502	1.124962		215	50	30			74.07		
503	1.2555		43.46667	50	30			122.1133		70
504	1.054795			50	15			377.6833		75
505	1.303216			150				249.23		
506	0.9470091	703.01		60	7.5		32	192.18	34	
507	1.181713		39.3	54				72.07		
508	0.9400153		52.4					80.07		
509	1.344871	144.62		30			60	45.04		
510	1.359052		43	70				133.625		100
511	1.024596			25	7.5			120.11		
512	1.063588		50.77	35.66667	15			179.5033		
513	0.9946227			45			30	192.18		
514	1.119636		82.3	30	7.5			118.61		
515	1.266976			16.6	3.2					
516	1.451824		200		25	30		33.33333		
517	0.8028865		52.4	30			70	33.33333		
518	1.70485		104.8	50	12.66667			119.61		
519	0.7850365						90	166.65		
520	0.9381179		26.2	45	7.5		80	576.53		
521	1.274121		26.2		7.5			100.09		50
522	1.071382		205.6	50	15			126.615		
523	1.111913		64.5	50			20	128.12		
524	1.087222	351.51	64.5	50	15		33.33333	1226.107		
525	1.009523		66.29333			30		72.07		50
526	0.8842042			50	22.5					
527	1.304146		179.13	100	30			121.11		
528	1.197613		236.5					192.18		
529	1.160729			49.33333	12		45	74.07	15	
530	1.376411		64.5	50	10		8	76.07		
531	1.088371		130.7933					90.0825		50
532	1.18299		84.20667	36.66667	7.5		15	67.56		50

533	1.177613		32.26	37.5			20	96.09		
534	1.833056			50	15			82.57		
535	1.183832		624	50				40.04		
536	1.174567		55.68	31.25	13.125		22.5	70.06667	22.5	100
537	1.12772		49.18	41.66667	12.5			97.59		
538	0.925458		58.96	27.5	15			77.32		
539	1.230176	94.01	92	42.5	7.5		45	96.09		15
540	0.6061076							80.07		50
541	0.8198444		78.6	50		120	22.5	104.0967		
542	1.338791			60	22.5		40	35.55556		
543	1.119429			56.66667			24.375	48.98963		
544	1.297043			83.33334	22.5			96.09		
545	1.41153			37.5	15			157.1433		
546	1.509329							45.04		
547	1.326574			75	20.625			130.1175		
548	1.043374		71.55	62.5	7.5		25	76.07		
549	1.232834		120.0867	50			40	326.7		
550	1.450083		78.6					240.22		
551	1.160867		35.96	50	7.5		20	90.08		
552	1.029125		83.06667	62.5	20.625	64		116.666		45
553	1.225879			50	15	60	30	120.11	30	
554	1.052891			50	10	73.92	35	167.155		
555	1.095497		107.5	50			90	288.27	68	
556	1.203416		64.5	50	11.25		30		25.5	
557	1.003777			50			90	160.15	34	
558	1.073329		114.62	30				175.16		
559	1.046932		98.3	30				136.125		
560	1.315291		132.5	60	24.5	30	40	112.7567		
561	1.579311		52.4	50		100	52.5	63.3075		55
562	1.431325		86							
563	1.326689		86	75			30		30	
564	1.486489		229.3333	100	15			112.605		50
565	1.03243			50	7.5			80.07		
566	1.180666		86	110	7.5		80	105.0933		
567	1.111431			50	7.5			256.24		
568	1.242218		78.6	50			15	134.625		
569	1.038517		58.06667				23.33333	16.14815		37.5
570	1.408018		86	50			22.5	123.7161		
571	1.117066		65.6	24	17.5	100		105.5415		
572	0.930823			33.33333	7.5		10	113.4367	7.5	25
573	1.368418		75.25	62.5	37.5			90.08	35	

574	2.108812		78.6	50	45		7.5	12.01		
575	1.039998							37.53		
576	1.048307		75.8	37.5	26.25			74.068		
577	1.422716		52.4		15			181.53		
578	1.228572		52.4	40				93.8375		65
579	1.253208		86	56.66667	10.5	60	20	97.59		
580	1.266359		127.3333	50	7.5		40	81.07333		
581	0.9879154		78.66666	50	10	60		143.385		
582	1.053698	496.125	96.75	100	6		17.5	96.09	15	
583	1.236749		54.25	47.5	19		20	128.12		
584	1.017367		65.6	30	7.5			250.23		
585	0.9062844		147.38					70.565		
586	0.9116347		58.96	50	15	60	36.25	153.8925	10	
587	1.202993		344	100	7.5			73.07		
588	1.614551			50	7.5			96.09		
589	1.10259		43	200			18		10	
590	1.201492		196.5	50	15		30	100.09	45	
591	1.346484		67.19	37.5	7.5		40			50
592	0.9909623			25			30			
593	1.202995		118.25	53.5	4	30		88.08334		
594	1.237245	149.12		25	7.5		60	100.4267		12.5
595	1.213253	150	64.5	66.66666	45			100.09	100	
596	1.209864			75	7.5		30			
597	1.501179		107.5			60		120.11		22.5
598	1.157188		87.5	52.5				120.11		
599	1.158952		48.38	20			15	120.11		65
600	1.152418			100				65.06		
601	0.9305218	351.51		95			53.33333	205.0457		
602	1.447404					160		100.09		
603	1.371645			100				72.07		
604	1.513649		32.26	50				45.04		
605	1.560512		43					96.09		
606	1.511549		215	66.66666	22.5		29.5	144.13	30	
607	1.234232		107.5	50	30			80.075		
608	1.334751		107.5	50			80	82.575		
609	1.713725		215							
610	1.2831			50		60	7.5			
611	1.296331		42	50				25.11111		
612	1.313589		86	50			90			
613	1.200572	241.035	350	37.5	13.125		23.33333	192.18	20	
614	1.642441		86	50	15			141.13		

615	1.186086		26.2			65	16.13	123.1125		
616	1.019989	2586.085	129	50	38			108.1		50
617	1.203727	250.315		50				192.18		50
618	1.070799			30				192.18		50
619	1.164812	410.09	107.5	100	15			46.295	30	
620	0.9393601		64.5	60			15	50.71333		
621	1.522314		86	73.33334	15		120		27	
622	1.244972			57.33333	7.5		30	192.18	11	
623	1.156704			43.5		52.875	27.5		20.625	
624	0.9585072		64.5	30	7.5		24	163.35		
625	0.9888541		104.8					126.115		
626	1.323443		91.70667	120		100				
627	1.119064		86	50			15	60.06		
628	1.084249		43	50	15			125.12		
629	1.51402		129	50			52.5	71.73334		
630	1.194197		75.25	75			45	127.1167		
631	1.109744		101	50	22.5			118.61		
632	1.003698		143.44				20	92.085		50
633	0.9165771		43	60			90	90.08	30	
634	1.436354			170						
635	1.228647		96.75	50	8			145.635		100
636	1.296725			60			30	192.18	11.25	
637	1.329313			50	10	80		178.1667		
638	1.468913		52.4	62.5	18.875	60	80	84.08		50
639	1.462748		65.23333	50	17		72	64.055		
640	1.02875			50	32		20	288.27		
641	1.186792	149.12		137.5		90		123.365		
642	1.113646			46.66667	11.25	180		181.1675		
643	1.1407	1230.275		51.66667			15	60.06	34	
644	1.354265		86	50	15			72.07		
645	1.081778		64.83334	50	7.5			54.8		
646	1.257712		215	125			60			
647	1.292324		129	70	17		8	192.18		
648	1.583172		86	50	22.5	60		75.44		
649	1.331823		172	75	22.5			89.75333		
650	1.190255		43	100				360.33		100
651	1.135131		26.2	50			15	160.14		50
652	1.854972		64.5	50	30			75.07		
653	1.384374		129	100				96.09	30	
654	1.438041		172	100	15			40.535		
655	1.26541		201.25	50				45.04		

656	1.1431	703.01		50	15		30	216.2		
657	0.9911613			30			20			50
658	1.145803			42.5625	7.5			93.42333		
659	1.141909		172	45		30		82.825		
660	1.237071		86	50				96.09		
661	1.719977		43	50				100.09		
662	1.057086		129	60			45	96.09		
663	1.224327		129	41.66667	25		60	129.4533		100
664	0.7494786			37.5			30	72.065	15	
665	1.092613		86	50	7.5			70.565		
666	1.101616		43	50	7.5			141.13		
667	0.9061357		173	50			52.5	96.09		
668	0.9332009		82.915	50	11.25			96.09		
669	1.194444		49.55333	37.5	7.5			81.325		
670	1.202452		86	50				60.06		
671	0.9111301		344	100			50	333.6133		
672	1.323935									
673	1.618496									
674	1.284845		96.25					37.53		
675	0.8848614							122.12		
676	0.8798922	1406.03	200	27				80.07		
677	1.152506		34.6	75	9.5		30	130.555		100
678	1.072881			25	19			72.07		
679	0.7351514						30	33.33333		
680	1.124806		32.26	108	15		75	310.785	30	
681	1.261203		129					75.07		
682	1.498476	94.01	80.625					56.3		
683	1.094307		32.53	60				141.8825		
684	1.396973		193.5	50	15			64.06		
685	1.773058		193.5	50	22.5			96.09		
686	1.463683		64.5	50	15	60		128.12		
687	0.795462		42.65		18			44.54		
688	1.05646		39.3	100				127.6733		
689	1.084857		52.5	75			30			
690	1.267776	372.81	48.37	50			37.5	80.075		
691	1.166564		86.25	50			25	93.085		
692	0.9519647		150				20	101.595		
693	0.8540802		64.5	30			20	96.09		
694	1.059061			45.5	10		5	98.09333	100	
695	1.068274			43.33333	15		40	195.782	120	
696	1.237801		140	50	75			125.12		

697	1.144559		117.92	50	7.5			36.03		
698	1.121264		73.19	50	7.5			181.0017		
699	0.988102		65.6							
700	1.149537		43	50	12.5			222.205		
701	1.173889		98.26	26.66667				36.03		
702	1.152214		26.26	30	7.5					
703	1.63677		52.4							200
704	0.8841637		65.6	37.5			32.5			
705	0.8339794		32.76	30	5		23.33333	25.41037		
706	1.45328			50				36.03		
707	1.092717		86	100	35		21	263.58		
708	1.357528			75	15			62.05667		
709	1.199226			50	7.5	60		253.73		
710	1.39715		52.5	25	7.5			144.13		
711	1.153135		107.5	50			20	64.06	15	
712	1.791108		74.23334		37.5					
713	1.110049		28.6	135	15			125.12		
714	1.194293		86					168.155		
715	0.9377598		32.76	50				57.35333		
716	1.126902		166.2	50	12.5			240.22		
717	1.371362		86	75	22.5			132.12		
718	1.401328		71.67	105	5		30	102.8425		
719	0.951564		344	50	10			182.67		
720	1.080801		42.58	25	7.5	30		56.05		
721	1.239658		86	75				96.09		
722	1.023118		96.75			120		85.58		
723	1.293318		172					125.12		
724	1.601827		93.16666	301.6667	26.25		20	105.4333	15	
725	1.508513		172	200				150.14		
726	1.130749		26.2	50	26.25			225.215		
727	1.348143		43	70						
728	1.174928		78.6		7.5			68.61667		
729	0.9135064		48.38	27			20	96.09		
730	1.01911							90.08		
731	0.964394	232.3533	138.5					47.525		50
732	1.137873		215	125				215.2025		
733	1.090262		86	50			50	93.835		
734	1.005283			60			15	109.7667		
735	1.449523		107.5					75.07		
736	1.113277	200.455	98.26		18.75			73.57		
737	1.425085		85.3							

738	1.258729		131	55	15					
739	1.290168		172	50	15			48.04		
740	1.288224		72.1	75	40			77.0725		
741	0.668746		107.5	27						
742	0.7342175		64.5	54			40	168.155		
743	0.8939685		76.66666	27.5	15	30		80.07		
744	0.8417351							57.254		
745	1.241554		31.94	50			11.5	44.44444		
746	0.9701326		79.95	50	11.25		40	273.252		
747	1.036616		107.5	150			30		22.5	
748	1.232633		164.8333	90	22.5			45.04		45
749	1.368556		43	60	15			132.12		50
750	1.141203	351.51		64.95	7.5		40	101.4267		
751	1.091292									
752	1.230527		107.5	50			20	96.09	30	
753	1.042551	451.935		35	7.5			62.56		
754	1.499523		172			30		125.12		
755	1.032313		43					320.3		
756	1.253663		86	50	30			219.2		
757	1.461854		140	50	11.25		25	144.135		
758	1.489912		107.5	50			180	100.09		
759	1.071128		215	37.5	7.5			144.13		
760	1.0438		215	62.5	11.25		25	192.1775		
761	1.329783		43	50	22.5	238		62.56		
762	1.104876		139.6	50	22.5			192.18		
763	1.250926		86	175			60	52.54667		50
764	1.068023	351.51	52.4	72.5	11.25	60		56.305	15	
765	1.203167		215	100				120.11		50
766	1.155658		107.5	50				120.11		50
767	1.355691		430	150	22.5			45.04		
768	1.590515			50	7.5		20	100.09	15	
769	0.9250496		43							
770	1.05748		215	50	15			120.11		
771	0.7612244	351.51	129	60		30		322.2983		100
772	1.104399	351.51						65.06		50
773	0.5927359	351.51		50			90	96.09		50
774	0.8168269			60			60	192.18		
775	1.47295		86	100			80	75.07		
776	0.9459221	1220.83						75.07		
777	1.207554	144.62		125	15		7	82.07667		
778	1.235483		43	75	7.5	60		57.555		

779	1.281058		107.5			100		144.13		
780	1.504527	144.62		100	30					
781	1.424298							120.11		45
782	1.305326		107.5	50	15					
783	1.34732		75.25	50	15			75.07		
784	1.112224		86	100	30			144.13		
785	1.014241		215							
786	0.9362178		107.5	50	15			96.09		
787	1.101548			75	11.25			120.11		
788	1.269661	143.16	86	75	5		40	85.58		
789	0.9834469									
790	0.9255002	2109.04	43				55			
791	0.9053164	125.54		60				164.155		
792	1.332246		140		15		20	75.07		
793	1.469196			75	15			133.3333		
794	1.071348		64.5	50	15			96.09		
795	1.175194		86	50	13.83333			154.645	50	
796	1.222489		39.3	50	16			98.09333		50
797	1.050301		26.2	66.66666	15			183.5		
798	1.409062			55	15		90			
799	1.088724	703.01	70	37.5	15			112.1867		
800	1.025935		86			100		144.13		
801	1.240467		64.5	77.5				98.595		50
802	1.024083	281.34	64.5	50			30	60.055		
803	1.198503		86	50	7.5		25	64.16		50
804	1.549802		96.75	60						
805	1.216056		59	50	15			103.095		
806	1.10271		140	25	7.5			192.18		
807	1.221727		53.76					45.04		
808	1.277326		75.25	25						50
809	1.248074		68.25333	50				130.4533		
810	0.9355541			30			45	240.225		
811	1.335056			50	15		45	7.21	17	
812	1.208137		73.33334	50		120	30	56.1125		
813	1.046679		56.77333	31.25		30	15	73.565		
814	1.319275		258	83.33334	27.5		60	123.11		50
815	1.125631		43	170	15					
816	1.39312		32.76		17.5	90	15	56.055		
817	1.090441			50			40	120.11		
818	1.077736							96.09		
819	1.078175	47		50			40	177.66	30	

820	1.153242	703.01		36.66667			35.625	113.44		22.5
821	1.266412			61.66667			35.625		150	
822	1.335412			53.33333	30			88.08		
823	1.120719			30				100.76	90	
824	1.148401		86	30			45	60.545		
825	0.9387536	298.24	123.625	50	13.125			298.81		45
826	1.113771		71.25			30	20	56.74074		
827	1.017184			50			100	120.11		22.5
828	1.321192			83.33334	18.75		15	202.19		
829	1.250134	703.01								
830	1.080925			75	11.25			120.1125		
831	0.8866549		86	60				48.04	34	
832	1.091461			25						
833	0.8967681	1054.52		60	18.75			229.2133		
834	1.278304		32.8	33.33333		30	60	126.7833		
835	1.060555		103.9	50			60	198.1825	34	
836	1.132568	351.51	172	25	7.5	30		48.04	15	
837	1.266047	190.88	92.13	116.6667	24.37667			228.21		45
838	1.220117		46.5	55	7.5			72.07		
839	1.138729		86	100			20	150.135		
840	1.596402		201.565	75	22.5			100.09		
841	1.227271		94.73	41.66667	7.5		20	90.75		
842	1.019992		83.315					107.6		
843	1.285213							180.17		
844	1.026962	83.31	215	75	7.5			62.05667		
845	1.202689		215	150						
846	0.8972431		53.76	50	15			144.13		
847	0.9295455	149.12	75.25	50	15			207.69		
848	0.9199324		39.3	25			46.66667	126.62		
849	1.416318		43	50	15		80	100.09		
850	1.397828		86	55.9	7.5			288.265		
851	1.29625		107.5	50	7.5			96.09	70	
852	1.311891		65.5	50	37.5			75.07		
853	1.675884									50
854	0.9240522									
855	1.09121		86	50	7.5			62.56		
856	1.004665		153.375	57.5						
857	0.7796733		43					86.58		
858	1.107405			50	15			128.12		
859	1.259629		107.5	50	15					
860	0.9682629			62.5			20	103.345		

861	0.947216			40.8	12.5	70	11	139.932		
862	0.7743698		75.8	34.66667		41	15	175.5917		
863	1.455596			33		60	20	96.09	15	
864	1.329423		215			70				
865	1.166203		197.0867	50			30	96.09		
866	1.667009		215	100	15			45.04		
867	1.900007		107.5							4.5
868	1.516811		107.5					45.04		
869	1.009783		172	50	15			100.09		150
870	1.070233		107.5							
871	1.00586			100	15			75.07		50
872	1.146365			50	15			62.56		50
873	1.31696					100	40			50
874	1.190856		107.5	50	16.875			88.08		
875	1.608362		172	50	7.5					
876	1.45099			50				96.09		
877	1.084071		34.4	41.11	15			39.18667		
878	1.048231		129					72.07		
879	0.8691535		52.4					911.8478		
880	1.246412			33.33333	7.5	75	15	57.72		
881	1.065576	1054.52	102.88	50	9.375	45		112.1033		
882	1.37234		32.26	25	15	60		48.04		
883	1.126974			50				175.16		
884	1.023373		70	31.25			20	60.555		45
885	1.254523		66.58	58.33333	20.625			107.1978		
886	1.291017		86					192.18		150
887	1.530353		86	212	7.5			96.09		
888	1.946315	149.96		50	7.5			75.07		
889	1.232627		26	100			7.5	125.12		
890	0.9978549			50						100
891	1.203546		128.6667	34.375						40
892	1.533771		86	50	5			100.09		
893	1.585868		86	50	5					
894	1.281545	703.01	80.62	75	15	60		132.125		
895	1.436325		43	50	15	86.66666		72.07		
896	1.299594		52.4	100				144.13		
897	1.131375		86	100	20			100.09		
898	1.194429		48.1	80	7.5			88.08		
899	1.140629		86	47.5	11.25			48.37667		
900	1.205004			50	22.5			72.07		
901	1.212486	351.51	107.5	58.33333	15			59.305	120	

902	1.277595	351.51	107.5	66.66666	11.25			81.375	120	
903	1.269598		86.5	50	15	100		109.4333		
904	1.441641		86	50	7.5		60	40.04		
905	1.233034		172	50	15	30		64.06		
906	1.032744	1757.53	220	50	11.25			95.7575		
907	1.317607			40	15			86.58		
908	1.143262		86	50	15		30	80.07		
909	1.305776		52.4	50.5	15					
910	1.063554		88.43	40.5			15	154.64		
911	1.068785		88.43	27			15	103.0933		
912	1.259702		65.6	50	15			72.07		
913	1.355737	493.53	78.6	50			20	75.07		100
914	1.058308		53.42				90	75.07		
915	1.589638		86	50			30	144.13	48	
916	1.021176		39.3	55	30			144.13		
917	0.8619066	3515.07	64.5							
918	1.631377			50	15					
919	1.234176		19.66				60			
920	1.077405									
921	1.278095		96.75	82	28.125	120		582.205		
922	1.075459		43	50	5			72.07		
923	1.247375		42			60		237.22		21
924	1.088153		52.4				38.33333	237.22		
925	0.9180202			32.5	7.5		60	253.6833	30	12.5
926	1.187965			60.5	22.5	60	45	240.22	45	
927	0.9934146		33.85333	50				158.5714		
928	1.10273			25	15			33.33333		
929	1.185621	527.265				45		45.04		
930	1.367427		48.38	61	22.5	100	40	172.16		
931	1.366635		43	60	21.5		30	381.37		
932	1.479716		107.5	50						50
933	0.9249959		34.6	25	7.5	60		96.09		
934	1.084236	1446.2	131					108.6		
935	1.079677							25.02		
936	0.8424889	828.55	86		22.5			62.56		
937	0.9758546		64.5	110	22.5		20	163.352		
938	1.287525	713.06	53.75	116.6667	32		20	189.175	15	
939	1.372101		86	63.5				56.3		
940	1.299553			75	15			240.22		
941	1.495945			110.3333	30			240.22		
942	1.306035		172	100	7.5			180.17		

943	1.417908		129	157	30			72.07		
944	1.572382		26.2	50	16			112.605		
945	1.398246			50	15			72.07		
946	0.9268957			59			25	96.09		
947	0.9285716		82	60	7.5	30	12.5	312.292		
948	1.4253	403.71		100		60	80	45.04	30	
949	1.399789		39.2	50	15			147.14		
950	1.082866							72.07		
951	1.382086	300.68	74.35333					73.06333		50
952	1.09378		75.8	50			22.5	108.1		
953	1.27511		86	100	15			150.14		
954	1.08331		175	60	15			75.07		
955	1.011574		86				45	192.18		
956	0.9786904		43	50	3.75		32.5	96.09		
957	0.9287913			26.5	7.5		40	97.7575		50
958	1.156122		28.6	50	7.5			47.545		
959	1.252909		78.83334	50	15			120.11		
960	1.16489		81.20667	50	11.25			116.605		
961	1.281729			50				72.07		50
962	1.042215		86					180.17		
963	1.354444		86	50	15			151.39		
964	1.232347	103.27	95.83	38.5	5		25.5	170.4075		
965	0.9887418	2812.05	91.73	50	15		110	554.51		
966	1.227435			50	7.5		36		15	
967	1.299817	351.51		78	7.5	45		106.6		
968	1.454455	366.6767		50	29.25	120		62.555		
969	1.276013		53.75	50	15		60	37.535		
970	1.131031		86	35	7.5		30.75	58.8		40
971	1.171967		131	50				45.04		
972	1.535664			50						
973	1.518213		43	97.5	15			160.14		
974	1.419351		43	73.33334	15	60		77.32		15
975	1.011444	144.62	43					175.164		
976	1.123401		279.5					80.07		
977	0.9326853		56.5	50	15			148.135		50
978	1.119987		70.15	50	15			97.59		
979	1.197948		52.4	58.33333	18.75			94.58501		
980	1.086353		39	41.66667	31.875	30		136.928		
981	1.20764			50			50			
982	1.647089		86					72.07		
983	1.051048	1757.53	139.75	25	15		30	174.16		

984	1.198161		172					66.06		
985	0.8991843			30			80		30	
986	1.386983			50		60	70	96.09		
987	1.381459			50		60	15	186.13		
988	1.207694		86					150.14		50
989	1.130726		182.25	37.5	15			114.105		
990	1.221219		96.75	50				192.18		
991	1.063637		119.75	50	7.5		80	105.43		
992	1.294246		86	50	15			84.78223		
993	1.263196		86	50	7.5			75.07		50
994	1.411361	901.82	86					196.18		
995	1.04323		172	100	15	120		336.31		50
996	1.209219	143.16	86	50			30	74.07		
997	0.9504787		96.75	110				132.625		
998	0.7008134			42.5			20			
999	1.141201		86	75	13.75			120.445		50
1000	1.033448		43	27	7.5			192.18		
1001	1.305428			50						
1002	1.402646			50				240.22		
1003	1.581217		107.5	30				48.04		
1004	1.483816		236.5	50	22.5			32.53		
1005	1.390105		172	100	45			75.07		100

id	fruitjuices_portion	fruits_portion	industjuices_portion	leafyveg_portion	milk_portion	pasta_portion	pizza_portion	redmeat_portion	rice_portion	saltedsnacks_portion
1						110		25		50.24
2				30				52.5	50	155
3			318.28	20	99.01			169.2	125	
4			200	30				50	100	7.99
5		64		50	247.53				50	
6		88	240		124.27					
7			220					100	200	
8					151.61	220			159	30
9		86		32	99.01				181	
10		473				220		100	75	
11		40				400				
12		238.3333	480	35	124.065	208.8	100.17	110	127.5	
13				4.27	202.7633				77.5	109.98
14	240.28				185.645			100	100	
15									75	
16									75	
17	576.68	86			199.3967	160	211.75		129.1667	
18		55	224	30	41.25	120		150	155	
19		55	112	22.5		72		107.5	93	
20		172		27	128.92			140	187.5	
21		86	112	20	118.26			66.66666	200	
22								100	116.25	
23					247.53	301.88			38.76	
24		127.5	186.6667	30	123.76	110		140	186	
25				100	74.26	330		62.5	477	
26		126.5		60	123.7625	220		20	296	
27			288		111.39				283.96	
28		168.6667		50	222.78	100		299.4	174.6667	40
29		155			103.14			91.25	213.75	
30		225							103.3333	
31				20	108.6333	240.04			124	
32		86	353.3333		132.01			30	116.25	
33						320		120	200	
34				30	247.53			100	125	
35				16	247.53	196		220	25	
36		126.25		29	331.525	222.5			50	
37	240.28			30				17.5		39.93
38					148.52				77.5	
39				20	103.14	45			62.5	
40		200		30	207.12			135		

41	209.65	355		30	207.12			100		
42	600.89			100	393.525	330		62	125	
43		145.92		20				15	100	137.32
44	900.3	326.6667	301.9	60	50.745					
45		269.2857			541.7693			62.5	100	
46			533.3333		154.7	320		75	100	
47	217.5833			10	310.68					
48	164.84	150				99		15	108.5	
49		60	266.6667		154.7				93	
50						80		60	100.75	
51	240.38	98		20	148.515			75	69.75	
52		86				180			125	
53				40				23.33333	300	
54		86		20	165.02			35	265	
55		156		20					193.75	
56				39.5	314.05			60	100	
57				27	341.38			113.3333	209.375	
58		40		45				35	117.9167	
59		142.375			148.52					
60									298.125	
61						259.95		259.95	250	
62		145		40	182.305	40		40	62.33333	
63		159	344.5417	18	278.47			143.3333	146.5	
64		42			148.52		285.46		100	
65					74.26		249.28		125	
66			240		198.02		186.96		112.5	
67					99.01	63.33333			141.6667	
68		90			49.51	50	50.09		77.5	
69		251.17			26.82			14	87.5	
70		140						256	187.5	60
71		450		80	297.03				79.16666	
72			240		34.785			175	125	
73				70	297.03				100	
74				15	99.01				150	
75					77.35				250	
76		133		70			200.34	74.85	166.6667	
77		350				112		100	112.5	
78								140	100	
79		172	194.4444	45	143.355			100	142.815	79.95
80		67.83334			77.225	208.8		112	129.5	
81					99.01	79.6		40	198.5	

82		150		132	136.29	479.2		70	93	
83	503.165		513.31	40				150	200	
84					297.03				155	
85			312.66			112.5		47.875	142.75	
86	150.95		256.66	40					87.5	
87			520		206.27				187.5	
88					58.79			60	106.25	250
89			56		90.76	260		88.33334	200	
90			256.66		82.51	220		92.5	150	
91		115		27.5	123.76				125	
92		86		32				100	100	
93		150			230.51				312	19.98
94		107.5			132.0167			59.33333	71.5	
95		27.5	150.34	60	154.7			150	125	
96								95	279.1667	
97		133		29	103.14			60	50	
98		175	149.3333	35	318.2094			100	224.75	20
99		266			97.98			96	252.75	
100	240.36	80			117.575			98.33334	167.125	
101					64.46				62	
102		155		20	168.4533			50	62	
103	288.34	254.5			90.245		572.98	106.6667	241.3333	
104				67.5				154	194.3333	40.09
105				75	185.645			92.5	124.75	225.17
106		86	186.6667	23.5	185.645			115	208	
107				45	247.53			105	105	
108				20	77.35				62	
109					173.27					
110					128.92			60	212	
111		127.3333	84	30	128.92	100		86	142.0833	
112		40		17.5	113.235			95	97.375	
113									212	
114					247.53				106	
115		110.25	252.4	30	113.64	120	158.25		77.5	
116	314.48	155			272.2825		504.74	90	848	
117				100	148.52	500			424	
118		100		16	77.35				318	
119		110			128.92			12.5	106	
120	240.28			50				100	132.5	
121								72.33334	116.25	
122	240.21			51	154.7			100	311.9167	79.95

123								160	649.25	
124								160	299	31.96
125		86	287.7956	36	77.35		113.19	170	164.125	
126			256.6667		515.68			140	181	124.94
127			11.05	40	289.9667	25		65	115.5	
128	288.3		186.6667	38	140.27	320.02		100	93	
129				35	114.23			205	155.75	
130		571		15					106	
131		86		45	247.53			30	132.5	
132			480	45	123.76			270	159	
133		198		60	206.27			100	132.5	
134		90	155.5556	18.33333	173.27	250		183.3333	181.75	40
135		120.5		60	247.53			125	106	
136								100	106	
137			444.4445	45	206.27				106	
138	251.58			123	257.84				124	
139				50				190	50	
140			360	10		100		330	300	
141			240					93.33334		
142		135.5		45	124.7933			20	76.66666	
143		130			128.725			71.25	387	
144					154.71				318	
145	382.62	222.5		15	230.3367			80	356.75	
146		140	240	30	156.7667				365.3333	
147	142.24	80			88.7			70	130.5	49.92
148		107.5			117.575			100	116.25	
149		243.3333		40	128.92				318	
150			204.0778	20.3	272.28			50	96.75	
151		315							212	
152	144.23	82.25			134.9			60	100	
153	150.95	235	155.5556	15	56.47			125	312	647.32
154					123.76				106	60
155	240.36	187.5	153.99	22.71	160.89			55	49.875	
156					99.01		90.13	40.09		
157		520			96.95					
158		113.75		100			113.19	40	154.625	60
159		196.75		30	144.39				145.75	66.33
160	314.48	240						205	209	37.5
161			112	20	138.0925			55	112.3767	
162		170			206.27	191.9		76.25	170.6667	
163		130					176.91			

164	288.25		205.3333	29	162.44				111.71	
165		220	168		205.6275			100	257.5	
166								100	200	
167		86		40				80	371	42.62667
168						320				130.82
169		90			123.76			200	100	
170			186.6667	36	247.53		60.095		68.66666	68.74
171			380.42					150	318	
172		40				110	170.26			
173	169.57				70.39		125.29	100	69.5	
174		86			99.01			140	212	
175		86		40	49.51				132.5	
176		103.3333								
177			112	30	74.26	55		60	100	
178				50				30	450	
179				60	123.76				150	
180			240	10	23.21			90	158.875	
181		167.5			111.39	50		65	156.25	
182	240.38							85	131.25	
183			240	30	99.01			80	125	
184				30				100	187.5	104
185								20	124	
186		140						35	93	
187	480.72	446		60				68.5	424	
188			480		247.53		189.96	30		
189	250.39	50		35	546.62			65	155	
190					322.2222			70	200	
191	310.4825	110			242.71			50	105.315	
192								45	124	643.02
193	150.22	97.25		32	190.29			47.8125	132.5	
194	53.07	260		60	123.0333			100	129.82	
195	301.9	180	112	30				125	65.88	
196	240	113		60	247.53	80		150	310	
197								140	318	
198			186.6667						30	
199				40					62.5	
200		635		30				128.3333	150	
201						300			348.75	
202	75.48	135			154.7322			60	119.25	50
203		30		27	151.61	25		73.33334	123.6667	
204			252.6					125	159	

205						75			217.25	
206	251.58				24.75	320			212	
207			373.3333					80	318	
208		220		40	247.52					
209						320				
210		180			148.52				77.5	
211		84		30	105.2			200	158.875	60
212		84		30	113.864			137.5	158.875	60
213		171			99.01	260	192.31		77	29.48333
214				37.5	104.68			52.5	54.25	
215		110		30	151.61			100	169.25	
216		180	228	10.33333	137.17			65	457.125	
217					247.53			170	133.125	
218		88	186.6667	5	95.91667			50	125	
219				26.66667	129.2633			40	212	
220	240.28			36					400	
221	165.29	97.5			173.27			100	91.75	
222								80	31	
223					165.02			54	392.5	
224	176.92	101.25	137.5			330	249.28			
225		210			142.395			40		
226	142.24	165		10	49.51			81	81	
227	144.17	130		20		100		202.5	141.4375	
228				12.5	63.43	60		80	186	
229	120.16	85		30	247.53	75			98.33334	65
230		172	186.6667					80	116.25	
231			52.62		86.635				155.75	30
232			52.62		86.635				89.5	30
233		235			77.35			78.33334	320.5	
234	240.28	133.3333	112	30	74.515			95	116.25	
235	365.43	95	208.81		77.35		113.05		106	
236	367.085				247.53				62	
237		150.5				45		90	248	
238			224					60	132.5	
239		140	167.7489		198.02			50	185.5	
240		117.5	229.16	40				80	247.3333	
241	480.71	135			129.45				93	
242			149.3333	3.75		300		75	124.375	46
243		160		50	99.01			125.3333	392	
244	300.35	172	544.4445		360.18		100.17	123.3333	181	160.8133
245			186.6667		148.52			87.5	77.5	

246	167.72	190.625	131.56	79	114.31				79	
247						110		140	273.8333	
248		150			99.01	75			125	
249	243.47	246.5			198.025			43.33333	172.6667	
250		115	186.6667		82.51			80	232.5	
251	70.1	142.4		22.5	148.515			38.64	82.93333	20
252		135			58.79	110.92		86.66666	282.6667	
253		238.6667	253.61		156.0767	660		60	62	20
254	251.58	108		10				50	62	
255					371.295	67.6		50	116.25	
256	144.2	40								
257		172				110.01			265	
258	245.97	94.5		21.66667	159.86			20	105.6667	
259		87			229.3478	30		75	162.25	
260		151.25			63.17				149.75	
261			509.25		123.76	200.1			318	100
262	360.54				297.03			80.19	238.5	
263					201.11				93	
264		247.5	224	40	143.945			60	142.0833	
265		133		42.5	128.92			80	361.095	
266		86			122.39				100	
267				40	25			101.625	73.625	
268		90		30				35	93.75	
269		170.25		52.5			174.5		156.25	
270		120.5	252.6		247.53				50	
271		213	186.6667		185.64			145	250	
272		100		15	279.61			100	78.79333	
273		133		45	110.0133	254.4		120	150	
274		86			77.35			50	155	80.38
275		64			74.26			30	116.25	57.69
276		59.125		40	122.215			20	101.3333	
277		122			46.41			20	50	
278		137.5	137.5	40	992.2333			112.5	77.5	
279					47.32			200	62.5	125
280			151.56	20		220		75	77.5	
281		60		28.3	74.26				76.2	
282		135			116.025				124	
283		40	55.55556	25.3	99.01	82.06			50	
284	144.23								125	
285					247.53			18.75	93	
286	240.36				132.01	149.4		33.33	90.66666	

287	607.99	86			99.01		76.87			
288		86	240	20	99.01			50	106	
289		40			99.01			80	62	
290				15	74.26	80		120	100	
291		180		30	51.57			65	250	
292		99.91666			97.98	160		60	155	
293		86			101.4167	160		70	331.25	
294		86		135	195.96	200		125	187.5	
295		175			128.92			30	62	
296		172			115.51			100	103.75	
297					283.3675	110		160	103	
298		139.75	336	20				140	512	
299	144.21		560			85		77.5	85.25	
300		90			120.1525			42.5	125	
301	144.17			20	84.57	277.5		50	123.6667	
302			298.6667		123.76			180	187.5	
303			240	20		220		110	137.5	54
304					123.76			57.5	265	
305		40	137.3333					81.66666	212	
306				30	165			100	108.3333	
307			136.8889			400		84.44334	194.75	
308	288.46							20	120.6667	
309		40			371.29			350	116.25	
310	200.31					25		140	100	
311						190		175	50	
312								40	187.5	
313	1001.18				148.52				186	
314								120		
315	209.65	90	58.44445	4					93	
316	200.31	105	80.2	30	128.92				55.8	
317		184.5625		18		200		90	83.125	
318		113			200.295	208.8		90	50	
319		446	155.5556	22.65	74.26	162		24	120	
320			111.1111	300	103.14			11.395	55.8	
321		291.25		46.66667	150.985			60	118.1667	
322		171		30				80	193.75	
323		212.5		40	154.7			30	69.75	
324		400	151.56	60	116.03			31.25	116.25	
325		176.6667	131.56	20	386.76			31.25	135.625	
326					74.26			75	72.375	100.07
327	144.23	86			185.6467			90	143.75	

328	144.23	277.05		40	371.29			90	125	
329	75.48				99.01	159.375				
330						191.25				
331	144.13	200	112		111.84					
332				24	154.7			75	116.25	
333		205			74.26			40	77.5	
334	251.58	265				330				
335					330.04				125	
336		114.0667			135.9667			90	140	
337		193.6			166.05			100		
338		88.16666		15.33	94.88			62.5	93.33334	80.38
339								80	112.5	
340				64				93.33334	183.3333	
341	144.23	117.2		20	121.185			120	137	
342	144.41	93.6		10	98.495				120.125	
343			264.4444		165.02			166.6667	187	
344			186.6667	30	74.56				175	80
345		110	280	30	99.01			105	62	30.07
346		40		32	74.26	37.5			40.5	
347		135	252.6	33.33333	50.645			95	144.6667	
348		40	252.6	26.66667				77.5	121.4167	
349				30				150	530	
350			367.1111			500		60	212	
351	144.17						219.64		212	
352	181.77			24					106	
353		195	186.6667	38.75	127.885			100	117	
354		80			149.12			70	178	25.06
355			155.5556	40	116.03			65	262.5	
356				30	108.98	80		115	100	
357		86	112	54	222.78			120	477	
358			112	30				70	143.75	
359			186.6667		123.76	155			170	
360			149.3333		86.635	370			170	
361			373.3333		123.76	120		60	116.75	
362			311.1111			190	131.745	60	85	50
363				40	74.26			120	246.6667	
364						220				
365		136.6667						100	175.6667	
366	251.58							207.5	238.5	
367	240.38				146.97	327.2833		60	71.66666	
368		74.75		75	99.01				113	

369								25	424	
370			186.6667					100	106	
371		180	56		123.7625			65	98.815	
372		52.5		40		120		100	100	60
373		60	186.6667		99.01				131.5	40
374				30				100	106	
375		86	186.6667	30.66667	109.2533			100	97.16666	
376				100	92.825				75	
377		86			49.51	165				
378			280	59.33333	331.1266	185			137.8	
379	144.21	110			1144.889				75	
380	961.35		252		177.3967				530	
381	491.43	60			101.59	260		126.6267	100	
382		153		62.5				305	93	
383		86	112	42.5	154.7			52.5	41.5	
384							270.39	30		361.7
385		260			148.512					
386			112		99.01	440		175	100	
387	251.58			60		190		90	278.25	54
388			280		105.715			140	337	
389			174.2222	10	103.14	320		130	150	
390				60	74.26				159	
391		120		50	148.52				45	
392					103.56			50	69.75	
393				16	74.26				397.5	
394		30			71.68				224	
395	265.1667	40		20		269.6			96.88	37.17
396		86		20	148.09	330		20	310	
397				20			311.5	100	143.75	
398	240		186.6667	16				100	231	
399				35	74.26				312.5	
400		65		20	132.01	320			125	
401		166			298.25	110				
402	150.95	86		426	192.865	119.94		57.4	165.5	
403	251.58	135		45		373.8	429.74		530	
404			224	21	198.02			53.33333	200	
405	251.58				247.53					
406				30	189.77	50.01		71.66666	116.25	80.38
407	37.56	86			83.54	395			53	80.16499
408		256							200	49.35
409		86							100	62.015

410				75	135.4533			100	252.75	
411		243.3333		40	148.81			25	75	
412		324	254.63	200	345.505	304.4		85	125	
413					99.01				212	
414					99.01				424	
415	480.56		384.16	94.45	123.76				101	
416									200	120
417								172.5	530	
418						160				38.35
419		65.5		30				56	62.5	
420		116		20	66.01			20	125	
421		79		25	266.09			28	127.25	
422	251.58			10	99.00999			185.7	75	
423	240.38	110		82	125.83	225		129.5	106	80.38
424		570	112			40			46.5	
425			202.2222		247.53				200	
426				30	92.82				115	
427				20					201.5	31.96
428			368.37			411.84				
429		75			99.01			80	97.5	
430	240.38		186.6667	24	193.38	220			77.5	
431			240	22				80	165.625	
432		220		30	111.39	1100				
433		155		50	128.92			70	10.75	
434					78.385			100		
435	150.95			48	99.215			100	215	
436			112					60	129.5	
437	300.47		224	40	88.18			105	294.25	
438	503.17			50				132.5	250.75	
439		108		40	64.46			100	124	
440					123.76			20	62	
441				30				230	58.125	
442				20	99.01			120	212	70.13
443					222.775			90	92.75	70
444					247.53	330		340	269.875	
445			280		247.53			125	636	
446					99.01		400.69	280	200	
447					99.01		200.34	150	100	90
448					123.76	220		40	62	
449				30	148.52				265	
450	240.36	117.3333		13.5	113.45			10	146.69	

451		140				220			445	
452			112				76.87	60	112.5	
453				22				105	105.9167	
454					74.26				125	
455	288.34		112						150	
456									175	40
457		86	75						212	
458			112		74.26				100	
459		296.6667				320		30		
460	148.85	105			206.27				65.66666	
461			186.6667			220	300.51			
462						220	200.34			
463		214.6667	305.55	25	128.92			50	209	
464	251.58	160		65	128.92	200	200.34		210.75	
465		86	149.3333					40	31	
466		150								
467								118.5	170.5	
468				25					50	
469		172	186.6667	20.25	99.01				86.5	
470		172			119.8925			71.25	62.16667	
471				45	74.26				50	
472		167.3333	210.34	50	87.57	159.5				
473				25	46.41				112.5	
474		130		25	156.77	220		100	156	
475		140		12				40	106	
476					136.655	280		60	112.75	
477				20.25				20	124	
478	39.51	97.198		28.32667	103.56	67.5				
479		266.6667						160	62	
480		80	186.6667					130	250	50
481			149.3333	5		320	185.12	30		
482		206.4						60	187.5	
483			373.3333	105		82.5	240.35	45	530	
484			186.6667						150	
485			126.81		109.075	75		25	100	
486		40		10	290.7128			111.25	231.875	17.98
487		120		38	309.41	150		95	112.5	
488				27.5	46.41	150			232.5	
489		80			85.09				265	
490		220	108.8889		123.76				278.25	
491				40	99.01				186	49.75

492			186.6667				110.19	200	250	
493			388.8889					142	156.25	49.86
494			186.6667		247.53					
495	350.52		350	46.66667		360				
496	503.17			9	100.1967	100		100	93.33334	
497								40	125	
498			373.3333					150	100	
499				45	123.76			150	161.5	
500	480.71		303.12	40				40	157	
501				30				120	318	
502	144.21	300		32.5	76.32				280.6667	
503	144.21	45		20	163.295				114.8333	
504	450.53	215	186.6667	30	82.51	208.8			126	
505					99.01	304.4	622.98			
506			251.58			220				
507					74.26				31	
508	176.92							49	62	
509		86	186.6667	20		190				
510		178		10	198.02				89	
511		104.5	112	26.25	420.79				46.5	
512					119.3033	80		50	139.5	
513		40	112		99.42				50	
514									58	
515		77.5			554.6666			50	92.5	
516		56	254.63		577.7778			62.5	200	
517	188.69	262		118.625	149.12			94.8	108.5	
518			186.6667	16	23.21			600	243	40
519		149.5	153.99		297.04	90				
520	251.58	378		102				120	62	
521	251.58	350		65	198.02				93	
522			292.8445		244.09	200.97		175	400	
523			186.6667	30	264.03			15	125	
524			220.6483		107.6225	50		82.5	125	
525		140	169.1133	30	95.915				100	
526					309.41	144.1667		100	125	
527		85			77.35	220			155	
528		1080	186.6667					375	118.75	
529			112					60		
530				16	119.64	224		50	125	
531			777.7778			340		30	212.5	
532		147.5	112	60		280			175	

533				52.5	99.01		153.75		150	
534		220			116.5	518.77				
535								69.3		
536		130			217.4767				118.8333	
537		130		7.5	88.545	36		60	96.875	
538				41	118.055	125		80	69.75	
539		220		45	173.27	400.71		190		
540					165.02					
541		120			160.89				46.5	
542	108.16	190.8333		24	272.89	196.6667			193.75	
543	240.36	93.75		10.5	137.685	116.6667				
544		115			120.175			90		
545					48.735		95.14999	20		80.38
546		172	380.42	45				307.9733	99.125	
547			186.6667		370.2978			96.74	68.5	
548		200		30	253.715	165		100	104	
549		180	280	55				77.5	167.9167	
550								100	116.25	79.95
551	240.36	40		56.25				62.5	56.83333	
552		140.5	149.3333	45	302.5367			15	290.625	
553		40			123.76	86.4				
554	480.42	176.6667	373.3333	50	151.27				135.625	
555				45					375	
556		155		35		208.8			75	
557		276		140	82.51					84.57
558	150.95	162.5		80	129.45	880		96.875	625	
559		220		60	57.895	385		91.66666	232.5	
560		110.3333		25	221.225				95	
561		137.5		20	110.19				188	
562							219.64	200	100	
563	240.38		213.88	54			109.82	72	95	
564				23.2	116.03			96.66666	274.6667	
565	251.58				165.02					
566				55	198.025			112	172.25	
567		47			132.01				95.4	
568		310	314.9933	41.3	89.215			96.66666	232.5	
569		165		26	241.64			75	119.1667	
570		121.3333		25	141.81	193.52		80	171.125	
571		195			259.9	190			116.25	
572			253.61		116.8867					
573			311.1111	18	268.16			85	188.75	

574			112		24.75			100	93	
575	144.23	75.25			38.68	250		73.33334	139.5	
576	120.14	242.11		30	80.575				80	
577		40			38.68				125	
578		83		38	225.87			85	139.5	75.4
579	72.11			10	261.88	210		43	187.5	
580				0.35	111.385			66.66666	145.1	
581				45	130.465			90	139.5	
582	350.41	125	212.19	40.33333	99.01	190		215	93.75	42.98
583		399	140	32.5	132.01			72	132.5	41.24
584		86		20	248.54			20	77.5	
585	21.33	197.5		50	99.01			125	193.75	
586	144.06	180		26	149.125				151	
587		40			99.01			100	187.5	
588		40	186.6667		99.01			80	62.5	
589		600			515.68				125	
590	71.12			15	103.14				193.75	40
591		110	254.63	70	198.02	110			130.75	
592		340			198.025	100		11		
593			373.3333	40	74.26			42.5	266.6667	
594	153.71		149.3333	30	132.55		100.04	160	58.13	28.995
595	300.27	270		41.94	160.515			93.33334	79.5	
596		143.75				104		104.5	77.5	
597					123.76	72			150	
598			186.6667	67.5	123.76				156.25	
599		180	93.33333	18	123.76			125	88.75	
600		132.5		55	77.35			80	212	
601								52.5		
602					206.27					
603			112		222.77		249.28	66		
604		216				156.6		248.48	200	
605					99.01	160		100	62	
606						206.25	154.25	300	187.5	
607		110		143	101.7867	50	153.99	150	125	109.98
608			186.6667	10	123.76			100	125	
609			280						150	
610		155		40	248.54		100.17		73	
611					248.54				106	
612								80	46.5	
613								26.25	356	
614			224	20	99.01				31	

615	150.95	150		75	99.01				31	
616				35			400.69	82	112.5	
617		40						60	257.5	
618								30	31	
619		129.5833		80	143.685			22.5	357.75	
620		200		57.6	199.35	99			93	
621	200.18	80		35	396.04	179.775		75	252.75	
622			130.5	135		289.775	76.125	43	31	
623	240.38		343.42		310.68		78.02	93	93	
624			134.4444	40	168.315			40	79.5	
625		180		24	37.13	80		60	155	30
626				24	247.53			66.66666	118.8333	50
627		192.3333			185.64			20	212	
628		189.25			128.92			32	212	
629		172						96.25	100	
630		86						101.1	133.3333	
631		380		40	132.0133			105	158.5	
632		174.9		22.5	219.51			60	178	33.33333
633	209.65		213.88	60					106	
634			93.33333			330		56		
635				90	74.26			80	212	
636	150.95					330				
637				20	51.57			125	38	
638		182.5	373.3333	30	86.635			66.66666	192.3333	63.91
639		115	373.3333	20	121.3567			26.66667	155	49.93
640						99				
641		137.98			264.03			120		47.935
642		212.25	256.66		103.14			80	73.625	
643				80	186.41			221.5625	77.5	
644			137.5		148.52			100	125	
645				30	136.66	90			70.66666	
646	305.14			120	206.27			165	323.75	
647	245.98	450			198.02	125		78.66	93.75	50
648			186.6667	80	322.3617	85.21		106	604.68	
649	314.48		186.6667		198.6833	25		160	106.25	
650					185.645			90	265	
651				30	330.04			100	150	
652								80	112.5	
653			224	40	99.01			245	131.25	
654			186.6667		129.955			200	93	
655										

656				40	247.53			350	174.38	
657	480.76	270		31	247.53			180	159	
658					184.27	220		135	31	
659	240.21	110		32	126.605	100		70	143.5	87.31
660		135		30	99.01			150	212	
661					103.14			150	106	
662	209.65	175		110	99.42				150	
663	250.38	98		50	99.57	208.8			92.75	
664	240.38	173.75			99.01		100.17		125	
665		95			99.42				150	
666		150			99.42				200	
667		133.7			126.855			75	140	
668				24.66667				100	97.5	
669	131.03				55.86666			80	50.83333	
670				30	334.16	110			50	
671			503.17		258.265			55		
672		220			247.53	400				
673								35	218.75	
674					38.68				62.5	
675					160.895			80		45
676					165.02			15	500	
677	245.895	235		13.33333	148.52			100	112.5	
678			224	200	74.26			150		
679	200.24	115			309.405					
680				27.5				30	37.5	
681		64.25							110.8333	30
682								100	376.6667	
683		65		20	549.8222				38.76	
684	219.625	131.25			132.01				156.25	
685	288.34				99.01				218.75	
686	144.15			20	66.01				68.75	
687		86		56.66667	132.01			100	95.83334	
688		40		29	131.09	380		57.33333	93	
689				6.66	165.02			47	45.22	
690		86		24	115.51			69.33334	37.5	
691		86		17.5	99.01		200.34	22.5	59.375	
692					107.7			100	75	
693		148.6667		35	99.42				62.5	
694	144.27	113		33	235.0733	360	100.17	180	155	
695		52.5		28	187.19	320	200.34	225	387.5	
696		245		30	128.92			125	200	

697		206			259.91			30	23.25	
698		198		16	207.99		113.19	45	165	70.33
699		112.5	229.16	35	284.6567			160	93	
700		124		40	83.88667	270		125	76.25	10
701					123.7667				116.26	
702		55		20	198.83				62	
703					577.7778			80	124.5	
704					198.43			100	58.13	
705		96.875		20	222.775			80	89.375	
706				10	222.78	400		70		
707		202.3333			203.6925			150	150	
708					99.01	400				
709		180			112.93				187.5	23.97
710			168		148.52			41.75	75	
711		178	62.22222	20	165.015				125.875	
712		131	466.6667		247.53			86.66666	201.5	
713		130		146	128.92				106	
714		105		25.75				60	116.25	
715		146	210.5	90	226.9	80			79.5	
716		235	186.6667	130				70		
717	150.95							100	210	
718		40			88.18			65	148.6	
719				30					106	
720		170			99.01			40	69.75	
721				95	49.51			100	331.25	
722					63.43	400		32.5	84	
723					128.92				424	
724			186.6667		163.01			115	274.5	
725					77.35			300	212	
726		40			128.92				31	
727	219.07		448	10				56	106	
728		86		5	174.98			50	135.625	
729	215.7767	169.75			148.79			20	90.5	
730		119							93.75	
731		80		24.835					156.35	
732				6	128.92			102	100	
733	150.95	40						80	139.5	
734		180		20	198.02	60		200		
735			224		148.52			160	387.5	
736		180		27	74.26			100	116.25	
737				54.5	1155.556				47.5	

738		133.4		50.66667	503.7037				189.875	
739	144.23		168		99.01				137.3333	
740				34.5	198.5556			70	139.5	
741	125.19	260		60	248.54			30	200	
742	480.71	155		45	99.42			60	250	
743		140		66.25	132.0133			96.66666	135.4167	
744			252.6		37.13				112.5	
745					403.88				38.75	199.39
746		152.375			123.76			98.33334	124.5	
747	721.13		186.6667	60				150	125	
748	300.35	256.6667		40	198.025			147	141.625	
749		102.5		35.5	112.7633			40	188.5833	261.13
750					143.355			117.5		
751				50				805	62	
752				57	99.01			50	187.5	
753	360.57	100			221.355	260		592	62	160.75
754					128.92			200	318	
755					198.02			35	125	
756	572.8			20	77.35	120			212	
757		220	373.3333	27	99.01			100	212	79.95
758								150	187.5	
759				90	297.04				250	
760		135		25	132.0167	350		70	187.5	
761		180			194.17			270	125	
762					222.7725				128.23	
763		86.5		28.75	125.83				124	
764		125		45				100	93	
765					123.76				125	
766					123.76				62.5	
767				37		110		150	342.5	110
768			186.6667					150		
769									25	
770		150			123.76					
771				50		400			150	
772		450		60	148.52	110		60	106	
773					99.01					
774	240.36			40	99.01				125	
775			480	24	154.7			540	150	31.96
776						250		180	93	
777			186.6667		68.35	240		92.5		
778			151.56	20.25	80.25999			75	103	

779					148.52			120	200	
780								117.6667	106	
781			186.6667				204.7	150	125	
782					247.53			150	187.5	
783					77.35			200	154.5	
784					148.52			56.75	125	
785		515		37.33333					187.5	
786				32	99.01				125	
787		230			123.76	440	100.17			100
788		150		22.5	63.43	320		155	318	
789		100			207.12					
790	245.97	253		40	412.54			153.3333	200	
791		86		6	148.52			68.5		
792	190.22	51.66667			128.92			195	253.3333	
793	240.38	110		27	800	160		150		
794				40	99.01			100	99	
795		52.5		35	153.878			120	262	
796	144.21		56	180					205.9167	
797		141.6667		25	120.3267				93	
798		458.3333			278.47			120	465.12	
799					38.095			150	81	143.74
800	288.34			30	148.52			100	73	
801		110.5			131.2425			20	144.75	
802		143			86.635				93	
803	160.14	143		32	151.915			100	108.3333	
804			209.65				100.17	100	93	10
805		190		15	37.13		95.14999	80	116.25	
806								140	200	
807								140	100	
808	240.36		154.3	30	233.2725			60	196.625	
809	240.36	80	182.3	30	113.965	208.8		60	208	
810	71.12	155		10				45	81.375	97.5
811	157.24		317.02	10						84.57
812		92		100	110.0967				113.0833	
813		203		56	50.54				91.75	
814		120	112		117.5733			123	247.3333	
815	721.09	40		30.25	148.52	79.6		175	185.5	
816		405	171.1111	20	115.5133	75		30	75	100
817					123.76			192.5		
818		190			99.01				25	
819	314.48			129.0533			100.17	200	342.5	107.72

820		120								92
821		120		38.5	148.52			75	124	
822		180	220.1383		181.52	380		340	150	
823		75			211.61	406.6667		160		
824		262.5		27	123.7625	50		135	73.25	
825	292.77			110	248.0425			50	116.5	
826		92.83334		27.75	191.8325			180	50	
827	244.025	90			123.76			37.5		
828		172			396.04			120	125	482.26
829				60		561.6		15	155	
830		165		60	198.2267	100			96.5	
831		77.16666			99.01				106	
832	150.95			30	198.02	468		75		16
833							127.035	90	212	
834	251.58	195		30	148.515			63.75	100	
835		78.75		23.83	192.6175			225	168.75	
836		445			297.64			130	79.5	
837		296.4167			561.4814				123.69	
838		106.5		20	149.12			30	125	
839		265			103.14			98.33334	279	
840			178.6667	13	103.14			91.25	234	135
841		170			93.51334			25	186	
842		92.33334		25.33333	74.93			86.66666	92.875	
843		70		76	74.26			50	73	
844	240.21	285		50	99.01			10	137.5	
845	277.42			20	515.68			150	187.5	
846		455			149.12			80	125	
847		223			286.5133		90.13		62.5	
848	486.94				97.865					
849	384.57			32	148.52			113.3333	125	50
850								90	250	25
851					99.01		130.22		100	
852		200			77.35			112.5	248	
853									42.5	
854					173.7875				66.66	
855		306.25		60	193.38			7.5	50	80.38
856	229.6333	110	224		162.1825			50	194.7933	
857		758			113.965	60		80	100	
858	251.58			30	148.92	123.15		18	126.2933	
859								84	250	
860	75.12	215			193.38			10		29.95

861	240.33	154			227.415	82.5			125	
862	144.2	155				55			101.25	
863			233.3333	45	99.01	182		30		75
864	240.28			40	247.53			150	125	
865				24	99.01	275		131.6667	137.0325	
866		90	186.6667	50				105	187.5	
867		42	233.3333	23.13	577.7778			116.65	153.125	7.99
868		172						300	125	
869		125			495.05				112.5	
870								150	125	
871	560.84	87.5							424	
872	240.36	270			194.17			160	194.725	
873			256.66	16	247.53			100		
874			112	45	119.635				97.5	
875					247.53				100	
876				60	99.01			355	93.75	
877	240.36	302			248.135	220		180	38.76	
878				60	74.56			45	198.75	
879				60				17.815	79.5	
880			112	30	132.01		250.09		46.88	
881				62.5	148.515			130	239.065	100
882					99.01			200	159	100
883		133			193.38				62	
884		180							100	
885	251.58	86		63.33333	198.53	150			93	
886			88.88889					40	159	31.96
887		86	233.3333		99.01				212	
888		180		68.4	77.35			295	106	
889	251.58			20	128.92			105	93	
890								45		
891	240.38	60		90		150		80.16	166.6667	
892			112	10	103.14	300		20	212	
893			224	10	148.52	300		35	212	
894				28	37.13			155.5	115	
895				34.5	74.26			48	108.125	
896								60	62	
897		207.5			103.14	400		100	212	
898			140	80	169.145			100	147.6667	
899	209.65		112	30	112.415		77.12	180	166.3333	
900				15	74.26				50	
901					136.91	169.8			132.5	

902					118.295				90.66666	
903					231.02	110		96	77.33334	
904					64.46		270.39	60	100	
905		266	253.61		132.01		95.14999	80	112.5	
906					105.885	100		40	206.25	
907					231.02			164	75	
908	628.96	140		54.83333	206.61	206.65		50	116.25	
909				30	297.64				124	
910				40	149.0325			10	124	
911				60	149.0325			10	139.5	
912			112	20	74.26			100	116.25	
913	232.715			20	77.35			140	120.1	116.53
914		220		40	148.52				100	
915		146		9					119.25	20
916		430	355.055	20				90	124	
917				30			249.28	104	75	49.975
918										
919		180	254.63					45	116.25	94.78
920					247.53					40.98
921		150		100	202.1433			125	243.75	
922		560		60	216			60	100	
923		40			194.17			21	21	
924		95		16.66				25	62	
925	366.89	60		10.27667	93.22	224.77		64.96	96	10
926		55		60					212	
927		100.6667		37.5	117.575		130.22	37.5	176.375	
928	251.58	105			473.3333	67.5				
929		184.4		24		167.8667		80		120.565
930		40	93.33333	60	132.01			185	187.5	154.515
931		90			436.8933			120	185.5	40
932		180		20	706.6666			56	105	
933	169.2167	110.3333		22.5	99.21333	85		47.5	82.66666	
934				15		220	414.6	95	185	
935	240.38	40		31.66667		110	276.4	90	92.5	
936		263		24	193.38			75	137	
937				86.5	247.53	340			106	
938	144.23			87.5	309.41	144.4		100	248.75	90
939			67.47	50	96.69	221.85		123.75	150	
940	240.38				144	183.3333				
941	240.38				247.53	346.6667		73.33334		
942	240.28	338		30	185.64			150	240	

943		250	186.6667	25.875	148.52			100	219.5	
944								300	93	
945			168	40	111.69	340		100		
946	243.47	95		56	99.215			500	57.94	
947		175		37.5		112.5		75.75	118.8333	
948								60		78.225
949		100			51.57			116.6667	137.3333	
950				16	74.26	220		80		
951			112	49	38.68			150	162.7933	
952		103.2	373.3333		148.5133			135	103	
953				8				100	125	
954		130		20	466.02			250	62.5	
955		180							116.25	
956		450		90	99.01				150	
957	144.17	110		39.6				20	46.5	
958				80					62.5	
959			253.61		206.27			80	196.5833	
960	144.23				49.51			46.66667	210.0033	
961				15	37.13				150	
962				43	74.26				636	50
963				24	193.38			250	375	
964	120.125	35	84.065	30	99.01	12.5		55	134.375	
965				10	37.13			123	125	31.96
966	132.1275			15	198.025	166.25		77.5	132.5	
967	244.32	150		25			149.57	90	153	69.96
968	263.9167	105	373.3333	40	311.99		124.64	125	161.5	140.4133
969		110	254.63	12	138.715			112.5	156	
970	300.41	188			196.22	120		48.33333	96.25	15.935
971	366.89		186.6667					120	150	35
972										
973		130	186.6667	25	165.02	110		99.94	106	10.11
974				27	117.575	80	106.665	70	112.5	
975		510	252.6	7	176.36			150.5	108.125	79.95
976		85.33334		36.66667		400		200	200	
977		64.5		30	143.0167				84.25	
978	167.72	89.875		28.33333				34.2	378.42	
979	144.21	85.5		51	149.12			160	170.5	
980		142.5		50	102.105			45	68.75	
981			373.3333		495.05	29.7				80
982								170	73	
983		141.25				250	100.17	101.25	175	

984		86.66666			281.565	250		111.5	100	
985		492.75		22.5	198.02				146	
986			140	27	99.01				132.5	
987		170	186.6667	27	99.01				37.5	
988		86		16					100	
989					259.9	100			106	
990						180			115.5	
991		106.5		133.5	85.60667	110			116.6667	
992				20	188.225				178.875	
993		40	253.61		348.09				397.5	
994				50				240	168.75	401.89
995	240.36			200	49.51				150	
996	240.28			23	72			55	334.3333	
997	480.56	170.6667		26.66667	128.92			112.5	103	
998	598.46	258		130					77.5	
999		76.66666		37.5	143.0613				145.6667	
1000			186.6667						62	
1001		301.8								
1002		316				208.8				
1003		258		21	132.0133	80		30	212	
1004	240.38			45.65		79.6		50.66667	200	
1005				30		330		30	180	

id	softdrinks_portion	soups_portion	sugar_portion	sweets_portion	toast_portion	tubers_portion	vegetables_portion	whitemeat_portion
1	249.96		2		16	25		100
2	302.04					90	30	
3			10					
4			6.25	60	31	30	20	20
5			2.5		48			140
6			24	45		270	30	39.6
7			8		80			55
8	149.98		10.6		32			40
9	148.1175		4.533333					180
10	249.96						50	160
11	249.14		5	200				
12					27.33333	157.5		
13	246.1733		8	60	40		10.66	200
14			12					
15	374.94		5		15			35
16	249.15		5					140
17	298.98		22.325			134	20	100
18		649.99	4		30	30	15.725	280
19	249.96	60			30	85	31.45	140
20		563.3233	10.325	66				
21	207.62		5.4	63	2.75		30	45
22	415.25							
23	208.3			77.45	40		14.15	
24		20				140		
25			4.5375	107.605				
26			9	14.58	32			
27							100	680
28	144.13		5	40		35	52.5	65
29	129.76		6.5	57.06		40	80	
30	149.98		5.616667	105	40			76.5
31	74.99		3		26.66667		100	43.33333
32			7.466667		55		18.75	140
33			57.6					
34			3.75				60	100
35				52.5			28.5	60
36		325	14.66667	11.2	46.33333	90		
37	124.57					177.53	87	
38	124.57				12.6	25	20	
39					24			
40			8	40	16		51	

41		22					60	
42			3.75			260	360	63
43	363.34						26.06667	
44							142.5	
45				25		62.5		
46			12					122
47			4		118.5		10	
48	124.57			160.49	14.81			180
49			17	20			80	180
50	130.19		4.815		32		77.76667	40
51		162.5			16			70
52	342.58			22				240
53			10				120	
54							30	
55			10	400			49	
56	156.23		8			105		
57	249.96		15			109.94		
58	350.32		3.666667		30		60	106.25
59					35		110	80
60			24		48	280		237.5
61						173.25	173.25	
62			3.5		24	42.6	20	40
63			16		15		45	
64	149.98		7.2					180
65	1046.43		4			45		310
66			4					500
67	174.9733		4.8					40
68	124.98				48			47.5
69	298.98		16.8	75			218.7	195
70			8.8		11.85	300	120	120
71	498.3		10.8		47.16		630	101.6667
72	249.15			100				270
73			4		32			
74			32				15	
75	149.98		7.5				144	60
76	498.84		4.8				31.5	360
77	274.96		6	59				
78					30	141	60	
79	158.31		12		39	45	24	
80	373.7233	88.255			30		59.125	
81								

82								
83		135.49					20	
84		519.99					45	40
85			7.2					120
86			11		30	195	78.75	127.5
87						60	50	260
88		519.99				60	112	105
89	249.15		3.1				40	
90	249.15		2				40	
91			12				30	75
92								120
93	433.265	234	7.85	31.72333	65			40
94			12			310		120
95			8				75	
96			5.1575				42	
97			7	75				
98			1.25				110	120
99			40.65				100	
100	124.57			40	23.25			
101								135
102			12.33333		14.66667		37.5	
103	149.98		9.85	186.25			72.75	640
104			6.8			400	372	180
105	603.56		5	70		145	75	155
106			8			70	75.66666	140
107								
108			6.25		27.6		30	40
109	83.32	433.33			30			
110			20.74					30
111			21.5	75	24		83.33334	46.66667
112							22.5	70
113	499.92			30				
114	249.96						62.5	
115		519.99						
116			7.5	40	80		37.2	200
117	1494.9		18		80		75	100
118	399.94		9.225	17.5	16		80	90
119			7.25		24			
120	240		4				72	
121	499.92		2.375	37.5			20	
122	363.34		9	80			39.33333	52

123	1388.667							
124			12.76429					180
125			8.95	140.375	24		58	
126			18.25					
127		22	10.8					80
128	249.96		12.8		32			
129	524.915		7.45					
130							25	10
131	208.3		4	72.91			48.75	402.5
132			12				50	500
133						1065	50	
134	640.86		2.4				32	175
135				160		710	45	
136	207.62		4	53.32			50	
137			4		75		450	360
138	249.96						150	360
139							100	
140	312.45			50			30	
141				75				
142	149.49		3.6		16	75		48.75
143			3.733333		16			
144			15		32			60
145			12.75				12	30
146		300	5.133333			300	140	238.75
147		103.38	6.295		15	215	100	450
148			2.666667			100	26.66667	65
149			16.25	240	16		49.5	80
150	228.725		9.17	103	44		95	60
151			3.7		39.66667		50	70
152			11.64	30.306	33.53333		53	60
153	312.45		6.06	90	31.5	60	50	
154			12	21			52.5	
155		40			14	45	18.12667	30
156	149.49		9.030001	41.5		280	54.6	
157			2.5		120			
158	374.535		6	238.75			56	
159	120.11			10.4			57	
160	246.55			87.5		300		
161	134.955		5.75		24	155.2	70	30
162	312.45		6.675				75	30
163	299.955							

164			6.8	26.25	64		100	84.5
165	187.47		12.6		31		40	200
166	249.96		4	180				
167	312.11		3.675			45	26.25	
168	324.95		3.675		188			
169	249.96							
170					27.6		70	78.33334
171	363.34							
172	249.15		6					
173	141.6467		11.85	60	20			
174						20		
175				14.98			90	
176		150.16	24		56			
177			3.6				45	
178	299.95		24				45	120
179	124.98		12					40
180			8		20		19.5	60
181					15			40
182	156.23		6.6					
183			4				50	140
184	156.23		3.6	40.08				
185		325	2.5625					
186	149.49		0.8					
187			7.5					
188			21.6					
189	363.34		12		45		43.5	
190	228.79			37.5				180
191			13.54		16		40	
192	314.99		2.59		160		78	
193			5.333333				105.9	120
194			4				105	
195			4				315	
196					20		100	
197	224.235	519.99	7.2		80			
198			9.6	120.17			5	
199		324.99	8				30	
200	374.695		4.165		32		12.9	120
201			6.6					126.155
202		212.875	23.25			50.975		
203			3.166667				61.5	60
204			9.75					90

205			6					80
206	374.94		6.48					320
207			7.2					
208	149.98					175		90
209			4.6					
210				160			60	195
211			18		20		50	
212					14.63333		50	
213	209.9767			60	10		40	70
214			8.1			16.5	37.5	70
215			6					
216			34.66667		40		45	180
217	560.585		10.01		21	95		100
218			10	127.085	16		11.25	
219			5		12		30	100
220	249.555		12	22			90	
221	374.13		6.816667	28.75				
222			16	60	80	235	35	
223	999.84		4		40			240
224			4		25			35
225			10			355		
226			6.15				215.35	70
227			7.35		21.66667		50	80
228			5.8125				40	
229					31	60	50	40
230			3.75					150
231	149.98							
232	149.98							
233			7.3		24			240
234			10.225		24	42.6	60	120
235			16.625	30	17.4			
236			12	77.33				92.31
237			2					
238			57.19					
239	249.96		6.333333		30		50	
240	312.45						70	213.3333
241			2.25		24		100	
242	249.96		4.533333	22.5	25			120
243			9.6	150	20	130	50	
244	520.75		48.6	53.75	39			80
245			24			290		

246		260	9		13	47	56.66667	110
247	833.2		10.325					32
248	281.205		10.85		24.33333	15	10.625	
249								
250			8	80				
251			10.575	50			49.06667	68
252	162.07		3.834286	12.53333	38	114.6667	75	
253			5.25	80	35			110
254	363.34		4				10	75
255		248.51	5.666667	50		41.55	41.55	
256		259.86	43.5					
257			9.3				50	
258			8		32		51.66667	50
259			5.333333		20.625		75	100
260					20		14.732	116.6667
261	249.96		12	310			108	
262	249.15		14.25	12.5			30	132.5
263	289.015		15				75	30
264		520.01						37.5
265			21.6					
266	249.96		5.7	33.75			64	
267	240.22		4.63		25		30	
268	146.99						50	
269			4.5		15.8			
270								
271	999.84		12				200	
272	279.31				14		24.25	120
273	149.4867		6			140	43.33333	
274			12					
275			3.6			8.75	6.25	40
276			4.625		16	64.58334	42.41667	160
277			4	15		8.75	5.625	140
278							30	
279	200.18							150
280			2.25					100
281	149.49						76.6	55
282					32		67	
283			4.8	15	15		35.8	155
284			5.6				50	50
285	363.34					22.5	24	
286	249.15		12		32	20	100	

287		519.99						
288			6		25			
289	364.53		6			50	15	
290			63					
291			7.5				50	
292								100
293			5.166667					120
294			13.66667					10
295			6					
296	282.64		8	116.6667	60			
297		520	8.55		45	80		
298		1040	14.5	483.26	113.3333	290	60	
299			29.4			77.99		
300			5.625		40			
301	145.81		8.2			30		60
302								110
303	373.725		4	3			30	
304	749.88		6					
305			5.4		14.75			
306			8.82		31.5		10	40
307				195.0467				48.75
308			8.666667		39			50
309			6		100			
310	499.92		8.125					
311	299.95		5					
312			5					
313			36					100
314			2.25					
315		520.35					24	40
316		520.35	5				13.5	40
317			6				35	85.50667
318		312			10	125	57.5	
319	364.53				50.4		34	
320			5				18.1975	180
321							90	105
322			16				100	170
323	130.19	519.99	7.5				25	
324			7.5		28			
325					26			
326	249.42		16			70		40
327		454.99	8	30	17	90	44.5	

328		454.99	9	16.25	30		33.125	
329			8.325001	110				
330			7.5	285				
331				200	12.9		50	
332	78.11		12				30	
333							3	65
334	240.22							102
335			4					360
336		659.11	13.41667				12	
337			4				120	
338	207.62		9.333333				23.125	50
339	183.3067		3.675					
340	162.35		9.6	25	33.6			80
341			15.1			110	57.33333	300
342			7.688		46		66.6	55
343			14		150	80		65
344	299.95		10.4			80		200
345			23.4	12	23	79.89	21	
346	156.22		3.45			63.9		18.75
347	249.96		3.7	49.825	22.5		30	40
348	114.045		3.375		30		30	40
349	1868.61							
350			23.6					
351			14.4					
352	325.99		9.075				30	
353							50	100
354			4.395			110	20	
355	249.15		10.5					116.25
356	249.15		6.666667					
357			10.8					
358			5.475					200
359	274.065		4.6125	23.48				
360	218.31		4.575	35.23				132.5
361	374.13		6.625	79.66666				65
362			24	117.375	75			
363	149.49		5.37				90	
364	149.49		8					
365		100.5	8.4					
366	244.98		8.625				200	
367	112.48		5.692857	107.79				
368			7		16		37.5	

369	334.465		18			30	90	50
370			6.625					180
371			6		24		85.5	
372	467.155			83.66666			60	
373			4.8			15	13.33	95
374			21.6			141		
375			13.5		18.5		30	100
376			3					
377			4.8		15		60	
378	149.49		11.05	70			43.88	90
379			11.58				35	
380			15.8	22.5	40	53.75	83.33334	140
381	299.95		10.61667			188	90	
382			7.866667	22.46	48			
383			5.852	79.13	34			
384	570.965			25				
385		292.5			16.25			
386			11.5		15			
387			5				30	200
388			10.25					
389			12.13333				52.5	140
390	74.74		3.666667					
391	100.09						20	
392			9.6				120	80
393	149.98						75	
394			6.15		20			73.75
395	363.34		16.8				94	65
396	149.49			34.24			35	
397	187.23		3.283333		20		132	180
398	249.96		12				71.66666	
399							35.83333	
400								
401							25	
402			16.2		24			
403	761.365		12		200			40
404			32			35.7	63.75	
405	624.9	519.99		30				
406	215.815	519.99	7.85	25.45	34.25		30	
407	156.23		8	25	20		30	120
408			12		20		200	
409	249.15		12	25			50	

410			13.13333				45	60
411	519.06		7.1					
412				238.96			53	
413								160
414								160
415	374.94		8.475	16			104	80
416			5					310
417	373.725		2					121.6667
418	224.725							60
419			9.6	150	20	20	50	20
420			5.6					
421					18		178.6667	180
422					35	10	32.5	80
423	129.975		12.4	20				105
424	124.98		2.5	17.5			272	70
425			25.125		40		115	
426	83.32		9		35	280	72	48.75
427	181.67		10.125					120
428				30				
429			7					80
430			14				120	100
431	249.15		27.2				45	217.13
432				37.45			103.75	
433			12.5		32		60	
434	498.3		24	7.5				
435	298.98		9	92.18			50	
436		89.47	37.8		43.75			
437					10		45	81.16666
438			8				90	
439			6.25				22.5	50
440			6					
441			7.125				82.5	
442	149.49		3.6	143.98			60	
443	224.235			100				
444	1041.5		11.33333		80	141		120
445			30					
446			4.8	40				
447	285.82			80				
448			12		32			
449	299.95		2.25				60	150
450			9.833333		32		121.25	146.25

451	149.98		3.375	37.5				50
452	149.49		14.25					
453	149.98		39.9				21	114.615
454			12					80
455			12					40
456			36					80
457			5.475					40
458								55
459	149.98		3.75		24	22.5		
460		360	4			92		40
461	498.3						50	
462	430.82							
463							47.5	120
464						210	50	137.31
465		160			30			
466					10			
467	149.98		14.4			5.5		
468			10				100	
469			9.6				40	
470			5.7			20		
471	373.725		7.2		20		100	
472		195	7.08				2.7	
473			4	22.5		88.88		40
474	499.92		16.53333	99.97	25		66	65
475	75		14.13333	99.97	5.2			65
476			8.133333	280	64			40
477			2.5				46	
478				32	20		128.3333	44
479	199.97		13.6		25		52	
480			5					
481			6.885				15	
482			8					
483			2.7				30	
484			6.25	50		160		
485	130.19				16		160	56.875
486	187.54			179.22		37.5	46.875	
487	249.96		2.25	40			100	65
488			8				15	180
489			8.25					85
490			19		25			140
491			6.4					65

492	249.15		9.6					
493				15	151.6667	36		
494			12.5		60		100	
495	998.215		24				133.3333	80
496	124.98				26.25			162.5
497			5					
498			9					
499	387.44	341.25	5	50			120	65
500	208.3		10.5	22.5	20		400	95
501			3.75		15		50	
502	149.98		7.5		24		97.5	223.3333
503	149.98		8.25		30		36.66667	38.75
504			27.6	43.75			45	
505	499.92		24					
506			9.6					80
507			7.2				60	70
508		52.8	4	25		21		
509			2.25		30		30	
510			9.6		39.25			105.625
511					15.5		75	120
512			13.45		20		21.66667	
513						140		100
514	240.22		2.25			280		67.5
515		119.76	49		10		23.75	
516				48	18.325		137.5	
517		260		75	10		138.95	
518			26.75				31.33333	60
519			11					
520			6	80		140	200	
521					8		50	
522			12.75					
523			4				60	
524			6	130	10	90	592.5	130
525		260	3.6					40
526	810.95				52	20	15	52.5
527	293.49		7.5					105
528								
529		779.99	3.533333					
530	124.57		5.8	28	40			48
531	249.96		9	43	50	126	25.75	
532			6			186.7	45.33333	220

533	149.49			40				
534	149.98							
535			2			69.3	69.3	
536			4				40	
537		349.99	10					155
538	207.62	260	3		6		258	48.75
539							31	
540		390				50		
541	150.935	519.99	5.2				60	
542		74.83	3.133333	48.6475	30	75	13.8	56.25
543			12			110	23.33333	24
544		520						
545			3.375	50	27		20	
546	499.11		2.25			90	30	150
547	499.92		12.66667			90		30
548			9.6	59.88			45	
549		72		100	25		30	95.44
550				124.45				
551				126.38			10	32.5
552		519.99		32.5		81.95	75	51.75
553		519.99	12	10				275
554			10.85	123	18.33333		45	55
555						30	240	105
556			9			30	180	105
557			12				55	
558					25		47.5	
559	112.48						33.75	
560			9.333333	60.005		40	300	73.33334
561			4.725	192	18		52	102.31
562	249.15							
563	207.62							
564			8.75	30		180		
565			12					
566							133.3333	74
567	149.98		9.6					120
568			9.85	152.17			66.6	
569	259.53		7	5	21.33333	346.25	72	40
570			8	60	27	400		140
571	149.98		8	56	24	50	30	
572		390	18		30		15	
573			48				100	40

574			15.6					
575			6.512					
576			7.105		20		45	52.5
577			3.19		32			75
578			5.966667				43.61	
579	622.87		2	10			30	40
580			9.75	100		140	45	
581			21.9	20			272.5	
582	363.34		15	150			242.25	120
583			23.34				15	
584				90		33.75	30	
585		1040			24		100	
586		520	7.2				87.5	100
587	747.45		16.5	90				
588			36	20	10.3		24	
589	306.84			50			63	100
590	311.44		12		30	140	90	90
591			8	80	40		50	40
592				68				40
593	799.35		3.3				45	153.3333
594			9.6	61.335				
595			15		17.77		27.5	
596				30		174	104	21
597	130.19			54.5		22.5	18.75	180
598	249.15		11.33333					
599			12		15		68	
600			5.75				45	40
601			11.95					
602	519.06		5					
603	298.98		9	17.75				
604								
605			8	30	44.1			
606			5			45	37.5	
607	249.96		3.375	16	40.33333		17.5	
608			7.125				120	127.5
609		24.9				30		40
610								100
611			8		25			272
612							33.3	
613	298.98		9.6					67.5
614			5.925	80				

615	49.83	1040		334.33	10	280		50.59
616	364.53		3.8	100			55	
617			9.6	421.25		480	200	110
618	149.98			168.5		160	87.5	70
619			10		25.87667			260
620		211.575	3	6.79			54	66.66666
621	364.53		12	101.97			30	120
622			8	95			122.5	40
623				47.5				
624			6		30		95	
625			7.2		2.96			
626	149.49		9	120.37			50	
627			12					
628			12.5	102.23				
629	249.15		3.583333	48		11.6	47.73333	
630	769.02		8.4	43.5		20	50	
631	249.96		2.25		15	140		91.5
632			3.68			105	21.66667	65
633			4.5				58.5	
634	249.96			45				
635								
636				80				
637		520				42		270
638	249.96			26	31	60	90	
639			18	122		90	30	
640			19.2		50			
641			3		30			184.62
642		60.05	10	50			100	30
643	178.555		3	50		100	100	68.75
644			3.6					
645		325			10		66.66666	40
646	311.44		13	80	40		277.5	110
647	312.45		19	91	30		78.75	
648	622.87		5.174	14.5	49			
649			15.3625		50	280		280
650				10				
651							60	
652			3.75		100			
653			4					
654			4		30			
655			2.25					60

656		520	10.8	17.36	40		30	
657		520	24	113.61	32		108.5	
658			4	80				30
659	78.11			50			49.16667	100
660			4.8					
661			5					
662					15		45	48.5
663		780.38	10.425	44			50	80
664	408.27	422.49	3.733333	21.5		55		55
665						70		40
666						52.5		40
667	207.62				35		255	
668	386.425		7.733333				327	90.625
669			2.72				37.5	60
670	249.96		3		75		50	60
671								
672	103.81							99
673								
674			6					120
675	363.34		7.2				72	
676			4					85
677			16.4			96		200
678		520	24					
679					45		236.6667	32.5
680	520.75						69	155.6333
681			5	96.69	48			40
682			3.75					168.3333
683			15.9				16	70
684			6			20.625	17.185	120
685			9.2			22.5	18.75	120
686			5.2			11.25	24.375	240
687	211.08	1039.99	5		24		51	40
688	144.13	260.17		100			258.4	
689	333.28				30			
690			6.9		13.66667		117.3333	
691			4.4	10			52.5	
692			3.375		10	80	19	
693		520						100
694	350.32				32	62.975	15	40
695	189.035	649.99	24.15		39	110.44	25	130
696	249.96			22				

697	78.11		9.6				26.66667	
698			55.925	20.04			37.5	113.75
699	26.04						175	80
700	130.19		9.066667	50	39		71.04333	
701			3.6					120
702					16			170
703	499.92		12		32			
704		406.17					5.4	
705		454.895			8		11.25	
706			3					
707								
708			5.75				80	
709	149.98							52.5
710			7.2		20	13.5	20	
711			6.8			135	15	97.5
712				120	67.5			
713	249.96							100
714					40		100	134.375
715			5.6				64	14
716			5.333333				50	
717	74.99		6.6					
718			39			62.5	58.42	
719			9.125				36	
720							35	30
721			4.8			140		
722		376.28	6.15			30		
723		643.17	25					500
724			8.55	20		90	50	
725	149.49		16.5	57.06	48			100
726					26			30
727								
728			4.466667	26.88333			100	36
729			4.8					195
730			4					81.25
731	280.29		2.25				50	110
732			13.08333				10	
733						120		
734	437.43		7.125			105	87.5	
735								
736			12		25		60	
737			4	10	21		300	100

738				20		200	52.5	60
739			4.8					83.83334
740			7.216667				57.5	
741	249.96	519.99				35	30	
742	124.57	325	10.8			60	30	
743	278.31			22.5	22.66667		140	65
744	282.37	325	2.775	3.75	16			
745	150.14							
746					20	45	62.5	170
747	249.15		36				30	
748	249.96		7.125	143.72			50	177
749	149.49		12.25	143.72			88.75	122.5
750	339.985		4.666667	120	68.14			
751	2325.39			30			111	80
752					15			
753				22			40	100
754			9					
755	149.98			205.32	48		40	48.75
756			15.2	75	69		45	
757			10.425	196.095				
758			5					
759							45	
760			12.26667			70		
761	249.96					30		
762			14.4		48			65
763			14.55	16	20.6	170	88.25	
764			2.815				75	
765			12					
766			12					
767	307.245		2.25		30		40	130
768			10					81.25
769	249.96							55
770			12					
771							115	165
772	129.76				16		115	140
773		519.99						
774							45	
775				44			60	148
776			3.75					100
777		649.99	8	62.5		140		
778			17.33333					200

779			14.4					80
780	374.94				49.38	21	16	
781	249.96		6			60		
782	249.96						50	
783	199.32		7.5	24			90	
784	125.12							
785		520						
786		519.99						
787	249.15		12	26.88				
788	149.49		8.2	57.06			31.91667	
789	175.16							
790			12	60		210	84	
791		25	2				59	
792			13		24	17.08	22.77333	100
793	209.3533	260	16	60				
794	350.32		9.6				30	
795	249.96	242.6	11.08	180			32.25	72.5
796			4				50	76.66666
797			9.333333		32		20	70
798			3				120	150
799	269.9133		14.54	66.875				
800			7.2				132	40
801		234	8				300	
802	149.98		2.4			150		56.66667
803	137.235		4.483334	152	14		50	40
804	74.99			76			5	40
805	280.8		3.933333		19.5		58.33333	65
806			7.2					
807			2.25					
808			12	70				230
809			11.21		24			92.31
810		460.28		120.14	15	60	80	40
811			15				15	
812							100	24
813							150	35
814	149.98		24.3			420	60	
815	247.075		20		30		25	95
816			3		26.66667		40	97.5
817		779.98	12	120				40
818				50				26.4
819	394.7567		14		5		27.46667	

820			5			15	24.75	102
821	216.76			15			30	
822	368.53		4	50.26				
823	253.735			30	15.66667	39.75	37.5	55
824			4.8		20		36.15	100
825		520.58	12.4	280			360	41.66667
826			5.25	219.12	16		19.66667	70
827			18					
828	364.53	226.085		201.17			100	
829	155.72			33.125				
830			19		15	355	150	80
831				110			175	100
832								
833				16		140		95
834			8	22.5	8		13.32	50
835			6.8		12		48.66333	
836							39.84	240
837			8				125	16
838				45.07	20		50	
839	190.075		10		23	117.5		
840	499.92		10		400	94	51.6	
841	86.76		13.6	63	30	35		65
842					22		46.66667	80
843			9		5			
844			17.5	30			250	230
845			24				30	200
846	288.26						75	200
847		259.81	8.875	7.5	16			
848		227.5	4.2	30	30		171.26	
849			7.85	10	30	85	60	
850	199.32		16.8	157.44	59.25			130.6
851	298.98		6.9		50			
852								
853								
854		77.5			17.5			
855	125		10.75			56.25		
856			4.5		39	175		40
857			5.7					50
858	498.3			10	20			35
859							36	
860		151.6667					40	

861	249.96	390.09	10.75	69.98			34	
862		325.09	8.6	38.115			31	
863	240.22		9.6	20		80		95
864			12				16	
865	311.435		7.8				45	150
866			15	260	100	140	100	80
867				100	100			
868			2.25					
869	520.75		5					
870	612.49			37.5				
871						290		
872				60			100	
873						106.5		
874	498.3		24.5				60	37.5
875		350					80	
876	499.92		9.6					
877			12	150				60
878							45	
879	298.98		3.6					
880	218.005		8.8					
881	249.96		12.4	30	20		78	
882	299.225		7.2	66.2	30		100	
883		256.8667	29	105				40
884		324.3175	5.425	32.7		140		40
885			21.6				46.57333	
886			6			140	112.5	
887			12.53				120	
888			7.5					
889			9	80			36	
890	624.9						58.6	
891	171.85		12				43.78	
892			11.7					100
893			10.8					100
894				10		160	130.66	
895	199.565		8	38		120	44	79.22
896			6					
897	249.96		12					
898			6				60	57.5
899	249.555	260	2.725	110	23	30	106	93
900	149.98		18				150	
901			4.565					100

902			4					150
903			12.33333				27	
904	200.18		4					
905			4					
906			4.333333		50			40
907			3.883333		20	18	34	
908	244.98		21.33333	85	15	140	30	475.63
909					30		26.66667	
910		395.46	9.6				30	40
911		395.46	12				30	40
912			4		15		122.5	
913	299.95					100	45	40
914			18		30		82.5	
915	302.04				98.5		35	210
916							163.3333	120
917	364.53							75
918	249.96							
919							240	
920	363.34		12		30			
921	298.98			40			100	
922			14.4				48	
923			10.66667	83	15	21	21	
924					29.66667			
925			12	90.18	24	45	39.18833	16.25
926						145	18	60
927			10.5	62	40	53.33333	96	40
928		520.35						65
929			2				102.5	
930	255.17		6.5	68.75		140		80
931	416.6				30		10	180
932					20			100
933			10.4		20	50		
934	249.96	650	5.425				18	
935			6.625				120.4	100
936			12.5		16		90	300
937	249.96		21		40		95	180
938			19.2		10		175	185
939	149.49		5.083333					160
940			12	50		70		30
941			12	54		157.5		
942			9	65			30	142.5

943				80	30			
944	149.98		3.75	18				
945			18		100			
946		519.99	10.55				111.8333	50
947	240.22			11	25	13.5	98.5	90
948	208.3			42.255				
949			5.733333			45		
950			12					
951			24		30			
952			50		20		100	
953	249.15	390						
954		390					45	
955					24		87.5	280
956						40	48	100
957		200	5.76		18		18.33	60
958			2.375				32	100
959	363.34		8		100	433.3333	50	110
960			6.375			26.25	25	
961			108				360	
962								
963			27		65			
964			9.3125	67.5		107.5	49.125	65
965	249.15		23.48	200		105.25	28.5	80
966		160.41	13.2	24.14			69	40
967	249.96		5.56	18.33333			15	
968	208.3		10.41667	47.5	26			
969	124.57	312	5.625				45	
970	104.15	325	5.75	65			23.75	
971			7.25					
972								
973			18					
974	380.15		5.15				33.75	187.55
975			12.5125	90	31.2	188		110
976			4		39		166.6667	100
977			7.466667		16		80	100
978	374.94		9.75			42.6	45	65
979	165.8		14.4		25	70	60	65
980		260	4.8		25			40
981	498.3		24	213.03				
982			3.6			175		
983		278.25	12.525				25	

984	104.15	321.59	10.8	190				
985							45	
986			9.6				60	
987			2.25				60	
988			9	50				
989			8.35					
990			9.6					
991							60	45
992							75	100
993			15		24.5			45
994			6.05		80			65
995			16					
996	143.0567		3.7		37.5			57.6
997	249.96		10.75	90			48	
998			33.6	150		60	145.75	10
999			5.55		48			65
1000			9.6		24		100	
1001								180
1002								80
1003			19.8		55	138.6	310	
1004			9.416667		115			120
1005			4.5					
